# *In Vivo* Transcriptional Profiling of *Listeria monocytogenes* and Mutagenesis Identify New Virulence Factors Involved in Infection

**DOI:** 10.1371/journal.ppat.1000449

**Published:** 2009-05-29

**Authors:** Ana Camejo, Carmen Buchrieser, Elisabeth Couvé, Filipe Carvalho, Olga Reis, Pierre Ferreira, Sandra Sousa, Pascale Cossart, Didier Cabanes

**Affiliations:** 1 IBMC - Instituto de Biologia Molecular e Celular, Group of Molecular Microbiology, Universidade do Porto, Porto, Portugal; 2 Institut Pasteur, UP Biologie des Bactéries Intracellulaires and CNRS URA 2171, Paris, France; 3 Institut Pasteur, Unité Génétique des Génomes Bactériens CNRS URA 2171, Paris, France; 4 Institut Pasteur, Unité des Interactions Bactéries-Cellules, Paris, France; 5 Inserm U604, Paris, France; 6 INRA USC2020, Paris, France; Yale University School of Medicine, United States of America

## Abstract

*Listeria monocytogenes* is a human intracellular pathogen able to colonize host tissues after ingestion of contaminated food, causing severe invasive infections. In order to gain a better understanding of the nature of host–pathogen interactions, we studied the *L. monocytogenes* genome expression during mouse infection. In the spleen of infected mice, ≈20% of the *Listeria* genome is differentially expressed, essentially through gene activation, as compared to exponential growth in rich broth medium. Data presented here show that, during infection, *Listeria* is in an active multiplication phase, as revealed by the high expression of genes involved in replication, cell division and multiplication. *In vivo* bacterial growth requires increased expression of genes involved in adaptation of the bacterial metabolism and stress responses, in particular to oxidative stress. *Listeria* interaction with its host induces cell wall metabolism and surface expression of virulence factors. During infection, *L. monocytogenes* also activates subversion mechanisms of host defenses, including resistance to cationic peptides, peptidoglycan modifications and release of muramyl peptides. We show that the *in vivo* differential expression of the *Listeria* genome is coordinated by a complex regulatory network, with a central role for the PrfA-SigB interplay. In particular, *L. monocytogenes* up regulates *in vivo* the two major virulence regulators, PrfA and VirR, and their downstream effectors. Mutagenesis of *in vivo* induced genes allowed the identification of novel *L. monocytogenes* virulence factors, including an LPXTG surface protein, suggesting a role for S-layer glycoproteins and for cadmium efflux system in *Listeria* virulence.

## Author Summary

The facultative intracellular bacterial pathogen *Listeria monocytogenes* is the etiological agent of a severe foodborne disease. In humans it causes a variety of manifestations ranging from asymptomatic intestinal carriage and gastroenteritis to invasive and disseminated severe diseases. Septicemia, meningoencephalitis, and infection of the foetus in pregnant women are the most serious clinical features of listeriosis. Virulence is a trait that only manifests in a susceptible host, involving a highly coordinated interaction between bacterial factors and host components. This article reports the use of *in vivo* genome expression profiling as a powerful approach to gain a detailed understanding of the *Listeria* responses and the molecular cross-talk taking place in infected mice. We showed that, during infection, *L. monocytogenes* shifts the expression of its entire genome to promote virulence, subverting host defenses and adapting to host conditions. This first analysis of *L. monocytogenes* gene expression *in vivo* significantly enhances our understanding of the means by which intracellular pathogens promote infection.

## Introduction

*Listeria monocytogenes* is an intracellular food-borne pathogen that causes listeriosis, an infection characterized by gastroenteritis, meningitis, encephalitis, and maternofetal infections in humans. It has one of the highest fatality rate among food-borne infections (20%–30%) [1]. Our knowledge of the infectious process *in vivo* mostly derives from infections in various animal models, in particular the mouse model. It is considered that bacteria after crossing the intestinal barrier reach, via the lymph and the blood, the liver and the spleen where they replicate actively. Then, bacteria via hematogenous dissemination, can reach the brain and the placenta. The disease is thus due to the original property of *L. monocytogenes* to be able to cross three host barriers: the intestinal barrier, the blood brain barrier and the materno-fetal barrier. It is also due to the capacity of *Listeria* to resist intracellular killing when phagocytosed by macrophages and to invade many non-phagocytic cell types. In the murine model, within minutes after intravenous inoculation, most bacteria can be found in the spleen and the liver [2].

*L. monocytogenes* ranks among the best-known intracellular pathogens and, until now, 50 genes have been shown to be involved in virulence in the mouse model (Table S1). However, whereas the different steps of the cell infectious process and the virulence factors specifically involved are well described [3], our knowledge of the *in vivo* infectious process is still fragmentary. Virulence is by definition expressed in a susceptible host, and involves a dynamic cross talk between the host and the pathogen. A detailed understanding of this interaction thus requires global approaches in the context of an *in vivo* infection. Analysis of the pathogen whole genome expression within the host should allow the identification of new bacterial genes critical for the infectious process, and lead to a better understanding of the molecular events responsible for *Listeria* infection.

The technology of DNA arrays allows to both study the gene content of different strains and measure gene expression levels on a genome-wide scale under different conditions. The genetic basis of *L. monocytogenes* pathogenicity was addressed by comparative genomics [4] and transcriptomics [5] using *Listeria* DNA arrays and various *L. monocytogenes* strains. *Listeria* arrays were also used for the analysis of the *in vitro* global gene expression of *Listeria* mutants for PrfA, the central regulator of virulence genes [6], and for other transcriptional regulators important for stress response and virulence (σB, σ54, HrcA, CtsR, VirR) [7]–[13]. Recently, this approach was applied to the determination of the intracellular gene expression profile of *L. monocytogenes* in epithelial and macrophage cell lines [14],[15]. *In vivo* genome profiles of other pathogens (*Streptococcus pneumoniae*, *S. pyogenes*, *Mycobacterium tuberculosis*, *Borrelia burgdoferi*, *Yersinia pestis*) infecting different mouse organs (dermis, soft tissue, lung, blood) were previously performed [16]. However, to our knowledge, the genome expression of a pathogen was never studied in infected mouse spleen.

Here, we present the first “*in vivo*” transcriptome of *L. monocytogenes*. We compared expression profiles of *L. monocytogenes* grown in standard culture medium in exponential phase vs. bacteria recovered from mouse spleens 24, 48 and 72 hours after intravenous infection. We determined the detailed expression kinetics of the complete *L. monocytogenes* genome in the course of the infection, and identified new *Listeria* virulence factors whose expression was highly up regulated *in vivo*.

## Results

### The *in vivo* transcriptome approach

We used the DNA macroarray technology to profile the transcriptome of *Listeria* during mouse infection. We used the previously described *L. monocytogenes* whole-genome arrays containing 500-bp-long PCR products specific for each gene [6]. Ninety-nine per cent of the 2853 predicted ORFs of the *L. monocytogenes* EGDe genome are represented on the arrays. They were used to analyze *Listeria* transcription profiles under *in vitro* growth in BHI in exponential phase at 37°C under aerobic conditions with shaking (pH 7) (Figure S1), and under *in vivo* growth conditions (mouse spleen) at 1, 2 and 3 days post intravenous infection (p.i.). *Listeria* present in spleen were analyzed because this organ is with the liver one of the major sites of *L. monocytogenes* infection. For unknown reasons, we never succeeded to prepare good quality bacterial RNAs from infected mouse livers. The time points chosen reflect key steps in the *Listeria* infectious process.

**Figure 1:**
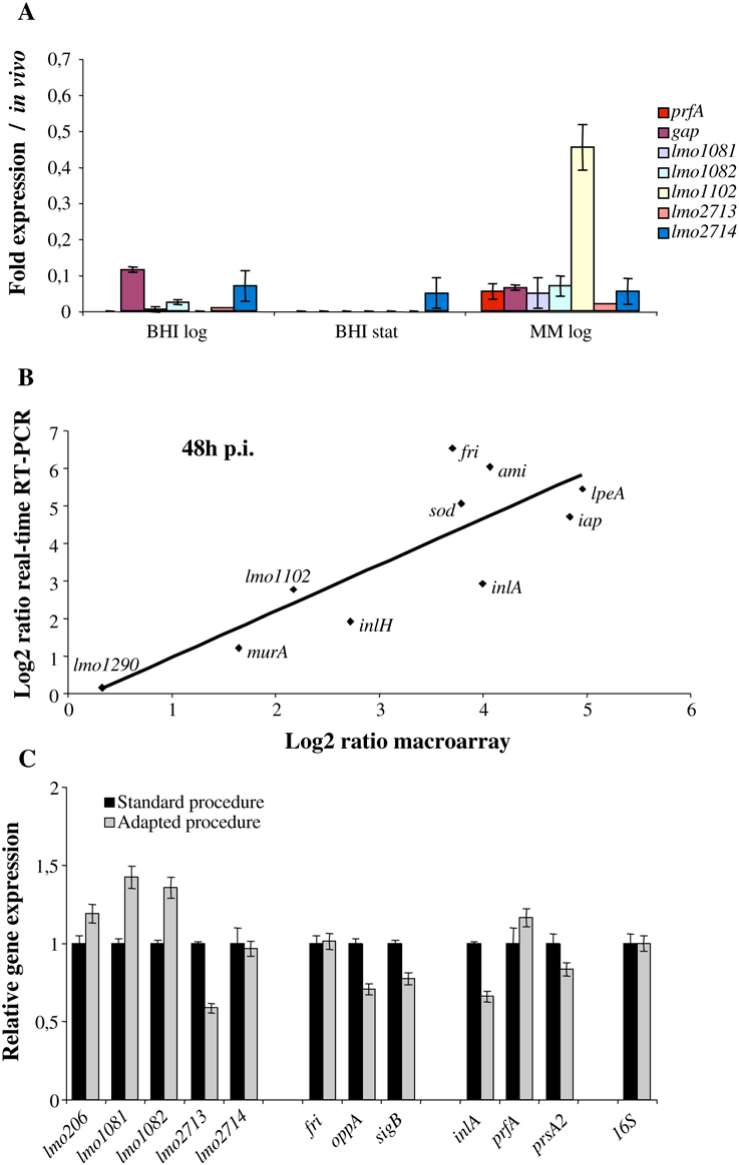
Macroarray validations. (A) Analysis of the impact of the *in vitro* culture conditions used as reference. The expression of known and potential virulence factors was analyzed in BHI at 37°C in exponential (BHI log) or stationary (BHI stat) growth phase, or in minimal medium in exponential growth phase (MM log) by real-time RT-PCR, and normalized to expression in mouse spleen. (B) Validation of macroarray data by real-time RT-PCR. Fold changes in *in vivo* gene expression 48 h p.i. compared to that in BHI were measured by macroarray and real-time RT-PCR, log transformed and compared for correlation analysis. (C) Analysis of the effect of the RNA extraction method on *L. monocytogenes* gene expression. RNAs from bacteria grown in BHI were prepared using the standard and adapted procedures for RNA extraction. The relative expression of potential virulence genes, cold shock genes and known virulence genes was determined by real-time RT-PCR.

Culture in BHI in exponential phase at 37°C with shaking was chosen as reference conditions because BHI is the *Listeria* reference growth medium where bacteria divide in exponential growth phase at rates that are comparable to those observed for intracellular growth [17]. In addition, these are the *in vitro* reference conditions used in all previous studies analyzing the genome expression of *L. monocytogenes in vitro* or intracellularly [6]–[15]. However, in order to analyze the potential impact of the *in vitro* culture conditions used as reference on the relative gene expression *in vivo*, we first analyzed the results obtained comparing transcriptome from *in vivo* grown bacteria to transcriptomes from bacteria grown *in vitro* in exponential or stationary phase (Table S2). In addition, expression of known and potential virulence genes was analyzed by quantitative real time-PCR (qPCR) on RNAs extracted from bacteria cultured in BHI at 37°C in exponential or stationary growth phase, or in defined minimal medium [18], and compared to *in vivo* expression (Figure 1A). Results indicated that culture in exponential growth phase are closer conditions to those met by *Listeria in vivo* (Table S2). In addition, even if the expression of tested genes behaved differently in function of the *in vitro* conditions, expression of all the genes was always lower *in vitro* as compared to *in vivo*, independently of the *in vitro* growth conditions (Figure 1A). These experiments supported the choice of exponential growth phase in BHI as reference conditions and minimized the impact of the *in vitro* growth conditions on the identification of genes differentially expressed *in vivo*.

The reliability of the macroarray expression data was further assessed by qPCR. We selected a subset of 10 genes and performed qPCR on cDNA from bacteria grown in either standard medium or extracted from mouse spleens 48 h p.i.. qPCR results and array data exhibited a high correlation coefficient (0.7) (Figure 1B). This strong correlation was also observed for other infection time points (Figure S2). However, the differences in gene expression, as measured by qPCR, were generally higher, indicating that *in vivo* transcriptome data rather underestimate changes in gene expression.

The procedure used for bacterial RNA extraction from infected mouse spleens is an adaptation of the standard procedure originally used for transcriptional analysis of RNA extracted from pure culture. In order to test the effect of the RNA extraction method on gene expression, RNAs from bacteria grown in pure culture were extracted using the two methods. The relative expression of known virulence genes, cold shock genes and potential virulence genes was analyzed by qPCR in the two RNA pools. The results showed that the relative expression of the genes tested is not significantly affected by the RNA extraction procedure (Figure 1C).

For bacteria cultured in BHI at 37°C in exponential phase or extracted from infected mouse spleen at the different times p.i., two different RNA preparations from independent cultures (or infections) were used for cDNA synthesis and subsequent hybridization to two sets of arrays. To identify statistically significant differences in gene expression, we used the Statistical Analysis for Microarrays (SAM) program [19]. Subsequently, all the genes showing statistically significant changes in the expression level and an at least two-fold change in their level of expression were considered in our analysis.

### Important global changes in *L. monocytogenes* gene expression occur during *in vivo* growth

Overall, a total of 568 genes representing ≈20% of the total genome exhibited a differential expression during infection as compared to growth in BHI at 37°C in exponential phase. Among these 568 genes, 457 were up regulated (≈80%) and 111 (≈20%) were down regulated during mouse infection as compared to exponential growth in BHI medium (Table S3).

In order to identify genes potentially implicated in virulence, all the genes differentially regulated *in vivo* were analyzed for the presence of an ortholog in the nonpathogenic close relative *Listeria innocua* strain CLIP11262 [20]. This analysis revealed that only 30 of the *in vivo* regulated genes (25 up and 5 down regulated) were absent from *L. innocua* (Table 1). Of these 30 genes, 20 were *L. monocytogenes* “specific” (i.e. also present in *L. monocytogenes* 1/2a F6854, *L. monocytogenes* 4b F2365 and H7858 [21], and absent from *L. innocua*). Interestingly, of these 20 genes, 16 were up regulated *in vivo*. Among these 16 genes, 11 have been previously implicated in *Listeria* virulence. The remaining 10 *in vivo* regulated genes, among which 9 up- and 1 down-regulated *in vivo*, appeared lineage specific, i.e. present only in the sequenced serovar 1/2a strains (Table 1).

**Table 1:** *L. monocytogenes* EGDe genes absent from *L. innocua* and differentially regulated in the host.

Gene designation	Gene	Annotation	Homolog in 1/2a F6854	Homolog in 4b F2365	Homolog in 4b H7858	Fold change 24 h	Fold change 48 h	Fold Change 72 h
*prfA*	*lmo0200*	listeriolysin positive regulatory protein	LMOf6854_0209	LMOf2365_0211	LMOh7858_0220		8,09	4,00
*plcA*	*lmo0201*	phosphatidylinositol-specific phospholipase c	LMOf6854_0210	LMOf2365_0212	LMOh7858_0221	7,20	48,31	6,70
*hly*	*lmo0202*	listeriolysin O precursor	LMOf6854_0211	LMOf2365_0213	LMOh7858_0222	35,56	118,39	15,14
*mpl*	*lmo0203*	zinc metalloproteinase precursor	LMOf6854_0212	LMOf2365_0214	LMOh7858_0223	3,36	18,41	4,68
*actA*	*lmo0204*	actin-assembly inducing protein precursor	LMOf6854_0213	LMOf2365_0215	LMOh7858_0224	6,02	15,58	4,49
*plcB*	*lmo0205*	phospholipase C	LMOf6854_0214	LMOf2365_0216	LMOh7858_0225	12,37	106,64	31,78
*lmo0206*	*lmo0206*	unknown protein	LMOf6854_0214.1	LMOf2365_0217	LMOh7858_0225.1			3,11
*lmo0257*	*lmo0257*	unknown protein	LMOf6854_0261.2	LMOf2365_0265	LMOh7858_0288		2,12	
*inlH*	*lmo0263*	internalin H	LMOf6854_0275	LMOf2365_0281	LMOh7858_0295	3,08	6,60	2,41
*inlA*	*lmo0433*	internalin A	LMOf6854_0469	LMOf2365_0471	LMOh7858_0499		15,92	3,86
*inlB*	*lmo0434*	internalin B	LMOf6854_0470	LMOf2365_0472	LMOh7858_0501.2		3,82	2,55
*uhpT*	*lmo0838*	hexose phosphate transport protein	LMOf6854_0883	LMOf2365_0855	LMOh7858_0894		5,13	2,59
*lmo0915*	*lmo0915*	similar to phosphotransferase system enzyme IIC	LMOf6854_0962	LMOf2365_0937	LMOh7858_0989	3,15		2,37
*lmo1290*	*lmo1290*	similar to internalin proteins, putative peptidoglycan bound protein (LPXTG)	LMOf6854_1332	LMOf2365_1307	LMOh7858_1374.2			6,24
*inlC*	*lmo1786*	internalin C	LMOf6854_1844.2	LMOf2365_1812	LMOh7858_1916.1		8,56	3,57
*lmo2157*	*lmo2157*	unknown protein	LMOf6854_2221	LMOf2365_2189	LMOh7858_2290.1	3,29	5,85	2,01
*lmo2257*	*lmo2257*	hypothetical CDS	LMOf6854_2321.1	LMOf2365_2290	LMOh7858_2400	−4,62		−3,99
*lmo2672*	*lmo2672*	weakly similar to transcription regulator	LMOf6854_2788	LMOf2365_2652	LMOh7858_2935			−2,31
*lmo2733*	*lmo2733*	similar to PTS system, fructose-specific IIABC component	LMOf6854_2852	LMOf2365_2720	LMOh7858_2997			−2,35
*lmo2736*	*lmo2736*	unknown protein	LMOf6854_2855	LMOf2365_2723	LMOh7858_3000			−2,48
*lmo1081*	*lmo1081*	similar to glucose-1-phosphate thymidyl transferase	LMOf6854_1134			8,25	5,99	5,17
*lmo1082*	*lmo1082*	similar to dTDP-sugar epimerase	LMOf6854_1135			47,21	24,06	21,01
*lmo1083*	*lmo1083*	similar to dTDP-D-glucose 4,6-dehydratase	LMOf6854_1136				3,49	
*lmo1084*	*lmo1084*	similar to DTDP-L-rhamnose synthetase	LMOf6854_1137				3,89	
*lmo2276*	*lmo2276*	similar to an unknown bacteriophage protein	LMOf6854_2338					−2,72
*lmo0150*	*lmo0150*	unknown protein					16,53	
*lmo0471*	*lmo0471*	unknown protein					3,18	
*lmo1099*	*lmo1099*	similar to a protein encoded by Tn916				65,42	30,42	24,04
*lmo1102*	*lmo1102*	similar to cadmium efflux system accessory proteins				9,71	4,51	4,72
*lmo2277*	*lmo2277*	unknown protein					2,61	

**Figure 2:**
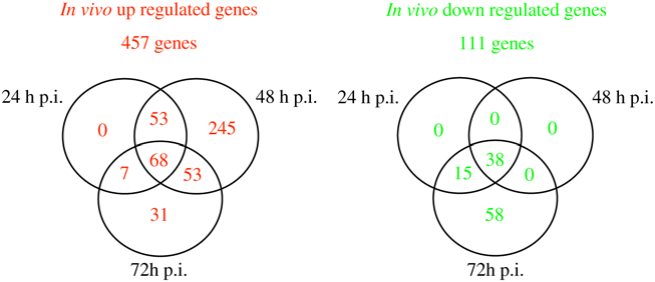
Venn diagrams showing the distribution of the up and down regulated genes at the three *in vivo* infection time points.

To identify genes regulated during different stages of listeriosis, gene expression levels of spleen-recovered bacteria at different time points p.i. were compared. This analysis revealed a core regulon of 106 genes (68 up and 38 down regulated) whose expression was significantly differentially regulated at all the time points of the infection as compared to bacteria grown in pure culture (Figure 2). No gene appeared specifically differentially regulated at 24 h p.i. At two days p.i., a large proportion (245/457) of genes was up regulated. The largest number of down regulated genes was observed 72 h p.i..

### Major virulence regulators and their downstream target genes are highly up regulated *in vivo*

**Figure 3:**
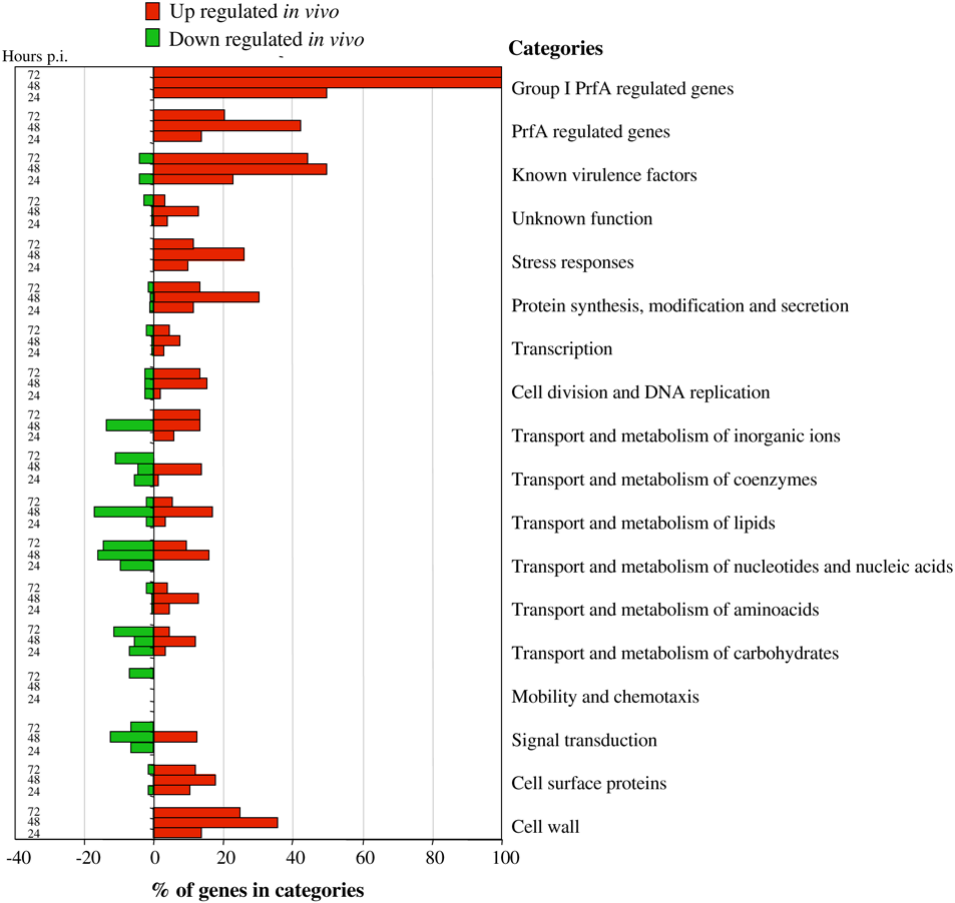
Differentially regulated genes of *L. monocytogenes* EGDe obtained from temporal transcriptome profiling experiments, classified in functional categories.

**Figure 4:**
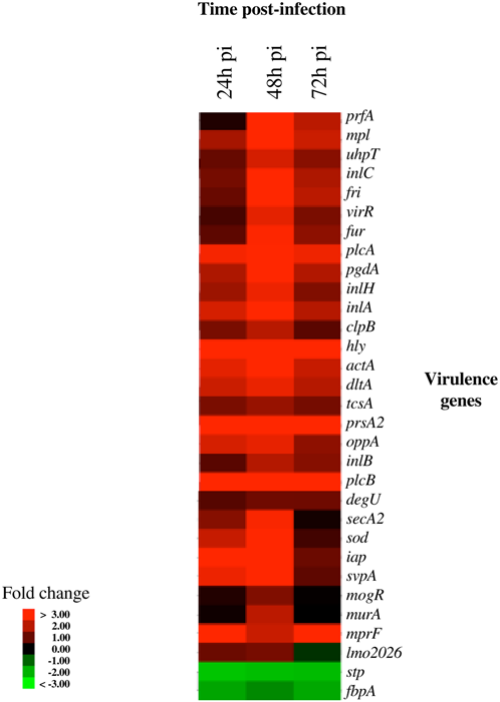
*In vivo* expression of virulence genes. Expression during mouse spleen infection of the 31 known virulence genes differentially regulated *in vivo*. A peak of expression was observed for the majority of these virulence genes 48 h p.i. All measurements are relative to culture in exponential phase in BHI. Genes were selected for this analysis when their expression deviated from BHI by at least a factor of 2.0 in at least one time point. The image was produced as described in Materials and Methods. Each gene is represented by a single row of colored boxes; each time point is represented by a single column.

As compared to *Listeria* grown in BHI at exponential phase, bacteria extracted from mouse spleens showed a differential expression of genes belonging to various functional categories (Figure 3). In particular, analysis of the expression profile of the 50 genes previously implicated in *Listeria* virulence in the mouse model revealed that 29 were up regulated during infection, and two (*stp* and *fbpA*) down regulated *in vivo* (Figure 4 and Table S1).

We observed that the entire virulence gene cluster of *L. monocytogenes* comprising the genes *prfA*, *plcA*, *hly*, *mpl*, *actA* and *plcB* was highly activated during the 3 first days of infection (Table 2). In addition to the virulence gene cluster, genes encoding the two major *L. monocytogenes* factors implicated in entry into eukaryotic cells (*inlA* and *inlB*) [22], and *uhpT*, a gene encoding a sugar phosphate transporter that mediates rapid intracellular proliferation [23] were also activated during infection. PrfA is the principal regulator of the expression of not only these key virulence genes, but also of most other *L. monocytogenes* genes involved in intracellular survival and virulence [6]. The 12 genes previously reported to be preceded by a PrfA box and positively regulated by PrfA in a transcriptional analysis of the PrfA regulon [6], were all highly up regulated in mouse spleens (Table 2). From the 53 other genes already shown as positively regulated by PrfA [6], 20 were more expressed *in vivo*. As previously shown [8], 19 of these 20 genes are also controlled by SigB, including the LPXTG internalin-like protein *inlH* known to be involved in *Listeria* virulence [24]. Two genes, *lmo0206* and *lmo0207*, recently shown as regulated by PrfA and implicated in *L. monocytogenes* intracellular survival [14] were also activated in infected mice. Importantly, no gene previously shown under the PrfA positive regulation appeared down regulated during mouse infection.

**Table 2:** *L. monocytogenes* EGDe genes positively controlled by PrfA and up regulated in the host.

Gene designation	Gene	Annotation	Homolog in *L. innocua*	Fold change 24 h	Fold change 48 h	Fold change 72 h
*Group I PrfA regulated genes*						
*prfA*	*lmo0200*	listeriolysin positive regulatory protein			8,09	4,00
*plcA*	*lmo0201*	phosphatidylinositol-specific phospholipase C		7,20	48,31	6,70
*hly*	*lmo0202*	listeriolysin O precursor		35,56	118,39	15,14
*mpl*	*lmo0203*	zinc metalloproteinase precursor		3,36	18,41	4,68
*actA*	*lmo0204*	actin-assembly inducing protein precursor		6,02	15,58	4,49
*plcB*	*lmo0205*	phospholipase C		12,37	106,64	31,78
*inlA*	*lmo0433*	internalin A			15,92	3,86
*inlB*	*lmo0434*	internalin B			3,82	2,55
*lmo0788*	*lmo0788*	unknown protein	lin0781	60,06	31,36	20,50
*uhpT*	*lmo0838*	hexose phoshate transport protein			5,13	2,59
*inlC*	*lmo1786*	internalin C			8,56	3,57
*prsA2*	*lmo2219*	similar to post-translocation molecular chaperone	lin2322	12,02	24,00	8,95
*Group III PrfA regulated genes co-controlled by SigB*						
*lmo0169*	*lmo0169*	similar to a glucose uptake protein	lin0212		3,11	
*inlH*	*lmo0263*	internalin H		3,08	6,60	2,41
*lmo0439*	*lmo0439*	weakly similar to a module of peptide synthetase	lin0460		2,76	
*lmo0539*	*lmo0539*	similar to tagatose-1,6-diphosphate aldolase	lin0543	18,71	24,03	
*lmo0596*	*lmo0596*	unknown protein	lin0605	23,31	68,15	
*lmo0781*	*lmo0781*	similar to mannose-specific phosphotransferase system (PTS) component IID	lin0774		2,57	
*lmo0782*	*lmo0782*	similar to mannose-specific phosphotransferase system (PTS) component IIC	lin0775		4,69	
*lmo0783*	*lmo0783*	similar to mannose-specific phosphotransferase system (PTS) component IIB	lin0776		3,82	
*lmo0784*	*lmo0784*	similar to mannose-specific phosphotransferase system (PTS) component IIA	lin0777		2,89	
*lmo0794*	*lmo0794*	similar to B. subtilis YwnB protein	lin0787		4,47	
*lmo0796*	*lmo0796*	unknown protein	lin0789		4,21	
*lmo0994*	*lmo0994*	unknown protein	lin0993		2,53	
*opuCD*	*lmo1425*	osmoprotectant transport system permease protein	lin1464		2,69	
*lmo1601*	*lmo1601*	similar to general stress protein	lin1642			2,18
*lmo1602*	*lmo1602*	unknown protein	lin1643		4,54	2,13
*lmo2157*	*lmo2157*	unknown protein		3,29	5,85	2,01
*lmo2391*	*lmo2391*	conserved hypothetical protein similar to B. subtilis YhfK protein	lin2490		4,56	
*lmo2696*	*lmo2696*	similar to hypothetical dihydroxyacetone kinase	lin2844		2,49	
*lmo2697*	*lmo2697*	unknown protein	lin2845		3,14	
*Group III PrfA regulated gene not controlled by SigB*						
*lmo0937*	*lmo0937*	unknown protein	lin0936		3,92	
*Other PrfA regulated genes*						
*lmo0206*	*lmo0206*	unknown protein				3,11
*lmo0207*	*lmo0207*	hypothetical lipoprotein	lin0239		5,31	3,27

VirR, another key *Listeria* virulence regulator that mainly controls genes involved in the modification of bacterial surface components, is the response regulator of a two-component system (TCS) implicated in cell invasion and virulence [13]. Using a transcriptomic approach, 17 genes were previously identified as regulated by VirR *in vitro* [13]. In our study, 13 of the 17 VirR regulated genes, including the *dlt* operon and *mprF*, were up regulated *in vivo* (Table 3). The *dlt* operon is necessary for D-alanylation of lipoteichoic acid (LTA) and was reported to be important for *L. monocytogenes* virulence [13]. The VirR regulated *mprF* encodes a protein shown to be required for lysinylation of phospholipids in the *Listeria* cytoplasmic membrane and to confer *Listeria* resistance to cationic antimicrobial peptides (CAMPs) [25]. The *virR* and *virS* genes were themselves up regulated, constituting the only TCS whose expression of both components was induced in mouse spleens.

**Table 3:** *L. monocytogenes* EGDe genes regulated by VirR and up regulated in the host.

Gene designation	Gene	Annotation	Homolog in *L. innocua*	Fold change 24 h	Fold change 48 h	Fold change 72 h
*lmo0604*	*lmo0604*	similar to B. subtilis YvlA protein	lin0613	51,45	27,43	
*dltD*	*lmo0971*	DltD protein for D-alanine esterification of lipoteichoic acid and wall teichoic acid	lin0970, dltD	11,98	21,95	3,73
*dltC*	*lmo0972*	D-alanine–poly(phosphoribitol) ligase subunit 2 DltC	lin0971, dltC	4,13	6,22	2,35
*dltB*	*lmo0973*	DltB protein for D-alanine esterification of lipoteichoic acid and wall teichoic acid	lin0972, dltB	6,48	7,58	2,73
*dltA*	*lmo0974*	D-alanine–D-alanyl carrier protein ligase DltA	lin0973, dltA		6,37	3,85
*mprF*	*lmo1695*	similar to MprF protein of S aureus	lin1803	9,68	4,59	24,61
*virS*	*lmo1741*	two-component sensor histidine kinase	lin1852		2,76	
*adeC*	*lmo1742*	highly similar to adenine deaminases	lin1853		2,66	
*virR*	*lmo1745*	two-component response regulator	lin1856		5,96	2,29
*lmo2114*	*lmo2114*	similar to ABC transporter (ATP-binding protein)	lin2219		3,68	2,93
*lmo2115*	*lmo2115*	similar to ABC transporter (permease)	lin2220	4,86	5,92	3,06
*lmo2177*	*lmo2177*	unknown protein	lin2280	3,36	6,14	2,23
*lmo2439*	*lmo2439*	unknown protein	lin2533		4,44	

In addition to VirRS, the *L. monocytogenes* genome contains 15 additional predicted TCS systems [26]. Genes encoding one component of three of these TCS (*degU*, *resD* and *phoR*) were also up regulated *in vivo*. DegU is an orphan response regulator (absence of the sensor kinase DegS in the *L. monocytogenes* genome) and a pleiotropic regulatory system previously involved in *Listeria* virulence [27],[28]. In particular, DegU has been implicated in the regulation of some *Listeria* secreted proteins (*gap*, *tsf*, *sod*, *lmo0644*) [26]. Interestingly, the expression of these four genes was also increased in mouse spleens.

Finally, OhrR a transcriptional regulator controlling OhrA, a hydroxyperoxidase implicated in intracellular survival of *Listeria* [14], as well as several predicted transcriptional regulators were up regulated in infected mouse spleens.

### Strong activation of genes encoding cell wall metabolism proteins during infection

In addition to genes already mentioned and involved in LTA modification (*dltABCD*), we observed that several genes implicated in peptidoglycan (PG) biosynthesis (*lmo0516*, *lmo0540*, *lmo1438*, *lmo1521*, *lmo1855*, *lmo2522*, *lmo2526 and pbpB*), cell shape determination (*mreBC*, *lmo1713*), cell wall peptide synthesis (*murC*) were up regulated in bacteria growing in mouse spleens (Table 4).

**Table 4:** *L. monocytogenes* EGDe genes implicated in cell wall metabolism and differentially regulated in the host.

Gene designation	Gene	Annotation	Homolog in *L. innocua*	Fold change 24 h	Fold change 48 h	Fold change 72 h
*pgdA*	*lmo0415*	peptidoglycan N-acetylglucosamine deacetylase A	lin0436		14,32	3,71
*lmo0516*	*lmo0516*	similar to Bacillus anthracis encapsulation protein CapA	lin0516		14,22	
*lmo0540*	*lmo0540*	similar to penicillin-binding protein	lin0544	3,78	3,97	2,28
*iap*	*lmo0582*	P60 extracellular protein, invasion associated protein Iap	lin0591	34,54	28,64	2,04
*dltD*	*lmo0971*	DltD protein for D-alanine esterification of lipoteichoic acid and wall teichoic acid	lin0970, dltD	11,98	21,95	3,73
*dltC*	*lmo0972*	D-alanine–poly(phosphoribitol) ligase subunit 2 DltC	lin0971, dltC	4,13	6,22	2,35
*dltB*	*lmo0973*	DltB protein for D-alanine esterification of lipoteichoic acid and wall teichoic acid	lin0972, dltB	6,48	7,58	2,73
*dltA*	*lmo0974*	D-alanine–D-alanyl carrier protein ligase DltA	lin0973, dltA		6,37	3,85
*lmo1075*	*lmo1075*	similar to teichoic acid translocation ATP-binding protein TagH (ABC transporter)	lin1063	9,25	5,46	4,20
*lmo1081*	*lmo1081*	similar to glucose-1-phosphate thymidyl transferase		8,22	5,98	5,17
*lmo1082*	*lmo1082*	similar to dTDP-sugar epimerase		47,18	24,08	20,97
*lmo1083*	*lmo1083*	similar to dTDP-D-glucose 4,6-dehydratase			3,51	
*lmo1084*	*lmo1084*	similar to DTDP-L-rhamnose synthetase			3,89	
*lmo1291*	*lmo1291*	similar to acyltransferase (to B. subtilis YrhL protein)	lin1329		4,72	
*uppS*	*lmo1315*	similar to undecaprenyl diphosphate synthase	lin1352			2,16
*lmo1438*	*lmo1438*	similar to penicillin-binding protein	lin1477		3,56	
*lmo1521*	*lmo1521*	similar to N-acetylmuramoyl-L-alanine amidase	lin1556		2,73	
*mreC*	*lmo1547*	similar to cell-shape determining protein MreC	lin1581		2,45	
*mreD*	*lmo1548*	similar to cell-shape determining protein MreB	lin1582		4,08	
*murC*	*lmo1605*	similar to UDP-N-acetyl muramate-alanine ligase	lin1646			2,39
*mprF*	*lmo1695*	multiple peptide resistance factor	lin1803	9,71	4,59	24,59
*lmo1713*	*lmo1713*	similar to cell-shape determining protein MreB	lin1825		5,82	
*lspA*	*lmo1844*	signal peptidase II	lin1958			−2,01
*lmo1851*	*lmo1851*	similar to carboxy-terminal processing proteinase	lin1965			−2,12
*lmo1855*	*lmo1855*	similar to similar to D-alanyl-D-alanine carboxypeptidases	lin1969		3,32	
*pbpB*	*lmo2039*	penicilin binding protein 2B	lin2145		5,00	
*srtB*	*lmo2181*	sortase B	lin2285		2,44	
*svpA*	*lmo2185*	unknown protein	lin2289	6,61	11,11	
*lmo2186*	*lmo2186*	unknown protein	lin2290		2,72	
*lmo2203*	*lmo2203*	similar to N-acetylmuramoyl-L-alanine amidase and to internalin B	lin2306		4,50	
*prsA2*	*lmo2219*	similar to post-translocation molecular chaperone	lin2322	12,02	24,00	8,95
*spl*	*lmo2505*	peptidoglycan lytic protein P45	lin2648, spl		12,21	2,68
*lmo2522*	*lmo2522*	similar to hypothetical cell wall binding protein from B. subtilis	lin2666		5,74	3,07
*lmo2526*	*lmo2526*	UDP-N-acetylglucosamine 1-carboxyvinyltransferase	lin2670			2,40
*murA*	*lmo2691*	similar to autolysin, N-acetylmuramidase	lin2838		4,06	

The expression of 3 genes encoding virulence factors involved in bacterial cell wall modifications (*murA*, *iap*, and *pgdA*) [29]–[31] was also increased *in vivo*. MurA and P60, the *iap* gene product, are two SecA2-secreted autolysins required for *Listeria* full virulence [29],[30]. *pgdA* encodes for the PG N-deacetylase of *L. monocytogenes* that was demonstrated as playing an important role in virulence and evasion from host defenses [31]. In addition, *spl* [32] and *lmo2203* are two other autolysins encoding genes up regulated *in vivo*, but until now never implicated in virulence.

Moreover, *prsA2*, a gene encoding a surface protein involved in protein folding and previously shown as implicated in *Listeria* intracellular survival and virulence [14],[33] was up regulated *in vivo*. Interestingly, the gene encoding the sortase SrtB that covalently links proteins to the *Listeria* peptidoglycan, and two genes encoding SrtB substrates (*svpA* and *lmo2186*) [34], were also over expressed *in vivo* (Table 4).

### Differential expression of genes encoding specific surface and secreted proteins during infection

Whereas a total of 44 genes encoding potential surface proteins were up regulated *in vivo*, only three were observed as down regulated during infection (*lspA*, *lmo1851* and *lmo2642*) (Table 5). In addition, among the 55 proteins previously identified in the cell wall subproteome of *L. monocytogenes* [35], we found that 23 were up regulated *in vivo* (Table S4). The *L. monocytogenes* genome encodes 41 LPXTG surface proteins [20],[36],[37]. This class includes proteins containing leucine rich repeats (LRRs) and belonging to the internalin family. Four LPXTG-protein encoding genes were up regulated *in vivo*. In addition to InlA and InlH, *lmo1290 and lmo2714* are the two other LPXTG encoding genes activated during infection (Table 5). Four genes encoding proteins associated to the cell wall via GW modules were also more expressed *in vivo*: *inlB*, the known invasion protein [38], and *lmo1521*, *lmo2203 and lmo2713*. *actA* [39] was the only gene encoding a protein with a carboxyl-terminal hydrophobic tail up regulated *in vivo*. Genes encoding lipoproteins previously implicated in *Listeria* virulence (TcsA and OppA) [33],[40] or in cell invasion (LpeA) [41], were over expressed in mouse spleens. In addition, 10 genes predicted to encode other lipoproteins were activated *in vivo* (Table 5).

**Table 5:** *L. monocytogenes* EGDe cell surface encoding genes differentially regulated in the host.

Gene designation	Gene	Annotation	Homolog in *L. innocua*	Fold change 24 h	Fold change 48 h	Fold change 72 h
**Sortase substrates: LPXTG and NXXTX proteins**						
*inlH*	*lmo0263*	internalin H		3,07	6,59	2,41
*inlA*	*lmo0433*	internalin A			15,89	3,86
*lmo1290*	*lmo1290*	similar to internalin proteins, putative peptidoglycan bound protein (LPXTG motif)				6,23
*svpA*	*lmo2185*	unknown protein	lin2289	6,61	11,11	
*lmo2186*	*lmo2186*	unknown protein	lin2290		2,72	
*lmo2714*	*lmo2714*	peptidoglycan anchored protein (LPXTG motif)	lin2862	25,46	70,03	3,10
**Proteins with noncovalent association to cell wall**						
*inlB*	*lmo0434*	internalin B			3,84	2,55
*iap*	*lmo0582*	P60 extracellular protein, invasion associated protein Iap	lin0591, iap	34,64	28,54	2,05
*lmo1521*	*lmo1521*	similar to N-acetylmuramoyl-L-alanine amidase	lin1556		2,73	
*murC*	*lmo1605*	UDP-N-acetylmuramate–L-alanine ligase	lin1646			2,40
*lmo1851*	*lmo1851*	similar to carboxy-terminal processing proteinase	lin1965			−2,12
*lmo2203*	*lmo2203*	similar to N-acetylmuramoyl-L-alanine amidase	lin1556		4,50	
*lmo2522*	*lmo2522*	similar to hypothetical cell wall binding protein from B. subtilis	lin2666		5,74	3,07
*lmo2713*	*lmo2713*	secreted protein with 1 GW repeat	lin2861		17,03	2,79
**Proteins with an hydrophobic tail**						
*actA*	*lmo0204*	actin-assembly inducing protein precursor		6,02	15,56	4,50
**Lipoproteins**						
*qoxA*	*lmo0013*	AA3-600 quinol oxidase subunit II	lin0013		2,95	
*lmo0153*	*lmo0153*	similar to a probable high-affinity zinc ABC transporter (Zn(II)-binding lipoprotein)	lin0191		5,31	
*lmo0207*	*lmo0207*	hypothetical lipoprotein	lin0239		5,31	3,27
*lmo0303*	*lmo0303*	putaive secreted, lysin rich protein	lin0331		3,18	
*lmo0355*	*lmo0355*	similar to Flavocytochrome C Fumarate Reductase chain A	lin0374	2,97	6,68	2,51
*lmo0366*	*lmo0366*	putative lipoprotein	lin0385		2,69	2,17
*prs*	*lmo0509*	similar to phosphoribosyl pyrophosphate synthetase			2,87	
*lmo0541*	*lmo0541*	similar to ABC transporter (binding protein)	lin0545	6,19	10,27	2,07
*tcsA*	*lmo1388*	CD4+ T cell-stimulating antigen, lipoprotein	lin1425			2,19
*lmo1649*	*lmo1649*	unknown protein	lin1689		3,39	
*lpeA*	*lmo1847*	similar to adhesion binding proteins and lipoproteins	lin1961	6,54	31,12	10,63
*lmo2184*	*lmo2184*	similar to ferrichrome ABC transporter (binding protein)	lin2288		5,10	2,57
*oppA*	*lmo2196*	similar to pheromone ABC transporter (binding protein)	lin2300	5,21	6,15	2,73
*lmo2642*	*lmo2642*	unknown protein	lin2791			−2,13
**Surface proteins involved in cell wall metabolism**						
*pgdA*	*lmo0415*	peptidoglycan N-acetylglucosamine deacetylase A	lin0436		14,32	3,71
*lmo0540*	*lmo0540*	similar to penicillin-binding protein	lin0544	3,78	3,97	2,28
*iap*	*lmo0582*	P60 extracellular protein, invasion associated protein Iap	lin0591	34,54	28,64	2,04
*lmo1438*	*lmo1438*	similar to penicillin-binding protein	lin1477		3,56	
*lmo1521*	*lmo1521*	similar to N-acetylmuramoyl-L-alanine amidase	lin1556		2,73	
*lmo1855*	*lmo1855*	similar to similar to D-alanyl-D-alanine carboxypeptidases	lin1969		3,32	
*pbpB*	*lmo2039*	penicilin binding protein 2B	lin2145		5,00	
*lmo2203*	*lmo2203*	similar to N-acetylmuramoyl-L-alanine amidase and to internalin B	lin2306		4,50	
*spl*	*lmo2505*	peptidoglycan lytic protein P45	lin2648, spl		12,21	2,68
*lmo2522*	*lmo2522*	similar to hypothetical cell wall binding protein from B. subtilis	lin2666		5,74	3,07
*murA*	*lmo2691*	similar to autolysin, N-acetylmuramidase	lin2838		4,06	
**Surface proteins involved in protein processing, folding and cell surface anchoring**						
*lspA*	*lmo1844*	signal peptidase II	lin1958, lspA			−2,01
*lmo1851*	*lmo1851*	similar to carboxy-terminal processing proteinase	lin1965			−2,12
*srtB*	*lmo2181*	sortase B	lin2285		2,44	
*prsA2*	*lmo2219*	similar to post-translocation molecular chaperone	lin2322	12,02	24,00	8,95

Protein secretion is of key importance in both the colonization process and virulence of *Listeria* [42]. Besides *L. monocytogenes* virulence factors with a signal peptide (ActA, LLO, InlA, InlB, InlC, InlH, Mpl, MurA, PlcA, PlcB, P60 and SvpA), three other virulence proteins (Fri, TcsA and Sod) were also found secreted in the *Listeria* culture supernatant [43]. All the genes encoding these secreted virulence factors appeared activated in our *in vivo* approach (Table S5). The analysis of the products present in the *Listeria* culture supernatant after growth *in vitro* allowed the identification of 89 additional proteins [43]. 29 of the genes encoding these secreted proteins were up regulated *in vivo* (Table S5). Most of the *Listeria* secreted proteins are presumed to be secreted through the Sec translocation system. A gene encoding one component of the predicted Sec system, *secE*, was observed up regulated *in vivo*. SecA2 is an auxiliary secretory protein required for persistent colonization of host tissues, and responsible for the secretion of several *Listeria* virulence factors (MurA, P60, Sod, OppA and TcsA) [29],[30],[44]. We observed an *in vivo* up regulation of the majority of the genes encoding SecA2-secreted proteins, including all the SecA2-secreted virulence factors (Table S5).

### *In vivo* high expression of genes involved in DNA metabolism, RNA and protein synthesis, cell division and multiplication

We observed an *in vivo* up regulation of several genes involved in DNA synthesis (*dnaX* and *lmo0162*), DNA restriction/modifications and repair (*mutL*, *uvrB*, *lmo1639* and *lmo1782*), DNA recombination (*recFRX*, *codV* and *lmo2267*), and DNA packaging and segregation (*gyrA*, *hup*, *lmo1606* and *lmo2794*) (Table S6). In addition, the expression of 25 genes encoding ribosomal proteins, as well as genes involved in protein synthesis initiation (*infAC*), elongation (*fus*, *tsf*, *lmo1067*) and termination (*frr*) was up regulated during infection. Genes encoding proteins implicated in chromosomal replication and segregation (*dnaABC*, *ssb* and *divIVA*), and cell elongation and division (*mreBC*, *ftsHX* and *lmo0196*) were also up regulated in mouse spleens (Table S6).

### Induction of genes implicated in stress responses during infection

In our study, genes belonging to the three principal classes of stress genes were up regulated in the host. Class I genes encode classical chaperones and are controlled by the HrcA repressor. Nine of the 25 genes previously shown as HrcA repressed [11] were activated *in vivo*, including genes encoding the molecular chaperones DnaK and GroEL respectively also shown as induced in macrophages and required for survival following phagocytosis [45],[46] (Table S7). Inversely, 17 of the 36 genes shown to be indirectly positively regulated by HrcA [11], were up regulated in mouse spleens. This list includes genes encoding ribosomal proteins, as well as a number of DNA replication, transcription or translation related genes.

The class II stress response is mediated by sigma B (SigB). A total of 30 genes that have been recently classified as SigB activated [8] appeared here up regulated *in vivo* (Table S7). In particular we detected the up regulation of *inlH* [24], *ltrC* implicated in response to cold shock [47], and *lmo1601* similar to general stress proteins. Interestingly, 40 genes previously classified as down regulated by SigB during the stationary growth phase [8] were detected as activated *in vivo* (Table S7). These include *kat*, a catalase involved in the oxidative stress response [48], a large proportion of genes encoding ribosomal proteins or implicated in translation, cell division and cell wall biogenesis. Furthermore, *iap*, the P60 gene [29], is part of this group. Finally, *rsbU* and *rsbX*, two components of the complex regulation system of SigB [49] were also up regulated in mouse spleens (Table S7).

CtsR is a transcriptional repressor involved in the control of class III stress proteins and previously shown to be responsible for the repression of 42 genes [12], 15 of which appeared up regulated in the host (Table S7). In particular, CtsR regulates the expression of Clp proteases required for the degradation of abnormal proteins and implicated in bacterial escape from macrophage vacuoles and virulence in mice [50]. Expression of *clpBCE* was activated during infection, as well as *mcsA* and *mcsB* the modulators of the CtsR regulon.

In some host cells, bacteria are confronted with severe oxidative stress due to the release of reactive oxygen intermediates. We observed the *in vivo* activation of an important number of oxidative stress resistance mechanisms. The *qoxABCD* operon that encodes a quinol oxidase important for oxidative stress response, and two major proteins implicated in protection against superoxides and reactive oxygen species (ROS), Kat and Sod, were highly up regulated *in vivo* (Table S7). Sod was previously shown as required for *Listeria* full virulence and is a target of Stp, a serine-threonine phosphatase also involved in *L. monocytogenes* virulence [44],[51], and detected down regulated in the host. A decrease in the level of Stp was previously associated to an increase in phosphorylated Sod, accompanied by the secretion of active non-phosphorylated Sod by the SecA2 system [44],[51]. Furthermore, genes encoding a thioredoxin and two thioredoxin reductases involved in the response to oxidative stress (*lmo2152*, *trxB* and *lmo2390*) were up regulated in our study (Table S7). The ferritin protein Fri, that also provides protection against reactive oxygen species, is essential for virulence and is required for efficient bacterial growth at early stages of the infection process [52],[53]. Fri transcription is directly regulated by Fur, the ferric uptake regulator. The expression of *fri* and *fur* was activated during infection. In addition, *ohrA* and *gap* were up regulated *in vivo* and encode two proteins respectively involved in hydroperoxide resistance [54] and in resistance against reactive oxygen species produced by host phagocytic cells in *Leishmania* [55] (Table S7).

### *L. monocytogenes* metabolism adaptation to *in vivo* conditions

Remarkably, 30% of the *in vivo* regulated genes are involved in *L. monocytogenes* metabolism (99 metabolism-related genes were up and 72 were down regulated) (Table S8). As described above, *uhpT* is an *in vivo* highly up regulated virulence gene, regulated by PrfA and that promotes the uptake of phosphorylated hexoses (glucose-1-phosphate and glucose-6-phosphate) [23],[56]. Phosphorylated glucose is the product of glycogen hydrolysis in eukaryotic cells and there is experimental evidence that the PrfA-dependent utilization of this compound has a role in *L. monocytogenes* virulence [23],[56].

We observed an *in vivo* up regulation of several genes encoding enzymes involved in the glycolysis, like *gap*, *pgi*, *fbaA*, and *pgm*. Inversely, we found a down regulation of the expression of four genes involved in the non-oxidative phase of the pentose phosphate pathway (*lmo2660*, *lmo2661*, *lmo2662* and *lmo2674*). The final step of glycolysis leads to pyruvate, which is then converted to acetyl-CoA by the pyruvate dehydrogenase complex. We found this complex partly up regulated *in vivo*, as well as one of its activator, the lipoate ligase protein LplA2 [57],[58]. The citric acid cycle is continuously supplied with acetyl-CoA during aerobic respiration. We observed an up regulation of three citric acid cycle genes (*citBCG*) (Table S8). The citric acid cycle is followed by oxidative phosphorylation. In this study, we found the up regulation of several genes implicated in biosynthesis and assembly of components of the respiratory chain (*menD*, *lmo1677*, *qoxABD*, *ctaA*, *cydA*, *cydD*, *atpD*). In addition, genes encoding *resD*, a regulator of aerobic and anaerobic respiration [59] and *rex*, a redox-sensing transcriptional repressor [60], were also up regulated *in vivo*. Genes encoding the pyruvate-formate lyase (*pfl*) and pyruvate-formate lyase activating enzymes (*pflCB*) are required for the anaerobic metabolism of pyruvate and were activated in the host (Table S8).

Genes implicated in amino acid biosynthesis were also induced *in vivo*, in particular *aroA* and *pheA*, two genes responsible for aromatic amino acid biosynthesis. Mutations in *aroA* and *pheA* were previously shown to induce an attenuation of virulence in the mouse model [61],[62]. Furthermore, the expression of genes implicated in the biosynthetic pathways of branched amino acids (*alsS*, *ilvN* and *lmo0978*), and amino acids of the aspartate and glutamate families (*ansB*, *lmo0594*, *lmo1006*, *lmo1011*, *lmo2413* and *glnA*, *lmo2770*, respectively), was also increased *in vivo* (Table S8).

Significantly, mannose (*lmo0781–lmo0784*), maltose (*lmo0278*) and cellobiose (*lmo0301* and *lmo0915*) -specific PTS encoding genes [63] were up regulated *in vivo*. Inversely, fructose (*lmo2733*), galactitol (*lmo2665*) and mannitol (*lmo2649*) -specific PTS encoding genes appeared down regulated.

Among the genes involved in bacterial ion uptake systems, a potassium-transporting ATPase encoding gene (*kdpB*) was down regulated *in vivo*. Cobalt (*lmo1207*), manganese (*lmo1424*) and calcium (*lmo0841*) transporter systems were, inversely, up regulated. As indicated above, the ferritin and ferric uptake protein encoding genes, *fri* and *fur*, shown to be activated under low iron concentration [64],[65], appeared highly up regulated *in vivo* (Table S8).

### Detection of potential virulence genes by *in vivo* transcriptomics

A major goal of this work was the identification of genes that encode proteins that may play a role in the infectious process. To identify such virulence genes and in order to establish a short list, we arbitrarily used several criteria. The gene should be preferentially 1) highly activated during infection; 2) absent in the non pathogenic strain *L. innocua* and present in other *L. monocytogenes* strains from diverse serotypes; 3) a member of a specific protein family encoding gene (surface, secreted, stress) possibly involved in virulence; 4) controlled by virulence regulators (PrfA, VirR, CtsR, HrcA, SigB). Several candidates emerged, matching, at least, some of the above criteria (Table 6).

**Table 6:** *L. monocytogenes* EGDe genes differentially regulated in the host and potential virulence factors.

Gene designation	Gene	Annotation	Homolog in *L. innocua*	Homolog in 1/2a F6854	Homolog in 4b F2365	Homolog in 4b H7858	Fold change 24 h	Fold change 48 h	Fold change 72 h	Regulation	Secreted/Surface protein
*lmo0206*	*lmo0206*	unknown protein		LMOf6854_0214.1	LMOf2365_0217	LMOh7858_0225.1			3,11	PrfA	Secreted
*lmo0257*	*lmo0257*	unknown protein		LMOf6854_0261.2	LMOf2365_0265	LMOh7858_0288		2,12			
*lmo0540*	*lmo0540*	similar to penicillin-binding protein	lin0544	LMOf6854_0581	LMOf2365_0569	LMOh7858_0598	3,77	3,96	2,29		Secreted/Surface
*lmo0604*	*lmo0604*	similar to B subtilis YvlA protein	lin0613	LMOf6854_0642.2	LMOf2365_0633	LMOh7858_0663.2	51,45	27,43		VirR	
*lmo0788*	*lmo0788*	unknown protein	lin0781	LMOf6854_0832	LMOf2365_0804	LMOh7858_0842	60,06	31,36	20,50	PrfA	
*lmo0915*	*lmo0915*	similar to phosphotransferase system enzyme IIC		LMOf6854_0962	LMOf2365_0937	LMOh7858_0989	3,15		2,37		
*lmo1081*	*lmo1081*	similar to glucose-1-phosphate thymidyl transferase		LMOf6854_1134			8,25	5,99	5,17		
*lmo1082*	*lmo1082*	similar to dTDP-sugar epimerase		LMOf6854_1135			47,21	24,06	21,01		
*lmo1099*	*lmo1099*	similar to a protein encoded by Tn916					65,42	30,42	24,04		
*lmo1102*	*lmo1102*	similar to cadmium efflux system accessory proteins					9,71	4,51	4,72		
*lmo1290*	*lmo1290*	similar to internalin proteins (LPXTG motif)		LMOf6854_1332	LMOf2365_1307	LMOh7858_1374.2			6,24		Surface
*lmo1438*	*lmo1438*	similar to penicillin-binding protein	lin1477	LMOf6854_1481	LMOf2365_1457	LMOh7858_1533		3,55			Secreted/Surface
*lmo1521*	*lmo1521*	similar to N-acetylmuramoyl-L-alanine amidase	lin1556	LMOf6854_1568	LMOf2365_1540			2,73		Sig54	Secreted/Surface
*lmo1601*	*lmo1601*	similar to general stress protein	lin1642	LMOf6854_1653.1	LMOf2365_1622	LMOh7858_1707.			2,18	PrfA-SigB-SigL	
*lmo1602*	*lmo1602*	unknown protein	lin1643	LMOf6854_1653.2	LMOf2365_1623	LMOh7858_1707.4		4,54	2,13	PrfA-SigB-SigL	Secreted
*adeC*	*lmo1742*	highly similar to adenine deaminases	lin1853	LMOf6854_1800	LMOf2365_1767	LMOh7858_1867		2,66		VirR	
*lmo1855*	*lmo1855*	similar to similar to D-alanyl-D-alanine carboxypeptidases	lin1969	LMOf6854_1915	LMOf2365_1883	LMOh7858_1980		3,31			Surface
*lmo2048*	*lmo2048*	unknown protein	lin2154	LMOf6854_2109.1	LMOf2365_2079	LMOh7858_2176.1		4,93	2,51	SigB-HcrA	
*lmo2114*	*lmo2114*	similar to ABC transporter (ATP-binding protein)	lin2219	LMOf6854_2178	LMOf2365_2147	LMOh7858_2245		3,68	2,93	ViR-CtsR-SigL	
*lmo2115*	*lmo2115*	similar to ABC transporter (permease)	lin2220	LMOf6854_2179	LMOf2365_2148	LMOh7858_2246	4,86	5,92	3,06	ViR-SigL	
*lmo2157*	*lmo2157*	unknown protein		LMOf6854_2221	LMOf2365_2189	LMOh7858_2290.1	3,29	5,85	2,01	PrfA-SigB	
*lmo2177*	*lmo2177*	unknown protein	lin2280	LMOf6854_2241	LMOf2365_2209	LMOh7858_2310	3,36	6,14	2,23	VirR	
*fabF*	*lmo2201*	similar to 3-oxoacyl-acyl-carrier protein synthase	lin2304	LMOf6854_2265	LMOf2365_2234	LMOh7858_2335		3,90	2,03	SigB	
*lmo2203*	*lmo2203*	similar to N-acetylmuramoyl-L-alanine amidase	lin2306	LMOf6854_2266.1	LMOf2365_2236	LMOh7858_2337		4,50			Secreted/Surface
*lmo2439*	*lmo2439*	unknown protein	lin2533	LMOf6854_2499.3	LMOf2365_2411	LMOh7858_2584.4		4,44		VirR	Secreted
*gap*	*lmo2459*	glyceraldehyde-3-phosphate dehydrogenase	lin2553	LMOf6854_2520	LMOf2365_2432	LMOh7858_2608		4,49	2,37	HcrA	Surface
*lmo2522*	*lmo2522*	similar to hypothetical cell wall binding protein	lin2666	LMOf6854_2584	LMOf2365_2495	LMOh7858_2674		5,72	3,07		Secreted/Surface
*lmo2713*	*lmo2713*	secreted protein with 1 GW repeat	lin2861	LMOf6854_2831.1	LMOf2365_2693	LMOh7858_2976.1	8,86	17,08	2,80		Secreted/Surface
*lmo2714*	*lmo2714*	peptidoglycan anchored protein (LPXTG motif)	lin2862	LMOf6854_2833	LMOf2365_2694	LMOh7858_2978	25,45	70,27	3,09		Surface

*lmo0206*, *lmo0257*, *lmo0915*, *lmo1290* and *lmo2157* are genes that, as eleven already known virulence factors, are *L. monocytogenes* species-specific and induced *in vivo*. *lmo0206* and *lmo2157* are the only two genes activated *in vivo*, controlled by PrfA, absent from *L. innocua* and whose role in virulence was never investigated. *lmo0206*, *orfX* [66], is in addition located at the end of the *Listeria* virulence cluster and was recently implicated in intracellular survival [14]. The expression of *lmo2157* was shown to be controlled by PrfA and SigB [6],[8].

*lmo1081*, *lmo1082*, *lmo1099* and *lmo1102* are *L. monocytogenes* EGDe species-specific genes highly up regulated *in vivo* over the three time points of the infection (Table 6). Interestingly, these genes encode proteins potentially involved in cell wall metabolism and heavy metal detoxification.

Only two uncharacterized genes encoding LPXTG surface proteins (*lmo1290* and *lmo2714*) and three encoding GW surface proteins (*lmo1521*, *lmo2203* and *lmo2713*) were up regulated within the host (Table 6). *lmo1521* and *lmo2203* are in addition predicted autolysins. *lmo2713* and *lmo2714* seem to be part of a genomic region over expressed at all time points of the infection and Lmo2714 was found in the *Listeria* culture supernatant [43]. Four genes (*lm0540*, *lmo1438*, *lmo1855* and *lmo2522*) predicted to be involved in cell wall metabolism were up regulated *in vivo*, and similar to *pgdA*, *iap*, and *murA* [29]–[31], could participate in *Listeria* infection.

Twenty-five uncharacterized genes activated *in vivo* encode secreted proteins that may interact with the host cells, including Lmo2201, a Tat-secreted protein [42], and GAPDH. GAPDH was previously shown to be part of the *Listeria* cell wall subproteome [35], and to impair *Listeria* phagosome maturation [67]. GAPDH seems, in addition, to be implicated in the virulence of several other pathogens [68]–[70].

*lmo0788* is highly activated in mouse spleens during infection and is the only gene of the group I PrfA-regulated genes (i.e. preceded by a PrfA-box and positively regulated by PrfA) [6] whose role during infection has never been addressed (Tables 2 and 6). *lmo0788* encodes a protein similar to subunits (BadFG) of the benzoyl-CoA reductase used by facultative aerobes in absence of oxygen for reductive aromatic metabolism [71].

VirR appears as the second main regulator of virulence genes and controls *lmo0604*, *lmo1742*, *lmo2114*, *lmo2115*, *lmo2177* and *lmo2439*, whose expression was activated in the host (Tables 3 and 6). *lmo2114* and *lmo2115* are in addition part of a transcriptional unit co-regulated by CtsR and SigL [7].

Several stress protein encoding genes that are under the control of different stress regulators were up regulated *in vivo*. In particular, *lmo2048* is a stress protein-encoding gene that is co-controlled by CtsR and HrcA (Table 6). The 19 genes up regulated *in vivo* and co-controlled by PrfA and SigB (Table 2) could also be important for the infectious process. Among these, *lmo1601* and *lmo1602* are furthermore regulated by SigL [7].

The use of such arbitrary criteria obviously not guaranteed that a selected gene was a virulence factor, and conversely probably excluded many virulence genes. In particular, it is worth mentioning that 91 genes encoding proteins similar to unknown proteins, and 31 encoding putative proteins with no similarity in public databases were differentially expressed *in vivo* (Table S3), representing a large reservoir of potential new virulence factors. Of these genes, those highly regulated all over the infectious process could be of special relevance for virulence.

### Identification of new *L. monocytogenes* virulence factors

In order to validate our transcriptomics approach and identify new *L. monocytogenes* virulence factors, 6 genes (*lmo1081*, *lmo1082*, *lmo1102*, *lmo2713*, *lmo2714* and *gap*) were selected for mutagenesis using the criteria presented above. As we were unable to produce a *gap* deletion mutant (probably because GAPDH is an essential protein), we constructed a GAPDH secretion mutant.

**Figure 5:**
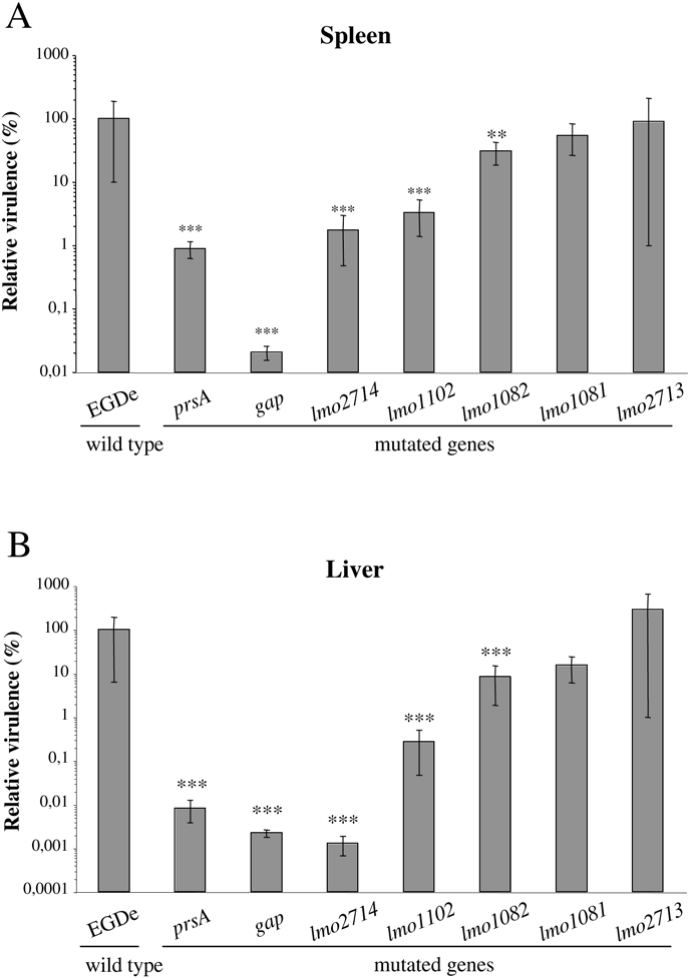
*In vivo* characterization of new *Listeria* mutants. BALB/c mice were intravenously inoculated with 10^4^ CFUs. The number of bacteria in the spleen (A) and liver (B) of mice was determined at 72 h post-infection. A *prsA* mutant was constructed and used as control. Five mice for each bacterial strain. Statistically significant differences are indicated as compared to wild type strain: ** = *P*<0.01, *** = *P*<0.001.

To analyze the potential role of the selected genes in virulence, we performed intravenous inoculations of BALB/c mice with wild type (wt) and mutant strains, and the number of bacteria in the mouse liver and spleen was determined 72 h after infection (Figure 5). Mutants can be classified with respect to their virulence potential. Bacterial counts for *lmo1081* and *lmo2713* mutants were not significantly changed as compared to the wt strain, suggesting the non-implication of these genes in *Listeria* virulence in mice. For the *lmo1082* mutant, bacterial counts were significantly affected (≈1 log) in mouse livers and at a lesser extent in the spleens. Interestingly, for *lmo1102*, *lmo2714* and *gap* mutants we observed a remarkable decrease of bacterial counts in both mouse organs as compared to the wt. In particular, the number of bacteria was dramatically impaired in the liver 72 h after inoculation (≈2,5 to 4,5 log). The *gap* mutant appeared as the most attenuated mutant of our analysis with a considerable virulence decrease in both organs reaching 3,5 log in the spleen and 4,5 log in the liver as compared to the wild type (Figure 5).

**Figure 6:**
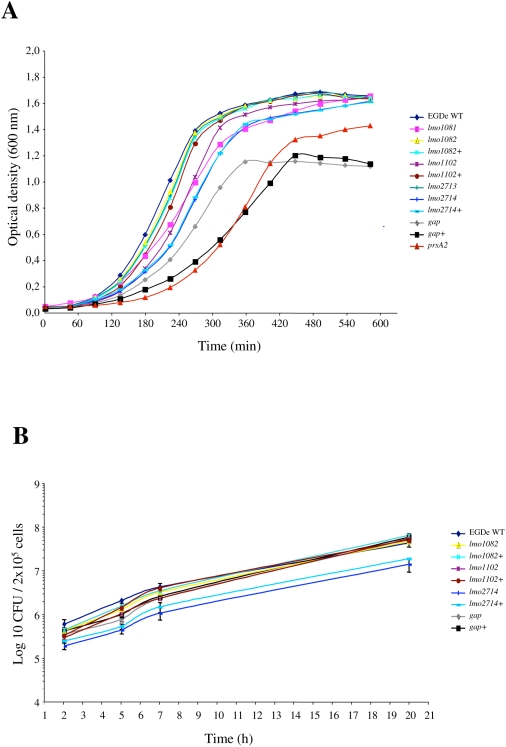
*In vitro* behavior of *L. monocytogenes* mutants. (A) Growth curves of *L. monocytogenes* EGDe strains in BHI at 37°C with shaking. (B) Intracellular behavior of *L. monocytogenes* EGDe strains in J774 cultured cells.

In order to better characterize virulence attenuated strains, mutants for *lmo1082*, *lmo1102*, *lmo2714* and *gap* were complemented. The corresponding wild-type gene was inserted as a single copy under the control of its own promoter on the chromosome of the mutant strain, at the PSA bacteriophage attachment site using the pPL2 integration vector [72]. Wild type, mutant and complemented strains were tested for growth in BHI at 37°C and for intracellular behavior after internalization in the murine macrophage cell line J774 (Figure 6).

The growth rate observed in BHI at 37°C for the majority of the strains tested was comparable to that of the wild type (Figure 6A). However, the *gap* secretion mutant exhibited an important *in vitro* reduced growth rate and reduced density at the stationary phase. The growth defect observed for the *gap* mutant was even accentuated in the complemented strain (Figure 6A). This is most probably the result of an over expression of intracellular GAPDH, expressed at the same time from the bacterial genome and from the plasmid harbored by this strain. Surprisingly, the *prsA2* mutant presented also a notable growth delay. This growth defect was not mentioned in previous studies implicating PrsA2 in intracellular behavior and virulence [14],[33].

Wild type, mutant and complemented strains were also tested for intracellular behavior. As shown in Figure 6B, all the strains grew with similar multiplication rates after internalization in J774 cells, indicating that the slight growth delay observed in BHI at 37°C for some strains has no consequences on intracellular multiplication.

**Figure 7:**
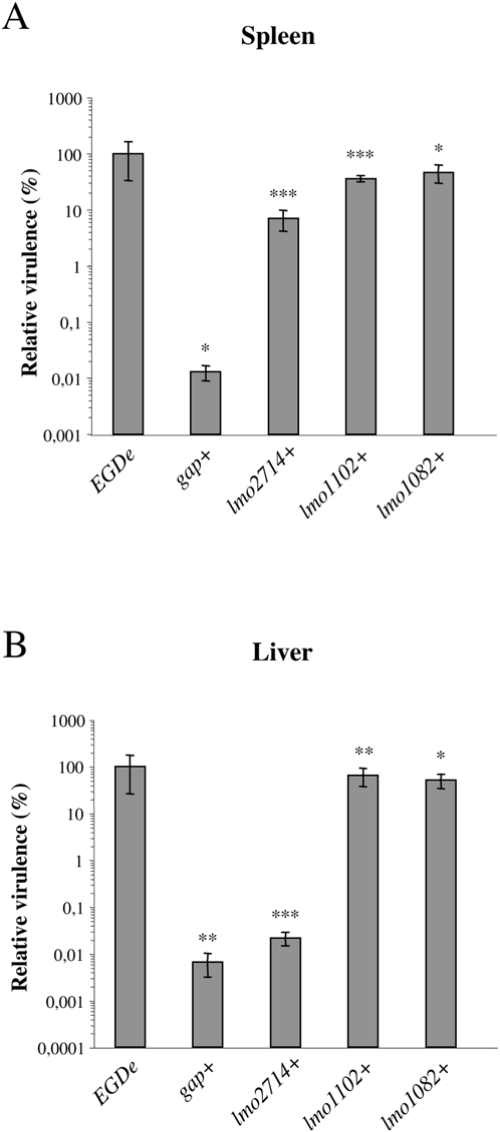
*In vivo* complementation of new *Listeria* mutants. BALB/c mice were intravenously inoculated with 10^4^ CFUs. The number of bacteria in the spleen (A) and liver (B) of mice was determined at 72 h post-infection. Five mice for each bacterial strain. Statistically significant differences are indicated as compared to the corresponding mutant for complemented strains: * = *P*<0.05, ** = *P*<0.01, *** = *P*<0.001.

In addition, complemented strains were analyzed after intravenous inoculations of BALB/c mice as compared to wt and mutant strains, and the number of bacteria in the mouse liver and spleen was determined 72 h after infection (Figure 7). The virulence phenotype was restored, albeit partially in the case of *lmo2714*, in complemented strains, except for the *gap* mutant. The virulence defect of the *gap* complemented strain was even more severe in the spleen as compare to the corresponding mutant (Figure 7A). This was in correlation with the increased growth defect observed in BHI at 37°C for the *gap* complemented strain.

These results revealed a role for *lmo1082* and *lmo1102*, and at less extent for *lmo2714 and gap* in *Listeria* virulence, validating our *in vivo* transcriptomics approach.

## Discussion

Identification of bacterial gene expression patterns during host-pathogen interactions has long been a goal for understanding infectious processes of intracellular pathogens [73]. Here, we undertook the first time course study of the *L. monocytogenes in vivo* transcriptome by comparing the genomic transcriptional patterns of bacteria grown under laboratory conditions (BHI, 37°C, exponential growth phase, pH 7) with that of *in vivo*-grown bacteria over three days of infection (mouse spleen). This constitutes also the first genome expression analysis of a pathogen in infected mouse spleens. Our results indicate that a significant part of the *Listeria* genome is differentially expressed for adaptation to the host environment, essentially through gene activation. We showed an *in vivo* over expression of an impressive number of genes involved in virulence and subversion of the host immune systems, together with genes involved in adaptation of the bacterial metabolism to host conditions and stress responses. We revealed that all these expression modifications are controlled by a complex regulatory network.

### *In vivo* activation of *Listeria* virulence mechanisms

Whereas metabolic genes represent only 22% of the *in vivo* activated genes, they constitute the major part of the down regulated genes (65%). These observations reveal that the modification of the *Listeria* genome expression during infection is dominated by the activation of virulence specific genes.

A major finding of our study was the demonstration that the majority of genes previously reported as implicated in virulence were highly up regulated in the host. We observed a peak of activation for these genes 48 h p.i., preceding the peak of bacterial loads that occurs at 72 h p.i. when mice are intravenously inoculated with a sub-lethal dose. Our results support the idea that *Listeria* uses *in vivo* a complex and coordinated regulatory network that includes virulence and stress regulators in order to tightly control genes that contribute to its survival and progression of the disease. Our study definitively establishes PrfA as the major *Listeria* virulence regulator and VirR as the second one. These two regulators, as well as a large proportion of the genes they regulate, including known virulence factors and potentially new virulence genes, were strongly activated *in vivo*. In addition, the co-control by PrfA and SigB of several genes activated *in vivo* strongly highlights the importance of the interplay between these two regulons during the infectious process. The hypothesis on an *in vivo* intersecting regulation of SigB and PrfA is in agreement with a very recent study demonstrating the contribution of SigB and PrfA to a regulatory network critical for appropriate regulation of virulence gene expression [74]. The large number of additional regulons and predicted transcriptional regulators differentially regulated inside the host underlines the high degree of regulation required for adaptation of *Listeria* to the host environment.

Another major aspect observed when *Listeria* interacts with its host is the active remodeling of the bacterial envelope through activation of the cell wall metabolism and enhanced exposure of virulence proteins at the bacterial surface.

Pathogens have evolved various systems for the secretion of bacterial factors that contribute to the progression of the disease. *Listeria* uses different secretion systems and a significant number of their products were activated *in vivo*. It is particularly the case of the SecA2 system, itself activated *in vivo*, and responsible for the secretion of several proteins lacking a signal peptide and also up regulated in the host, including known virulence factors.

Although competence genes have been found in *L. monocytogenes* genome [20], *Listeria*e have never been shown to be naturally competent. Interestingly, we observed several competence genes (*comEA*, *comEB*, *comGF*, *comGE*, *clpC*, *mecA* and *degU*) up regulated *in vivo*. This is the first report of a simultaneous activation of a great number of competence genes in *Listeria*, suggesting that this bacterium could be competent during infection and use this system to incorporate DNA from the host environment in order to acquire new potentialities.

### Subversion of host defenses

In addition to the up regulation of a number of virulence factors, *L. monocytogenes* activates mechanisms of subversion of the host defenses. Lysinylation of phospholipids in *Listeria* membranes by MprF, and D-alanylation of cell wall TAs and LTAs by the *dlt* complex lead to a reduced negative charge of the bacterial surface. One of the consequences of this process is the repulsion of cationic antimicrobial peptides [13],[25]. *In vivo* activation of both *dlt* and *mprF* by VirR strongly suggests a regulation of *L. monocytogenes* resistance to cationic peptides in the host, as previously proposed [25]. Furthermore, surface components e.g. LTAs and PG play a role in the innate immune response through receptors like Nods and Toll-like receptors [75]. LTA modification by VirR regulated factors could thus be used by *Listeria* to escape the host innate immune response.

SecA2-dependent secretion has been proposed to coordinate PG digestion by the activity of secreted autolysins [30]. The muramyl glycopeptide predicted to be generated by the combined activities of p60 and MurA is known to modify host inflammatory responses [76],[77]. Thus, the strong *in vivo* induction of SecA2 and SecA2-secreted proteins may activate release of specific peptides that interfere with host pattern recognition.

N-deacetylation by PgdA was shown to be a major modification of *Listeria* PG, conferring the ability to survive in the gastrointestinal tract, in professional phagocytes, evade the action of host lysozyme, and modulate the inflammatory response [31]. The over expression of *pgdA* during infection appears as an additional strategy used by *Listeria* to subvert host pattern recognition and control the host inflammatory responses to promote its own survival.

In the same way, in accordance to the potent proinflammatory activity of flagellin [78], *Listeria* down regulates flagella related genes (*lmo0681* and *lmo0697*) during the infection by the activation of *mogR*, a repressor of motility and chemotaxis gene expression [79].

### Adaptation of the metabolism to the host environment

Once inside the host, bacteria need to adapt to nutritional changes, including carbon and nitrogen sources. This is illustrated by the high number of metabolic genes differentially regulated *in vivo*.

UhpT and several enzymes involved in glycolysis were up regulated whereas enzymes implicated in the non-oxidative phase of the pentose phosphate pathway were repressed. These data suggest that phosphorylated glucose transported by UhpT is being metabolized through glycolysis. Glucose or phosphorylated glucose seems thus one of the major carbon sources *in vivo*, the pentose phosphate cycle appearing not essential for the generation of necessary intermediates and for gluconeogenesis. In addition to genes involved in glycolysis, we observed the *in vivo* up regulation of numerous genes implicated in the citric acid cycle and in oxidative phosphorylation, indicating that *L. monocytogenes* is using oxidative phosphorylation to generate energy. However, the activation of genes involved in fermentation, suggests that, even though *L. monocytogenes* is using oxidative phosphorylation to generate energy, it might be experiencing some level of oxygen starvation in spleen cells. The activation of the glycerol kinase and glycerol-3-phosphate dehydrogenase, indicates that glycerol, probably deriving from the activity of the phospholipases A and B on cellular lipids, is an additional carbon source for intracellular growth.

Metal ions are essential cofactors for functional expression of many proteins in bacterial systems. Thus, alterations in *Listeria* ion transport genes *in vivo* reflect the accessibility/inaccessibility of those ions in the intracellular environment, i.e presence of potassium and lack of cobalt, manganese, calcium and iron.

Due to defense mechanisms developed by the host to limit bacterial multiplication, it could be expected a growth rate decrease for invading pathogens in their host [16]. A very interesting discovery resulting from our *in vivo* transcriptomics analysis was the observation of an active growth status of *Listeria* in infected mouse spleens. This was demonstrated by the increased expression of numerous genes encoding proteins involved in bacterial growth and multiplication, including genes implicated in DNA replication and cell division. *Listeria* thus seems to have 24 h p.i. overcome organism defenses and being engaged in an active multiplication phase.

### Stress responses to the host environment

Bacterial responses to environmental changes are often characterized by the induction of specific stress responses. The *in vivo* induction of *Listeria* stress genes indicated that bacteria are faced with stress within the host. The alternative sigma factor B is the master regulator of stress. Even in the absence of a significant regulation of *sigB* itself *in vivo*, an impressive number (70) of genes previously shown to be under SigB-regulation were up regulated *in vivo*, including numerous virulence factors. In addition, genes up regulated *in vivo* and under the control of HrcA and CtsR seem also to be particularly relevant for virulence. During the process of host colonization, *L. monocytogenes* induces a host inflammatory response [80]. This defense is accompanied by the generation of ROS presented to the persistent pathogen. In addition to the traditional ROS combating enzymes like catalase and superoxide dismutase, this transcriptomic analysis revealed the *in vivo* activation of panoply of genes implicated in the response to oxidative stress, suggesting a special relevance of this response for *Listeria* pathogenesis/persistence.

### Strong difference between *in vivo* and “in cultured cells” transcriptome data

Our study highlights that the analysis of a host-pathogen interaction in its real context (i.e. the living host) is highly informative. Indeed, it is not currently feasible to reconstruct *in vitro* the exact environment faced by *Listeria* in the host. This is illustrated by the strong difference observed between our *in vivo* transcriptome and previous transcriptomes of *Listeria* growing inside epithelial cells or macrophages [14],[15], with only 15% and 29% overlap for the up regulated genes, and no more than 3% for the down regulated genes. Furthermore, 25% and 44% of the genes down regulated in epithelial cells and macrophages respectively, were up regulated during mouse infection. One of the main differences between *in vitro* intracellular growth and *in vivo* infection was the much higher number of previously known virulence genes activated *in vivo* (29 against 17), or down regulated intracellularly (8 against 2). Genes identified in this study as up regulated *in vivo* and implicated in virulence are not regulated in epithelial cells, and not regulated (*lmo1102*) or even repressed (*lmo1082*, *gap*) in macrophages. *lmo2714* is the only of these genes that appeared activated both *in vivo* and in macrophages. Other significant differences between “*in cultured cells*” and *in vivo* approaches were observed at the level of genes involved in cell division and cell wall metabolism, repressed in macrophages but activated in the host, suggesting a more active multiplication status of *Listeria* in mouse organs. *Listeria* metabolism in the two environments appeared also significantly different, in particular concerning glycolysis and the pentose phosphate pathway that, in contrast to what was observed in cultured cells [14],[15], were respectively activated and repressed in bacteria growing in mouse spleens. Finally, we observed a strong *in vivo* down regulation of flagella related genes, in accordance to the potent proinflammatory activity of flagellin [78], and inversely to what observed during intramacrophagic growth [14],[15].

### Identification of new *L. monocytogenes* virulence factors by *in vivo* genome profiling and mutagenesis

In addition to the global analysis of the expression of the entire *Listeria* genome during infection, a major goal of this study was the identification of new *Listeria* virulence factors. The *in vivo* differential expression of a remarkable number of genes previously implicated in *Listeria* intracellular survival and virulence underscores the relevance of our approach. Our analysis allowed the detection of several potential novel virulence genes. Mutagenesis of 6 of these genes demonstrated the implication of a majority of them in virulence, thus definitively establishing the value of our strategy.

*lmo2714* is a gene up regulated during infection and that encode a LPXTG surface protein [35],[36]. The probable implication of this LPXTG protein in *L. monocytogenes* virulence in mice confirms the importance of this protein family for the *Listeria*-host interaction and underlines the complexity of the mechanisms developed by this pathogen to reach a maximal infectious capacity. As other known surface virulence determinants [81],[82], Lmo2714 could interact with a specific cellular receptor or ligand that remains to be identified. Lmo2714 was also shown as present in the *Listeria* supernatant [43], and could thus also act as secreted factor.

*gap* encodes GAPDH, a glycolytic enzyme involved in bacterial energy generation that is essential for growth in the absence of neoglucogenic substrates. In *Listeria*, GADPH was previously described as a surface protein present in the cell wall, as well as a secreted protein [35],[43]. As a secreted product, GAPDH was shown to impair Rab5a mediated phagosome–endosome fusion [67]. Interestingly, GAPDH was also recently shown to be a key virulence-associated protein of *Streptococcus suis* type 2 up regulated *in vivo* [70]. The impossibility of constructing a *gap* deletion mutant confirmed the essential role of this protein in the bacterial metabolism as previously shown [70]. Using a *gap* secretion mutant, we showed that, whereas required for full growth in BHI, secreted GAPDH was not essential for intracellular multiplication. In addition, mouse infection indicated a role for secreted GAPDH in *Listeria* virulence, probably in part through its ability to retain and inactivate phagosomal Rab5a as previously described [67]. However, the *gap* secretion mutant exhibited an important *in vitro* growth defect. In addition, the complemented strain showed an accentuated growth delay *in vitro* and a more pronounced virulence decrease *in vivo*. These effects, even most probably due to an over expression of intracellular GAPDH, should be corrected in order to definitively prove the critical role of secreted GAPDH in virulence.

*lmo1082* is homolog to *rmlC* that encodes a dTDP-dehydrorhamnose epimerase potentially implicated in the surface layer (S-layer) glycoprotein synthesis [83]. *lmo1082* is furthermore part of a *L. monocytogenes* EGDe specific chromosomal region that contains two other genes that are homologous to S-layer biosynthesis genes in other organisms, several genes involved in TA biosynthesis, and the autolysin Auto previously implicated in *Listeria* virulence [84]. S-layers are two-dimensional crystalline arrays that completely cover bacterial cells. In addition to the impaired survival of the *lmo1082* mutant in mouse organs, the high *in vivo* activation of *lmo1082* could suggest a role for S-layer glycoproteins in *Listeria* virulence. S-layers have been shown to be virulence factors of several pathogens. In particular, the S-layer glycoproteins were implicated in mechanisms evolved by pathogenic bacteria to evade host immune systems. However, the examination of several strains using different techniques has so far never demonstrated the presence of S-layers in *Listeria* [85]. Preliminary experiments by electron microscopy did not allow to confirm the presence of a *Listeria* S-layer *in vivo* (data not shown). This aspect thus requires further investigation.

Lmo1102 is similar to CadC, a protein required for cadmium resistance. Cadmium is a heavy metal and its cation is toxic for microbes in the environment. Cadmium resistance in *Listeria* is an energy-dependent cadmium efflux system, involving two proteins, CadA and CadC. *Listeria cadA* and *cadC* genes for cadmium resistance were previously located, in the strains analyzed, on a transposable element (Tn5422) closely related to Tn917 and capable of intramolecular transposition [86],[87]. In the *L. monocytogenes* EGDe strain, the *cadA* and *cadC* genes are part of an EGDe specific chromosomal region located downstream an integrase encoding gene and containing 13 genes similar to Tn916 genes. This seems to indicate that *L. monocytogenes* EGDe has also acquired resistance to cadmium by transposon insertion. The strong *in vivo* activation of *cadC* and the significant impaired virulence of the *cadC* mutant suggest that this heavy metal resistance system constitutes an advantage for *in vivo Listeria* survival. The real function of CadC *in vivo* reserves further investigation.

### Concluding remarks

*In vivo*, bacteria are challenged with unique cues that are difficult to reproduce under *in vitro* conditions. The molecular analysis of *in vivo* infectious processes by means of this approach provides the first comprehensive view of how *L. monocytogenes* adapts to the host environment in the course of the infection. We showed that the remarkable shift of the *Listeria* genome expression during infection is characterized by the activation of a number of genes involved in virulence and subversion of the host immune systems, and is associated with the adaptation of the bacterial metabolism to host conditions. All these mechanisms are under the control of a complex regulatory network. As confirmed here by the identification of several new virulence genes, this analysis provides a powerful tool for the detection of novel virulence determinants and a better understanding of the complex strategies used by pathogens to promote infections.

It would be now particularly interesting to perform the same *in vivo* genome profile analysis on different infected organs (intestine, liver, brain) and using different animal models in order to identify organ- or host-specific virulence factors.

## Materials and Methods

### Bacterial strains and growth conditions

*L. monocytogenes* EGDe was grown in Brain Heart Infusion (BHI) medium (BD-Difco) or in a defined minimal medium (modified Welshimer's broth [18]) at 37°C, under aerobic conditions with shaking. Erythromycin was included at 5 µg/ml when the bacteria carried pMAD and pAUL-A derivatives. Chloramphenicol was included at 7 µg/ml when the bacteria carried pPL2 derivatives. *E. coli* strains were grown in LB medium at 37°C, with shaking. Ampicilin and erythromycin were added at 100 µg/ml and 300 µg/ml, respectively, when required.

### Isolation of *L. monocytogenes* EGDe total RNA

#### From pure culture

Cultures for preparing RNA samples were grown overnight at 37°C under aerobic conditions in liquid medium with shaking. Overnight pre-cultures were diluted in liquid medium and incubated at 37°C under aerobic conditions with shaking. Exponentially growing cells (OD_600_ = 0.6) or cells in stationary growth phase (OD_600_ = 1.5) were harvested by centrifugation for 15 min at 4700 rpm at 4°C. Total RNA was extracted as previously described [6]. Quality of RNA was assessed by determining the OD_260/280_ ratio and by visualization following agarose gel electrophoresis and ethidium bromide stain.

#### From infected mice organs

Specific pathogen-free female CD1 mice (Charles River) were intravenously infected with *L. monocytogenes* EGDe. At 24, 48 and 72 h post-infection, the mice were sacrificed and dissected. The livers and spleens were harvested and immediately frozen in liquid nitrogen. The organs were stored at −80°C. Prior to RNA isolation, the organs were thawed on ice and homogenized in 20 ml of an ice-cold solution composed of 0.2 M sucrose/0.01% SDS. The homogenate was gently centrifuged for 20 min at 300 rpm and filtered to remove large tissue debris. The tissue suspension was centrifuged for 20 min at 4000 rpm to pellet the bacteria. Centrifugations were performed at 4°C. Bacterial RNA extraction was performed as previously described [6]. Quality of RNA was assessed by determining the OD_260/280_ ratio and by visualization following agarose gel electrophoresis and ethidium bromide stain.

### Construction of whole-genome macroarrays

The macroarrays used here are described in [6]. Briefly, specific primer pairs were designed for each of the 2853 ORFs of the *L. monocytogenes* EGDe genome, in order to amplify a fragment of ∼500 bp specific for each ORF. For macroarray preparation, nylon membranes were soaked in 10 mM TE, pH 7.6. Spot blots of ORF-specific PCR products and controls were printed using a Qpix robot. Immediately after spot deposition, membranes were neutralized for 15 min in 0.5 M NaOH, 1.5 M NaCl, washed three times with distilled water and stored wet at −20°C until use.

### cDNA synthesis, labeling and hybridization to macroarrays

cDNA synthesis, labeling and hybridization were performed as previously described [6]. Briefly, cDNA was reversely transcribed in the presence of [α-^33^P]-dCTP. Labeled cDNA was purified using a QIAquick column (Qiagen). Hybridization and washing steps were carried out using SSPE buffer. Macroarrays were pre-wet in 2× SSC and pre-hybridized in hybridization solution (5× SSPE, 2% SDS, 1× Denhardt's reagent, 100 µg of sheared salmon sperm DNA/ml) at 65°C. Hybridization was carried out for 20 h at 65°C. After hybridization, membranes were washed twice at room temperature and twice at 65°C in 0.5× SSC, 0.2% SDS. For each condition, two independent RNA preparations were tested, and two cDNAs from each of the RNA preparations were hybridized to two sets of arrays and analyzed.

Array results are available at the GEO database under the accession number GPL7248 (http://www.ncbi.nlm.nih.gov/geo/query/acc.cgi?token=jvqvnqwicgkoajy&acc=GSE13057).

### Data analysis

Membranes were scanned using a 445SI PhosphoImager. The ARRAYVISION software was used for quantification of the hybridization intensities. The intensity of each spot was normalized according to the median value of the total intensities of all spots on each array. The global background was calculated from the median intensity of 610 “no-DNA” spots homogeneously distributed throughout the membrane. For spots whose intensity value was lower than the median background intensity, the intensity value was replaced by the median background intensity, for analysis purposes.

The significance analysis of microarrays (SAM) program was used for identification of genes with statistically significant changes in expression [19]. SAM was conducted with the following log_2_ ratios of gene expression values: 1) 24 h post-infection *versus* pure culture, 2) 48 h post-infection *versus* pure culture, and 3) 72 h post-infection *versus* pure culture. One-class responses were chosen to test if the mean level of gene expression differed from a hypothesized mean. A delta value corresponding to a false discovery rate <5% was chosen. Genes with at least a twofold expression change that were significant according to this analysis in at least one time point were taken into account.

For clustering analysis, data was log transformed, median centered and an average-linkage clustering was carried out using CLUSTER software and the results were visualized by TREEVIEW [88].

### Quantitative real time RT-PCR analysis

Up to 1 µg of total RNA was reverse-transcribed by using the iScript kit (Bio-Rad). Forward and reverse primers (Table S6) were designed using Primer3 software (http://frodo.wi.mit.edu/) to produce an amplicon length of 70–200 bp. A standard curve was generated for each primer pair by using four ten-fold dilutions of cDNA from *L. monocytogenes* EGDe, to ensure that PCR efficiency was 100%. Quantitative PCR was performed for 45 cycles with 2 µl of cDNA, 10 µl of 2× SYBR green PCR master mix (Bio-Rad) and 0,25 pM (each) forward and reverse primers in a final volume of 20 µl. For each primer pair, a negative control (water), was included during cDNA quantification. After PCR amplification, a melting curve was generated for every PCR product to check the specificity of the PCR reaction. Data were analyzed by the ΔΔCt method which provides the target gene expression value as unitless fold changes in the unknown sample compared with a calibrator sample [89]. Both unknown and calibrator sample target gene expression data were normalized by the relative expression of 16S rRNA.

### Mutagenesis

#### Construction of deletion mutants (*lmo1082*, *lmo1102*)

Two ∼1000 bp fragments flanking the target genes were amplified by PCR from L. monocytogenes EGDe chromosomal DNA. Primers used to generate the flanking regions are shown in Table S9 in the supplemental material (restriction sites are underlined). The purified PCR fragments were digested as stated in Table S9 and coligated in the thermosensitive plasmid pMAD [90]. Plasmid DNA of pMAD bearing the fragments was used to electroporate *L. monocytogenes* EGDe to generate the chromosomal deletion mutants, as described previously [90]. The deletions were verified by PCR analysis of chromosomal DNA using pairs of primers inside each gene (Table S9).

#### Construction of insertion mutants (*prsA2*, *lmo1081*, *lmo2713*, *lmo2714*)

A 500–1000 bp fragment internal to the target genes was amplified by PCR from *L. monocytogenes* EGDe chromosomal DNA. Primers used to generate the internal regions are shown in Table S9 (restriction sites are underlined). The purified PCR fragments were digested as stated in Table S9 and coligated in the thermosensitive plasmid pAUL-A [91]. Plasmid DNA of pAUL-A bearing the fragments was used to electroporate *L. monocytogenes* EGDe to generate the chromosomal intertion mutants, as described previously [91]. The insertions were verified by PCR analysis of chromosomal DNA using pairs of primers described in Table S9.

#### Construction of *gap* secretion mutant

The prediction of hydrophobicity of a putative hydrophobic tail was determined based on the dense alignment surface (DAS) score as described at the website http://www.sbc.su.se/~miklos/DAS/. The amino acid sequence of a hydrophobic tail to be inserted at the C-terminal end of GAPDH was based on the translated amino acid sequence of the *actA* gene and was edited using the DAS method to get an optimum DAS score (≥3.0), one indicative of a typical transmembrane location [92]. The *gap* gene and a ∼1000 bp DNA fragment corresponding to the downstream region of the *gap* gene was amplified by PCR using primer pairs described in Table S9 (restriction sites are underlined, hydrophobic tail is in boldface). The purified PCR products were cloned in the multiple cloning sites located upstream and downstream of the kanamycin resistance gene in the plasmid pOD23 [93]. pOD23 bearing the fragments was used to electroporate *L. monocytogenes* EGDe to generate the chromosomal secretion mutant, as described previously [93].

### Complementation

For complementation, the entire gene and flanking regions were amplified using primers described in Table S9. PCR products were digested as described in Table S9 and ligated to the site-specific phage integration vector pPL2 [72]. Plasmid DNA of pPL2 bearing the fragments was transformed into *E. coli* S17-1 and the resulting strain was mated into each mutant strain. Chloramphenicol-resistant transconjugants were tested by PCR for pPL2 integration at the appropriate chromosomal site using primers PL102 (5′-TATCAGACCAACCCAAACCTTCC-3′) and PL95 (5′-ACATAATCAGTCCAAAGTAGATGC-3′). Primers described in Table S9 were used to confirm the presence of each gene in the respective complemented strain.

### Virulence studies

Animal experiments were performed as previously described in [94]. Bacterial growth in mice was studied by injecting 6-week-old specific pathogen-free female BALB/c mice (Charles River) intravenously with a sublethal bacterial inoculum, 10^4^ CFUs, of wild type or mutant strains. At 72 h after infection the liver and spleen were sterilely dissected and the number of CFUs was determined by plating serial dilutions of organ (liver and spleen) homogenates on BHI agar medium (five animals for each strain).

### Ethics statement

All animals were handled in strict accordance with good animal practice as defined by the relevant national and local animal welfare bodies, and all animal work was approved by the Direcção Geral de Veterinária (FCT-POCI/SAU-MMO/60443/2004, FCT-PTDC/SAU-MII/65406/2006).

### Intracellular multiplication assay

*Listeria* strains were grown to OD_600_ = 0.6, washed and diluted in DMEM such that the MOI was about 10 bacteria per cell. Bacterial suspensions were added to J774A.1 cells for 45 min. Cells were then washed and non-phagocytosed bacteria were killed by adding 20 µg/ml gentamicin for 1 h15 min. After washing, cells were lysed in 0.2% Triton X-100, at 2 h, 5 h, 7 h and 20 h post-infection and the number of viable bacteria released from the cells was assessed after serial dilutions of the lysates on BHI agar plates. Experiments were repeated two times in triplicate.

## Supporting Information

Figure S1 Growth and pH curves of *L. monocytogenes* EGDe in BHI at 37°C with shaking
(0.07 MB PDF)

Figure S2 Validation of macroarray data by real-time RT-PCR. Fold changes in *in vivo* gene expression 24 h p.i. (A) or 72 h p.I. (B) compared to that in BHI were measured by macroarray and real-time RT-PCR, log transformed and compared for correlation analysis.
(0.04 MB PDF)

Table S1 Known *L. monocytogenes* virulence factors
(0.03 MB PDF)

Table S2 *L. monocytogenes* genes regulated in the host as compared to exponential or stationary growth phase in BHI
(0.05 MB PDF)

Table S3 *L. monocytogenes* genes differentially regulated in the host as compared to exponential growth in BHI at 37°C
(0.07 MB PDF)

Table S4 *L. monocytogenes* EGDe genes encoding proteins of the cell wall subproteome and differentially regulated in the host
(0.03 MB PDF)

Table S5 *L. monocytogenes* genes encoding secreted proteins and differentially regulated in the host
(0.03 MB PDF)

Table S6 *L. monocytogenes* genes involved in DNA metabolism, RNA and protein synthesis, cell division and multiplication, and up regulated in the host
(0.03 MB PDF)

Table S7 *L. monocytogenes* genes involved in stress responses and differentially regulated in the host
(0.04 MB PDF)

Table S8 *L. monocytogenes* genes involved in metabolism and differentially regulated in the host
(0.05 MB PDF)

Table S9 Primers
(0.04 MB PDF)
