# Supplementary material for: In Vivo Transcriptional Profiling of Listeria monocytogenes and Mutagenesis Identify New Virulence Factors Involved in Infection
Source: PLoS Pathog. 2009 May 29;5(5):e1000449. doi: 10.1371/journal.ppat.1000449 (PMC2679221; doi:10.1371/journal.ppat.1000449)
Supplement: Figure S1 — Growth and pH curves of L. monocytogenes EGDe in BHI at 37°C with shaking (0.07 MB PDF) [file ppat.1000449.s001.pdf]

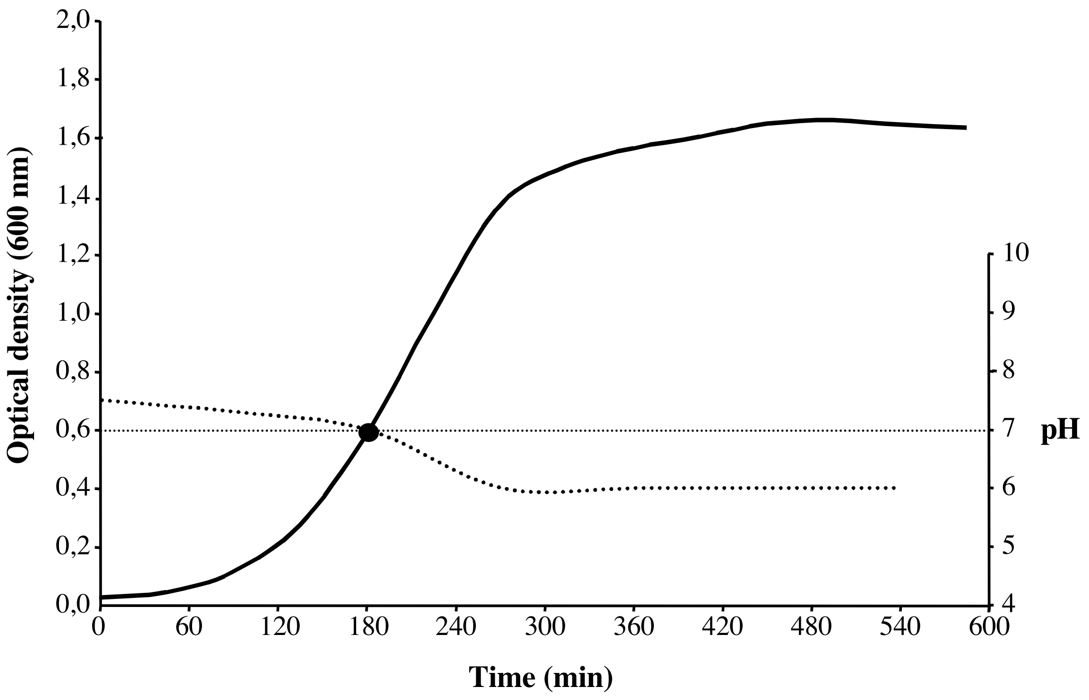

Figure S1: Growth and pH curves of *L. monocytogenes* EGDe in BHI at 37°C with shaking

Growth curve

pH curve of the medium during bacterial growth

Growth and pH conditions used to prepare *in vitro* RNAs
