# Supplementary material for: In Vivo Transcriptional Profiling of Listeria monocytogenes and Mutagenesis Identify New Virulence Factors Involved in Infection
Source: PLoS Pathog. 2009 May 29;5(5):e1000449. doi: 10.1371/journal.ppat.1000449 (PMC2679221; doi:10.1371/journal.ppat.1000449)
Supplement: Figure S2 — Validation of macroarray data by real-time RT-PCR. Fold changes in in vivo gene expression 24 h p.i. (A) or 72 h p.I. (B) compared to that in BHI were measured by macroarray and real-time RT-PCR, log transformed and compared for correlation analysis. (0.04 MB PDF) [file ppat.1000449.s002.pdf]

A

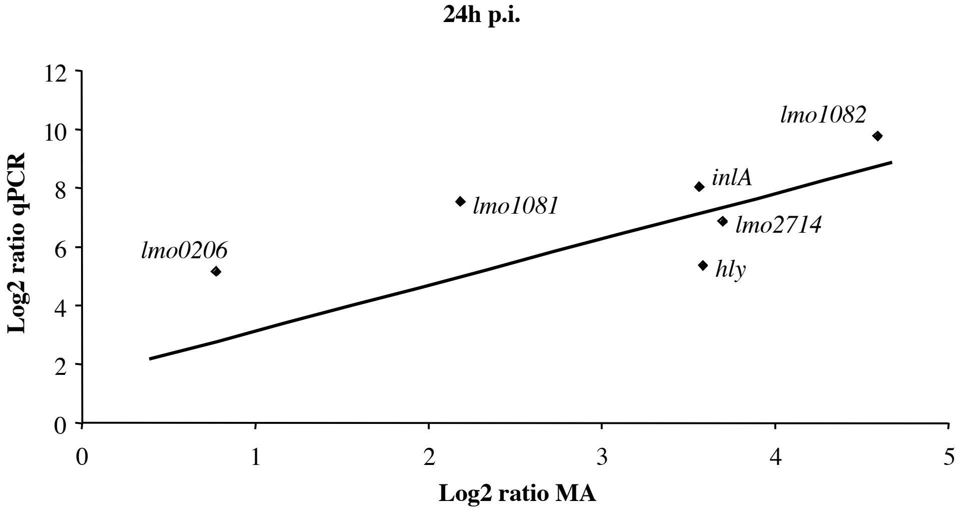

B

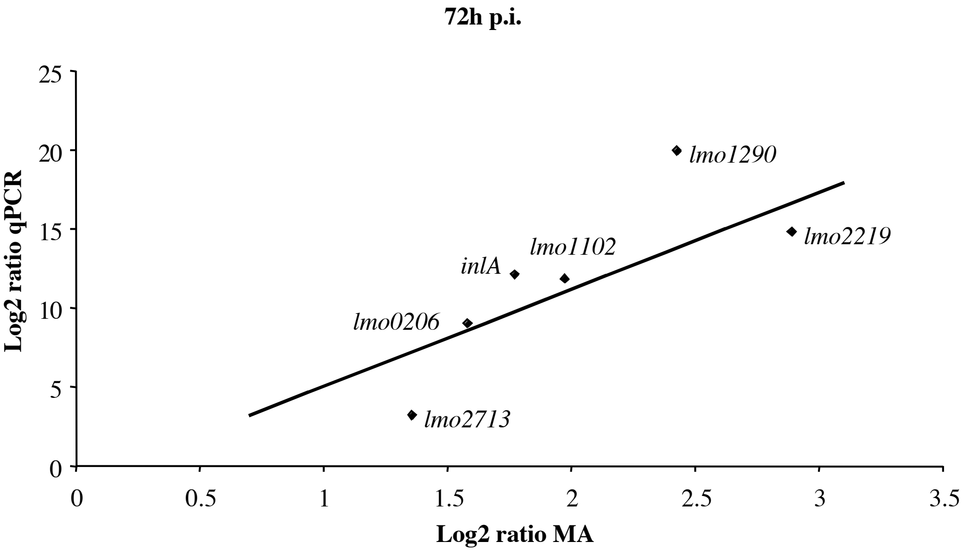

Figure S2: Validation of macroarray data by real-time RT-PCR. Fold changes in *in vivo* gene expression 24h p.i. (A) or 72h p.I. (B) compared to that in BHI were measured by macroarray and real-time RT-PCR, log transformed and compared for correlation analysis.
