# Supplementary material for: In Vivo Transcriptional Profiling of Listeria monocytogenes and Mutagenesis Identify New Virulence Factors Involved in Infection
Source: PLoS Pathog. 2009 May 29;5(5):e1000449. doi: 10.1371/journal.ppat.1000449 (PMC2679221; doi:10.1371/journal.ppat.1000449)
Supplement: Table S1 — Known L. monocytogenes virulence factors (0.03 MB PDF) [file ppat.1000449.s003.pdf]

**Table S1**. *Known L. monocytogenes virulence factors*

| Gene designation | Gene | Reference |
|---|---|---|
| ctsR | lmo0229 | Karatzas KA, Wouters JA, Gahan CG, Hill C, Abee T, Bennik MH. Mol Microbiol. 2003;49(5):1227-38. |
| lmo0135 | lmo0135 | Port GC, Freitag NE. Infect Immun. 2007;75(12):5886-97. |
| agrA | lmo0051 | Autret N, Raynaud C, Dubail I, Berche P, Charbit A. Infect Immun. 2003;71(8):4463-71. |
| actA | lmo0204 | Kocks C, Gouin E, Tabouret M, Berche P, Ohayon H, Cossart P. 1992. Cell 68:521–531. Domann E, Wehland J, Rohde M, Pistor S, Hartl M, Goebel W, Leimeister-Wächter M, Wuenscher M, Chakraborty T. EMBO J. 1992; 11(5):1981-90. |
| ami | lmo2558 | Milohanic E, Jonquières R, Cossart P, Berche P, Gaillard JL. Mol Microbiol. 2001;39(5):1212-24. |
| aut | lmo1076 | Cabanes D, Dussurget O, Dehoux P, Cossart P. Mol Microbiol. 2004;51(6):1601-14. |
| bilE | lmo1421 | Sleator RD, Wemekamp-Kamphuis HH, Gahan CG, Abee T, Hill C. Mol Microbiol. 2005;55(4):1183-95. |
| bsh | lmo2067 | Dussurget O, Cabanes D, Dehoux P, Lecuit M, Buchrieser C, Glaser P, Cossart P. Mol Microbiol. 2002;45(4):1095-106. |
| clpB | lmo2206 | Chastanet A, Derre I, Nair S, Msadek T. J Bacteriol. 2004;186(4):1165-74. |
| degU | lmo2515 | Knudsen GM, Olsen JE, Dons L. FEMS Microbiol Lett. 2004;240(2):171-9. |
| dltA | lmo0974 | Abachin E, Poyart C, Pellegrini E, Milohanic E, Fiedler F, Berche P, Trieu-Cuot P. Mol Microbiol. 2002;43(1):1-14. |
| fbpA | lmo1829 | Dramsi S, Bourdichon F, Cabanes D, Lecuit M, Fsihi H, Cossart P. Mol Microbiol. 2004;53(2):639-49. |
| fri | lmo0943 | Dussurget O, Dumas E, Archambaud C, Chafsey I, Chambon C, Hébraud M, Cossart P. FEMS Microbiol Lett. 2005;250(2):253-61 |
| fur | lmo1956 | Rea RB, Gahan CG, Hill C. Infect Immun. 2004;72(2):717-27 |
| hfq | lmo1295 | Christiansen JK, Larsen MH, Ingmer H, Søgaard-Andersen L, Kallipolitis BH. J Bacteriol. 2004;186(11):3355-62. |
| hly | lmo0202 | Gaillard JL, Berche P, Sansonetti P. Infect Immun. 1986;52(1):50-5. |
| htrA | lmo0292 | Stack HM, Sleator RD, Bowers M, Hill C, Gahan CG. Appl Environ Microbiol. 2005;71(8):4241-7. |
| iap | lmo0582 | Pilgrim S, Kolb-Maurer A, Gentschev I, Goebel W, Kuhn M. 2003; Infect. Immun. 71:3473-3484. |
| inlA | lmo0433 | Gaillard JL, Berche P, Frehel C, Gouin E, Cossart P. Cell. 1991;65(7):1127-41. Lecuit M, Vandormael-Pournin S, Lefort J, Huerre M, Gounon P, Dupuy C, Babinet C, Cossart P. Science. 2001;292(5522):1722-5. |
| inlB | lmo0434 | Dramsi S, Bourdichon F, Cabanes D, Lecuit M, Fsihi H, Cossart P. Mol Microbiol. 2004;53(2):639-49. |
| inlC | lmo1786 | Engelbrecht F, Chun SK, Ochs C, Hess J, Lottspeich F, Goebel W, Sokolovic Z. Mol Microbiol. 1996;21(4):823-37. |
| inlH | lmo0263 | Schubert WD, Göbel G, Diepholz M, Darji A, Kloer D, Hain T, Chakraborty T, Wehland J, Domann E, Heinz DW. J Mol Biol. 2001;312(4):783-94. |
| inlJ | lmo2821 | Sabet C, Lecuit M, Cabanes D, Cossart P, Bierne H. Infect Immun. 2005;73(10):6912-22. |
| lmo2026 | lmo2026 | Autret N, Dubail I, Trieu-Cuot P, Berche P, Charbit A. Infect Immun. 2001;69(4):2054-65. |
| lplA1 | lmo0931 | O'Riordan M, Moors MA, Portnoy DA. Science. 2003;302(5644):462-4. |
| mogR | lmo0674 | Gründling A, Burrack LS, Bouwer HG, Higgins DE. Proc Natl Acad Sci U S A. 2004;101(33):12318-23. |
| mpl | lmo0203 | Raveneau J, Geoffroy C, Beretti JL, Gaillard JL, Alouf JE, Berche P. Infect Immun. 1992;60(3):916-21. Vazquez-Boland JA, Kocks C, Dramsi S, Ohayon H, Geoffroy C, Mengaud J, Cossart P. Infect Immun. 1992;60(1):219-30. |
| mprF | lmo1695 | Thedieck K, Hain T, Mohamed W, Tindall BJ, Nimtz M, Chakraborty T, Wehland J, Jänsch L. Mol Microbiol. 2006;62(5):1325-39. |
| murA | lmo2691 | Lenz LL, Mohammadi S, Geissler A, Portnoy DA. Proc Natl Acad Sci U S A. 2003;100(21):12432-7. |
| oppA | lmo2196 | Borezee E, Pellegrini E, Berche P. Infect Immun. 2000;68(12):7069-77. |
| perR | lmo1683 | Rea RB, Gahan CG, Hill C. Infect Immun. 2004;72(2):717-27 |
| pgdA | lmo0415 | Boneca IG, Dussurget O, Cabanes D, Nahori MA, Sousa S, Lecuit M, Psylinakis E, Bouriotis V, Hugot JP, Giovannini M, Coyle A, Bertin J, Namane A, Rousselle JC, Cayet N, Prévost MC, Balloy V, Chignard M, Philpott DJ, Cossart P, Girardin SE. Proc Natl Acad Sci U S A. 2007;104(3):997-1002. |
| plcA | lmo0201 | Mengaud J, Braun-Breton C, Cossart P. Mol Microbiol. 1991;5(2):367-72. Camilli A, Goldfine H, Portnoy DA. 1991. J. Exp. Med. 173:751–754. |
| plcB | lmo0205 | Vazquez-Boland JA, Kocks C, Dramsi S, Ohayon H, Geoffroy C, Mengaud J, Cossart P. 1992. Infect. Immun. 60:219–230. |
| prfA | lmo0200 | Chakraborty T, Leimeister-Wächter M, Domann E, Hartl M, Goebel W, Nichterlein T, Notermans S. J Bacteriol. 1992;174(2):568-74. |
| prsA2 | lmo2219 | Port GC, Freitag NE. Infect Immun. 2007;75(12):5886-97. |
| relA | lmo1523 | Taylor CM, Beresford M, Epton HA, Sigee DC, Shama G, Andrew PW, Roberts IS. J Bacteriol. 2002;184(3):621-8. |
| secA2 | lmo0583 | Lenz LL, Mohammadi S, Geissler A, Portnoy DA. Proc Natl Acad Sci U S A. 2003;100(21):12432-7. |
| sigB | lmo0895 | Garner MR, Njaa BL, Wiedmann M, Boor KJ. Infect Immun. 2006;74(2):876-86. |
| sipX | lmo1269 | Bonnemain C, Raynaud C, Réglier-Poupet H, Dubail I, Frehel C, Lety MA, Berche P, Charbit A. Mol Microbiol. 2004;51(5):1251-66. |
| sipZ | lmo1271 | Bonnemain C, Raynaud C, Réglier-Poupet H, Dubail I, Frehel C, Lety MA, Berche P, Charbit A. Mol Microbiol. 2004;51(5):1251-66. |
| sod | lmo1439 | Archambaud C, Nahori MA, Pizarro-Cerda J, Cossart P, Dussurget O. J Biol Chem. 2006;281(42):31812-22. |
| srtA | lmo0929 | Bierne H, Mazmanian SK, Trost M, Pucciarelli MG, Liu G, Dehoux P, Jänsch L, Garcia-del Portillo F, Schneewind O, Cossart P. Mol Microbiol. 2002;43(4):869-81. |
| stp | lmo1821 | Archambaud C, Gouin E, Pizarro-Cerda J, Cossart P, Dussurget O. Mol Microbiol. 2005;56(2):383-96. |
| svpA | lmo2185 | Borezée E, Pellegrini E, Beretti JL, Berche P. Microbiology. 2001;147(Pt 11):2913-23. |
| tcsA | lmo1388 | Port GC, Freitag NE. Infect Immun. 2007;75(12):5886-97. |
| tig | lmo1267 | Bigot A, Botton E, Dubail I, Charbit A. Appl Environ Microbiol. 2006;72(10):6623-31. |
| uhpT | lmo0838 | Chico-Calero I, Suárez M, González-Zorn B, Scortti M, Slaghuis J, Goebel W, Vázquez-Boland JA. Proc Natl Acad Sci U S A. 2002;99(1):431-6. |
| vip | lmo0320 | Cabanes D, Sousa S, Cebriá A, Lecuit M, García-del Portillo F, Cossart P. EMBO J. 2005;24(15):2827-38. |
| virR | lmo1745 | Mandin P, Fsihi H, Dussurget O, Vergassola M, Milohanic E, Toledo-Arana A, Lasa I, Johansson J, Cossart P. Mol Microbiol. 2005;57(5):1367-80. |

Red: Genes up-regulated *in vivo*
Green: Genes down-regulated *in vivo*
Black: Genes not differentially regulated *in vivo*
