# Supplementary material for: In Vivo Transcriptional Profiling of Listeria monocytogenes and Mutagenesis Identify New Virulence Factors Involved in Infection
Source: PLoS Pathog. 2009 May 29;5(5):e1000449. doi: 10.1371/journal.ppat.1000449 (PMC2679221; doi:10.1371/journal.ppat.1000449)
Supplement: Table S2 — L. monocytogenes genes regulated in the host as compared to exponential or stationary growth phase in BHI (0.05 MB PDF) [file ppat.1000449.s004.pdf]

Table S2. *L. monocytogenes* EGDe genes regulated in the host, as compared to exponential or stationary growth phase in BHI

**Genes up regulated in the host, as compared to:**				**Genes down regulated in the host, as compared to:**			
Exponential growth phase in BHI		Stationary growth phase in BHI		Exponential growth phase in BHI		Stationary growth phase in BHI	
Gene designation	Gene	Gene designation	Gene	Gene designation	Gene	Gene designation	Gene
dnaA	lmo0001	dnaN	lmo0002	lmo0023	lmo0023	lmo0008	lmo0008
RecF	lmo0005	RecF	lmo0005	lmo0024	lmo0024	lmo0020	lmo0020
gyrA	lmo0007	gyrA	lmo0007	lmo0032	lmo0032	lmo0034	lmo0034
lmo0010	lmo0010	lmo0010	lmo0010	lmo0033	lmo0033	rpsF	lmo0044
lmo0011	lmo0011	lmo0011	lmo0011	lmo0391	lmo0391	lmo0078	lmo0078
lmo0012	lmo0012	lmo0012	lmo0012	lmo0393	lmo0393	lmo0080	lmo0080
qoxA	lmo0013	qoxA	lmo0013	lmo0496	lmo0496	lmo0081	lmo0081
qoxB	lmo0014	qoxB	lmo0014	lmo0681	lmo0681	lmo0087	lmo0087
qoxD	lmo0016	qoxC	lmo0015	lmo0697	lmo0697	lmo0088	lmo0088
ssb	lmo0045	lmo0019	lmo0019	lmo1142	lmo1142	lmo0093	lmo0093
rpsR	lmo0046	lmo0025	lmo0025	lmo1143	lmo1143	lmo0094	lmo0094
rplI	lmo0053	lmo0031	lmo0031	lmo1144	lmo1144	lmo0102	lmo0102
dnaC	lmo0054	lmo0042	lmo0042	lmo1145	lmo1145	lmo0105	lmo0105
purA	lmo0055	spl	lmo0045	lmo1146	lmo1146	lmo0113	lmo0113
lmo0098	lmo0098	rpsR	lmo0046	lmo1147	lmo1147	lmaC	lmo0116
lmo0150	lmo0150	lmo0050	lmo0050	lmo1148	lmo1148	lmo0128	lmo0128
lmo0153	lmo0153	lmo0052	lmo0052	lmo1149	lmo1149	lmo0130	lmo0130
lmo0158	lmo0158	rplI	lmo0053	lmo1150	lmo1150	lmo0134	lmo0134
lmo0161	lmo0161	purA	lmo0055	lmo1151	lmo1151	lmo0136	lmo0136
lmo0162	lmo0162	lmo0063	lmo0063	lmo1152	lmo1152	lmo0140	lmo0140
lmo0165	lmo0165	lmo0065	lmo0065	lmo1153	lmo1153	lmo0142	lmo0142
lmo0169	lmo0169	lmo0067	lmo0067	lmo1154	lmo1154	lmo0143	lmo0143
lmo0189	lmo0189	lmo0098	lmo0098	lmo1156	lmo1156	lmo0144	lmo0144
lmo0190	lmo0190	lmo0109	lmo0109	lmo1157	lmo1157	lmo0146	lmo0146
lmo0196	lmo0196	lmo0119	lmo0119	lmo1158	lmo1158	lmo0154	lmo0154
prfA	lmo0200	lmo0125	lmo0125	lmo1159	lmo1159	lmo0155	lmo0155
plcA	lmo0201	lmo0137	lmo0137	lmo1160	lmo1160	lmo0156	lmo0156
hly	lmo0202	lmo0150	lmo0150	lmo1161	lmo1161	lmo0157	lmo0157
mpl	lmo0203	lmo0158	lmo0158	lmo1162	lmo1162	lmo0161	lmo0161
actA	lmo0204	lmo0162	lmo0162	lmo1164	lmo1164	lmo0163	lmo0163
plcB	lmo0205	lmo0169	lmo0169	lmo1165	lmo1165	lmo0166	lmo0166
lmo0206	lmo0206	lmo0178	lmo0178	lmo1166	lmo1166	lmo0171	lmo0171
lmo0207	lmo0207	lmo0190	lmo0190	glpF	lmo1167	lmo0174	lmo0174
lmo0208	lmo0208	lmo0196	lmo0196	AckA2	lmo1168	lmo0175	lmo0175
ctc	lmo0211	prfA	lmo0200	cobD	lmo1169	lmo0179	lmo0179
lmo0216	lmo0216	plcA	lmo0201	lmo1170	lmo1170	lmo0184	lmo0184
ftsH	lmo0220	hly	lmo0202	pduQ	lmo1171	lmo0185	lmo0185
lmo0222	lmo0222	mpl	lmo0203	lmo1172	lmo1172	lmo0186	lmo0186
sul	lmo0224	actA	lmo0204	lmo1173	lmo1173	prs	lmo0199
folA	lmo0225	plcB	lmo0205	eutA	lmo1174	lmo0212	lmo0212
folK	lmo0226	lmo0208	lmo0208	eutB	lmo1175	lmo0215	lmo0215
lmo0227	lmo0227	ctc	lmo0211	eutC	lmo1176	lmo0219	lmo0219
lysS	lmo0228	lmo0216	lmo0216	lmo1177	lmo1177	lmo0234	lmo0234
lmo0230	lmo0230	lmo0218	lmo0218	lmo1180	lmo1180	rpll	lmo0251
lmo0231	lmo0231	ftsH	lmo0220	lmo1181	lmo1181	lmo0252	lmo0252
clpC	lmo0232	ssb	lmo0224	lmo1182	lmo1182	rpoB	lmo0258
lmo0236	lmo0236	lmo0227	lmo0227	cbiA	lmo1191	lmo0270	lmo0270
gltX	lmo0237	lysS	lmo0228	lmo1192	lmo1192	lmo0274	lmo0274
secE	lmo0245	lmo0231	lmo0231	cbiD	lmo1194	lmo0277	lmo0277
nusG	lmo0246	clpC	lmo0232	lmo1728	lmo1728	lmo0280	lmo0280
rplK	lmo0248	lmo0236	lmo0236	lmo1812	lmo1812	lmo0281	lmo0281
rplA	lmo0249	gltX	lmo0237	lmo1817	lmo1817	lmo0284	lmo0284
rplJ	lmo0250	cysE	lmo0238	lmo1818	lmo1818	lmo0285	lmo0285
rpll	lmo0251	lmo0242	lmo0242	lmo1819	lmo1819	lmo0286	lmo0286
lmo0257	lmo0257	nusG	lmo0246	lmo1821	lmo1821	lmo0288	lmo0288
rpoC	lmo0259	rplK	lmo0248	lmo1822	lmo1822	lmo0293	lmo0293
inlH	lmo0263	rplA	lmo0249	fmt	lmo1823	lmo0296	lmo0296
lmo0265	lmo0265	rplJ	lmo0250	priA	lmo1824	lmo0300	lmo0300
lmo0278	lmo0278	lmo0257	lmo0257	lmo1825	lmo1825	lmo0301	lmo0301
lmo0279	lmo0279	rpoC	lmo0259	lmo1828	lmo1828	lmo0302	lmo0302
lmo0280	lmo0280	inlH	lmo0263	lmo1829	lmo1829	lmo0304	lmo0304
lmo0301	lmo0301	lmo0265	lmo0265	lmo1830	lmo1830	lmo0305	lmo0305
lmo0303	lmo0303	lmo0273	lmo0273	pyrE	lmo1831	lmo0310	lmo0310
lmo0355	lmo0355	lmo0278	lmo0278	pyrF	lmo1832	lmo0315	lmo0315
lmo0366	lmo0366	lmo0279	lmo0279	pyrD	lmo1833	lmo0316	lmo0316
lmo0368	lmo0368	lmo0311	lmo0311	pyrDII	lmo1834	lmo0319	lmo0319
lmo0369	lmo0369	lmo0355	lmo0355	pyrAB	lmo1835	lmo0334	lmo0334
lmo0370	lmo0370	lmo0365	lmo0365	pyrAa	lmo1836	lmo0338	lmo0338
lmo0408	lmo0408	lmo0366	lmo0366	pyrC	lmo1837	lmo0340	lmo0340
lmo0411	lmo0411	lmo0368	lmo0368	pyrP	lmo1839	lmo0374	lmo0374
lmo0415	lmo0415	lmo0369	lmo0369	lsp	lmo1844	lmo0386	lmo0386
lmo0422	lmo0422	lmo0370	lmo0370	lmo1845	lmo1845	lmo0419	lmo0419
inlA	lmo0433	lmo0380	lmo0380	lmo1851	lmo1851	lmo0426	lmo0426
inlB	lmo0434	lmo0384	lmo0384	lmo1869	lmo1869	lmo0432	lmo0432
lmo0439	lmo0439	lmo0391	lmo0391	lmo1871	lmo1871	lmo0445	lmo0445
lmo0443	lmo0443	lmo0393	lmo0393	lmo1872	lmo1872	lmo0446	lmo0446
lmo0452	lmo0452	lmo0410	lmo0410	lmo1883	lmo1883	lmo0447	lmo0447
lmo0471	lmo0471	lmo0415	lmo0415	lmo2175	lmo2175	lmo0448	lmo0448
lmo0481	lmo0481	lmo0420	lmo0420	lmo2257	lmo2257	lmo0449	lmo0449
lmo0483	lmo0483	lmo0422	lmo0422	lmo2276	lmo2276	lmo0454	lmo0454
lmo0484	lmo0484	lmo0428	lmo0428	lmo2425	lmo2425	lmo0455	lmo0455
rpmF	lmo0486	inlA	lmo0433	lmo2432	lmo2432	lmo0456	lmo0456
prs	lmo0509	inlB	lmo0434	lmo2437	lmo2437	lmo0457	lmo0457
lmo0512	lmo0512	lmo0439	lmo0439	lmo2454	lmo2454	lmo0458	lmo0458
lmo0516	lmo0516	lmo0481	lmo0481	lmo2574	lmo2574	lmo0459	lmo0459
lmo0539	lmo0539	lmo0483	lmo0483	lmo2583	lmo2583	lmo0460	lmo0460
lmo0540	lmo0540	rpmF	lmo0486	lmo2586	lmo2586	lmo0462	lmo0462
lmo0541	lmo0541	lmo0494	lmo0494	lmo2642	lmo2642	lmo0463	lmo0463
lmo0558	lmo0558	lmo0505	lmo0505	lmo2644	lmo2644	lmo0464	lmo0464
lmo0580	lmo0580	prs	lmo0509	lmo2645	lmo2645	lmo0465	lmo0465
iap	lmo0582	lmo0512	lmo0512	lmo2646	lmo2646	lmo0467	lmo0467
lmo0583	lmo0583	lmo0516	lmo0516	lmo2648	lmo2648	lmo0468	lmo0468
lmo0584	lmo0584	lmo0525	lmo0525	lmo2649	lmo2649	lmo0474	lmo0474
lmo0590	lmo0590	lmo0530	lmo0530	lmo2657	lmo2657	lmo0479	lmo0479
lmo0592	lmo0592	lmo0539	lmo0539	lmo2660	lmo2660	lmo0480	lmo0480

lmo0593	lmo0593	lmo0541	lmo0541	lmo2661	lmo2661	rpmF	lmo0486
lmo0594	lmo0594	lmo0542	lmo0542	lmo2662	lmo2662	lmo0487	lmo0487
lmo0596	lmo0596	lmo0553	lmo0553	lmo2663	lmo2663	lmo0488	lmo0488
lmo0597	lmo0597	lmo0557	lmo0557	lmo2664	lmo2664	lmo0490	lmo0490
lmo0599	lmo0599	lmo0558	lmo0558	lmo2665	lmo2665	lmo0497	lmo0497
lmo0600	lmo0600	lmo0559	lmo0559	lmo2669	lmo2669	lmo0498	lmo0498
lmo0601	lmo0601	lmo0560	lmo0560	lmo2672	lmo2672	lmo0499	lmo0499
lmo0604	lmo0604	hisH	lmo0565	lmo2674	lmo2674	lmo0500	lmo0500
lmo0606	lmo0606	hisD	lmo0567	lmo2676	lmo2676	lmo0501	lmo0501
lmo0607	lmo0607	lmo0577	lmo0577	kdpB	lmo2681	lmo0503	lmo0503
lmo0608	lmo0608	lmo0581	lmo0581	lmo2733	lmo2733	lmo0504	lmo0504
lmo0609	lmo0609	iap	lmo0582	lmo2736	lmo2736	lmo0506	lmo0506
lmo0611	lmo0611	lmo0583	lmo0583	lmo2741	lmo2741	lmo0507	lmo0507
lmo0612	lmo0612	lmo0584	lmo0584	lmo2759	lmo2759	lmo0508	lmo0508
lmo0618	lmo0618	lmo0589	lmo0589	lmo2761	lmo2761	lmo0510	lmo0510
lmo0619	lmo0619	lmo0590	lmo0590	lmo2763	lmo2763	lmo0515	lmo0515
lmo0620	lmo0620	lmo0592	lmo0592			lmo0517	lmo0517
lmo0621	lmo0621	lmo0593	lmo0593			lmo0518	lmo0518
lmo0623	lmo0623	lmo0594	lmo0594			lmo0520	lmo0520
lmo0624	lmo0624	lmo0595	lmo0595			lmo0521	lmo0521
lmo0625	lmo0625	lmo0596	lmo0596			lmo0522	lmo0522
lmo0628	lmo0628	lmo0597	lmo0597			lmo0523	lmo0523
lmo0629	lmo0629	lmo0599	lmo0599			lmo0524	lmo0524
lmo0637	lmo0637	lmo0601	lmo0601			lmo0526	lmo0526
lmo0639	lmo0639	lmo0604	lmo0604			lmo0528	lmo0528
lmo0644	lmo0644	lmo0605	lmo0605			lmo0529	lmo0529
lmo0645	lmo0645	lmo0607	lmo0607			lmo0531	lmo0531
lmo0646	lmo0646	lmo0609	lmo0609			lmo0532	lmo0532
lmo0648	lmo0648	lmo0611	lmo0611			lmo0535	lmo0535
lmo0650	lmo0650	lmo0612	lmo0612			lmo0539	lmo0539
lmo0651	lmo0651	lmo0613	lmo0613			lmo0540	lmo0540
thiD	lmo0662	lmo0616	lmo0616			lmo0551	lmo0551
lmo0663	lmo0663	lmo0617	lmo0617			lmo0571	lmo0571
lmo0674	lmo0674	lmo0618	lmo0618			lmo0573	lmo0573
lmo0721	lmo0721	lmo0619	lmo0619			lmo0586	lmo0586
lmo0727	lmo0727	lmo0620	lmo0620			lmo0599	lmo0599
lmo0764	lmo0764	lmo0621	lmo0621			lmo0600	lmo0600
lmo0774	lmo0774	lmo0622	lmo0622			lmo0602	lmo0602
lmo0777	lmo0777	lmo0623	lmo0623			lmo0606	lmo0606
lmo0781	lmo0781	lmo0624	lmo0624			lmo0608	lmo0608
lmo0782	lmo0782	lmo0625	lmo0625			lmo0618	lmo0618
lmo0783	lmo0783	lmo0628	lmo0628			lmo0621	lmo0621
lmo0784	lmo0784	lmo0632	lmo0632			lmo0629	lmo0629
lmo0788	lmo0788	lmo0637	lmo0637			lmo0633	lmo0633
lmo0794	lmo0794	lmo0641	lmo0641			lmo0634	lmo0634
lmo0796	lmo0796	lmo0644	lmo0644			lmo0635	lmo0635
lmo0799	lmo0799	lmo0646	lmo0646			lmo0636	lmo0636
lmo0813	lmo0813	lmo0648	lmo0648			lmo0638	lmo0638
uhpT	lmo0838	lmo0650	lmo0650			lmo0639	lmo0639
lmo0840	lmo0840	lmo0651	lmo0651			lmo0645	lmo0645
lmo0841	lmo0841	lmo0652	lmo0652			lmo0654	lmo0654
lmo0848	lmo0848	lmo0663	lmo0663			lmo0656	lmo0656
lmo0866	lmo0866	lmo0669	lmo0669			lmo0657	lmo0657
lmo0876	lmo0876	lmo0674	lmo0674			lmo0658	lmo0658
rsbU	lmo0892	lmo0695	lmo0695			lmo0659	lmo0659
rsbX	lmo0896	lmo0718	lmo0718			lmo0660	lmo0660
lmo0907	lmo0907	lmo0721	lmo0721			lmo0661	lmo0661
lmo0909	lmo0909	lmo0738	lmo0738			lmo0665	lmo0665
lmo0915	lmo0915	lmo0752	lmo0752			motB	lmo0686
lmo0927	lmo0927	lmo0754	lmo0754			lmo0694	lmo0694
lmo0937	lmo0937	lmo0762	lmo0762			lmo0697	lmo0697
lmo0939	lmo0939	lmo0764	lmo0764			lmo0701	lmo0701
fri	lmo0943	lmo0774	lmo0774			lmo0702	lmo0702
lmo0944	lmo0944	lmo0777	lmo0777			lmo0704	lmo0704
lmo0964	lmo0964	lmo0781	lmo0781			lmo0708	lmo0708
lmo0969	lmo0969	lmo0783	lmo0783			lmo0710	lmo0710
lmo0970	lmo0970	lmo0784	lmo0784			lmo0711	lmo0711
dltD	lmo0971	lmo0786	lmo0786			lmo0713	lmo0713
dltC	lmo0972	lmo0788	lmo0788			lmo0714	lmo0714
dltB	lmo0973	lmo0793	lmo0793			lmo0716	lmo0716
dltA	lmo0974	lmo0794	lmo0794			lmo0719	lmo0719
lmo0976	lmo0976	lmo0795	lmo0795			lmo0720	lmo0720
lmo0977	lmo0977	lmo0796	lmo0796			lmo0722	lmo0722
lmo0978	lmo0978	lmo0797	lmo0797			lmo0736	lmo0736
lmo0994	lmo0994	lmo0798	lmo0798			lmo0745	lmo0745
clpE	lmo0997	lmo0799	lmo0799			lmo0758	lmo0758
ptsH	lmo1002	lmo0803	lmo0803			lmo0763	lmo0763
lmo1003	lmo1003	lmo0804	lmo0804			lmo0770	lmo0770
lmo1006	lmo1006	lmo0813	lmo0813			lmo0782	lmo0782
lmo1011	lmo1011	lmo0837	lmo0837			lmo0787	lmo0787
lmo1051	lmo1051	uhpT	lmo0838			lmo0807	lmo0807
pdhC	lmo1054	lmo0840	lmo0840			lmo0809	lmo0809
lmo1067	lmo1067	lmo0841	lmo0841			lmo0815	lmo0815
lmo1070	lmo1070	lmo0842	lmo0842			lmo0816	lmo0816
lmo1075	lmo1075	lmo0848	lmo0848			lmo0823	lmo0823
lmo1081	lmo1081	ddlA	lmo0855			lmo0824	lmo0824
lmo1082	lmo1082	lmo0876	lmo0876			lmo0826	lmo0826
lmo1083	lmo1083	rsbU	lmo0892			lmo0834	lmo0834
lmo1084	lmo1084	lmo0897	lmo0897			lmo0844	lmo0844
lmo1086	lmo1086	lmo0900	lmo0900			lmo0851	lmo0851
lmo1087	lmo1087	lmo0905	lmo0905			lmo0854	lmo0854
lmo1093	lmo1093	lmo0907	lmo0907			lmo0857	lmo0857
lmo1094	lmo1094	lmo0909	lmo0909			lmo0858	lmo0858
guaA	lmo1096	lmo0910	lmo0910			lmo0860	lmo0860
lmo1099	lmo1099	lmo0911	lmo0911			lmo0862	lmo0862
lmo1102	lmo1102	lmo0915	lmo0915			lmo0865	lmo0865
lmo1163	lmo1163	lmo0916	lmo0916			lmo0868	lmo0868
lmo1179	lmo1179	lmo0927	lmo0927			lmo0869	lmo0869
lmo1207	lmo1207	lmo0933	lmo0933			lmo0879	lmo0879
pheT	lmo1222	lmo0936	lmo0936			lmo0885	lmo0885
lmo1250	lmo1250	lmo0937	lmo0937			lmo0898	lmo0898

lmo1272	lmo1272	fri	lmo0943	lmo0908	lmo0908
codV	lmo1277	lmo0944	lmo0944	lmo0914	lmo0914
lmo1290	lmo1290	lmo0947	lmo0947	lmo0923	lmo0923
lmo1291	lmo1291	lmo0950	lmo0950	lmo0924	lmo0924
glpD	lmo1293	lmo0952	lmo0952	lmo0940	lmo0940
miaA	lmo1294	lmo0957	lmo0957	lmo0942	lmo0942
lmo1297	lmo1297	lmo0964	lmo0964	lmo0960	lmo0960
glnR	lmo1298	lmo0969	lmo0969	lmo0966	lmo0966
glnA	lmo1299	lmo0970	lmo0970	dltA	lmo0974
lmo1301	lmo1301	dltD	lmo0971	lmo0986	lmo0986
lmo1302	lmo1302	dltC	lmo0972	lmo0987	lmo0987
lmo1306	lmo1306	lmo0976	lmo0976	lmo0988	lmo0988
smbA	lmo1313	lmo0977	lmo0977	lmo0989	lmo0989
frr	lmo1314	lmo0978	lmo0978	lmo0992	lmo0992
lmo1315	lmo1315	lmo0981	lmo0981	lmo0994	lmo0994
lmo1326	lmo1326	clpE	lmo0997	lmo1003	lmo1003
lmo1342	lmo1342	lmo1011	lmo1011	lmo1006	lmo1006
lmo1343	lmo1343	lmo1044	lmo1044	lmo1007	lmo1007
lmo1352	lmo1352	lmo1051	lmo1051	lmo1008	lmo1008
lmo1354	lmo1354	lmo1059	lmo1059	lmo1013	lmo1013
cspl	lmo1364	lmo1070	lmo1070	lmo1018	lmo1018
tcsA	lmo1388	lmo1075	lmo1075	lmo1024	lmo1024
lmo1395	lmo1395	lmo1077	lmo1077	lmo1025	lmo1025
mutl	lmo1404	lmo1081	lmo1081	lmo1031	lmo1031
pflB	lmo1406	lmo1082	lmo1082	lmo1041	lmo1041
pflC	lmo1407	lmo1083	lmo1083	lmo1066	lmo1066
lmo1423	lmo1423	lmo1084	lmo1084	lmo1067	lmo1067
lmo1424	lmo1424	lmo1087	lmo1087	lmo1086	lmo1086
opuCD	lmo1425	lmo1093	lmo1093	lmo1097	lmo1097
lmo1431	lmo1431	guaA	lmo1096	lmo1098	lmo1098
lmo1432	lmo1432	lmo1099	lmo1099	lmo1103	lmo1103
lmo1434	lmo1434	lmo1102	lmo1102	lmo1108	lmo1108
lmo1438	lmo1438	lmo1116	lmo1116	lmo1113	lmo1113
sod	lmo1439	lmo1151	lmo1151	lmo1118	lmo1118
lmo1440	lmo1440	lmo1163	lmo1163	lmo1127	lmo1127
lmo1466	lmo1466	lmo1179	lmo1179	lmo1130	lmo1130
lmo1468	lmo1468	lmo1180	lmo1180	lmo1131	lmo1131
dnaK	lmo1473	lmo1189	lmo1189	lmo1135	lmo1135
grpE	lmo1474	lmo1207	lmo1207	lmo1137	lmo1137
comEB	lmo1483	pheT	lmo1222	lmo1156	lmo1156
comEA	lmo1484	lmo1250	lmo1250	glpF	lmo1167
lmo1492	lmo1492	lmo1272	lmo1272	cbiE	lmo1195
udk	lmo1497	lmo1290	lmo1290	cbiH	lmo1199
lmo1499	lmo1499	lmo1291	lmo1291	cbiK	lmo1202
lmo1502	lmo1502	lmo1297	lmo1297	lmo1211	lmo1211
lmo1503	lmo1503	glnR	lmo1298	lmo1216	lmo1216
lmo1512	lmo1512	lmo1302	lmo1302	lmo1219	lmo1219
lmo1518	lmo1518	thrB	lmo1305	lmo1223	lmo1223
lmo1521	lmo1521	lmo1306	lmo1306	lmo1224	lmo1224
lmo1536	lmo1536	frr	lmo1314	lmo1231	lmo1231
lmo1537	lmo1537	lmo1315	lmo1315	lmo1253	lmo1253
lmo1538	lmo1538	lmo1317	lmo1317	lmo1256	lmo1256
rpmA	lmo1540	lmo1318	lmo1318	lmo1257	lmo1257
lmo1541	lmo1541	lmo1321	lmo1321	lmo1264	lmo1264
rplU	lmo1542	nusA	lmo1322	tig	lmo1267
mreC	lmo1547	infB	lmo1325	lmo1271	lmo1271
mreB	lmo1548	lmo1326	lmo1326	rnhB	lmo1273
dnaB	lmo1561	ribC	lmo1329	codV	lmo1277
lmo1562	lmo1562	lmo1342	lmo1342	lmo1292	lmo1292
citC	lmo1566	lmo1343	lmo1343	glnA	lmo1299
lmo1578	lmo1578	lmo1350	lmo1350	lmo1303	lmo1303
lmo1592	lmo1592	lmo1352	lmo1352	lmo1310	lmo1310
rpsD	lmo1596	lmo1354	lmo1354	lmo1323	lmo1323
aroA	lmo1600	cspl	lmo1364	rbfA	lmo1327
lmo1601	lmo1601	tkt	lmo1365	truB	lmo1328
lmo1602	lmo1602	lmo1405	lmo1405	pnpA	lmo1331
murC	lmo1605	pflB	lmo1406	lmo1332	lmo1332
lmo1606	lmo1606	pflC	lmo1407	lmo1345	lmo1345
lmo1615	lmo1615	lmo1414	lmo1414	lmo1356	lmo1356
lmo1616	lmo1616	lmo1415	lmo1415	lmo1369	lmo1369
lmo1634	lmo1634	lmo1423	lmo1423	lmo1371	lmo1371
lmo1639	lmo1639	lmo1424	lmo1424	lmo1372	lmo1372
citB	lmo1641	opuCD	lmo1425	lmo1373	lmo1373
lmo1643	lmo1643	opuCA	lmo1428	lmo1384	lmo1384
lmo1649	lmo1649	lmo1431	lmo1431	recA	lmo1398
lmo1650	lmo1650	lmo1432	lmo1432	lmo1401	lmo1401
tsf	lmo1657	lmo1433	lmo1433	lmo1410	lmo1410
rpsB	lmo1658	lmo1434	lmo1434	lmo1435	lmo1435
leuS	lmo1660	lmo1436	lmo1436	lmo1453	lmo1453
ansB	lmo1663	lmo1438	lmo1438	glyQ	lmo1459
lmo1665	lmo1665	smc	lmo1439	lmo1462	lmo1462
menD	lmo1675	zurM	lmo1446	lmo1463	lmo1463
lmo1677	lmo1677	rpoD	lmo1454	hrcA	lmo1475
lmo1687	lmo1687	lmo1466	lmo1466	rpsT	lmo1480
lmo1688	lmo1688	lmo1468	lmo1468	lmo1481	lmo1481
lmo1690	lmo1690	rpsU	lmo1469	comEA	lmo1484
lmo1693	lmo1693	lmo1470	lmo1470	lmo1506	lmo1506
lmo1695	lmo1695	grpE	lmo1474	apt	lmo1524
lmo1701	lmo1701	lepA	lmo1479	lmo1526	lmo1526
lmo1703	lmo1703	comEA	lmo1484	lmo1528	lmo1528
lmo1705	lmo1705	lmo1487	lmo1487	lmo1531	lmo1531
lmo1711	lmo1711	lmo1490	lmo1490	lmo1534	lmo1534
lmo1713	lmo1713	lmo1492	lmo1492	lmo1537	lmo1537
lmo1716	lmo1716	lmo1494	lmo1494	lmo1549	lmo1549
lmo1721	lmo1721	udk	lmo1497	thrS	lmo1559
lmo1722	lmo1722	lmo1499	lmo1499	lmo1562	lmo1562
lmo1729	lmo1729	lmo1500	lmo1500	lmo1577	lmo1577
lmo1741	lmo1741	lmo1501	lmo1501	lmo1582	lmo1582
adeC	lmo1742	lmo1502	lmo1502	argD	lmo1588
lmo1745	lmo1745	lmo1503	lmo1503	lmo1601	lmo1601
lmo1751	lmo1751	lmo1505	lmo1505	lmo1606	lmo1606

lmo1752	lmo1752	lmo1507	lmo1507	pheT	lmo1607
lmo1753	lmo1753	lmo1508	lmo1508	lmo1610	lmo1610
lmo1760	lmo1760	lmo1512	lmo1512	lmo1614	lmo1614
lmo1761	lmo1761	lmo1513	lmo1513	daaA	lmo1619
purD	lmo1764	lmo1518	lmo1518	lmo1621	lmo1621
lmo1780	lmo1780	lmo1530	lmo1530	trpC	lmo1630
lmo1782	lmo1782	ruvB	lmo1532	lmo1635	lmo1635
rplT	lmo1783	lmo1536	lmo1536	lmo1636	lmo1636
infC	lmo1785	lmo1538	lmo1538	lmo1638	lmo1638
inlC	lmo1786	rpmA	lmo1540	lmo1640	lmo1640
lmo1791	lmo1791	lmo1541	lmo1541	lmo1643	lmo1643
trmD	lmo1792	rplU	lmo1542	lmo1651	lmo1651
rpsP	lmo1797	minC	lmo1545	lmo1662	lmo1662
lmo1847	lmo1847	mreC	lmo1547	lmo1677	lmo1677
lmo1848	lmo1848	dnaB	lmo1561	lmo1679	lmo1679
lmo1849	lmo1849	citC	lmo1566	lmo1682	lmo1682
lmo1855	lmo1855	lmo1569	lmo1569	lmo1691	lmo1691
lmo1860	lmo1860	lmo1578	lmo1578	lmo1694	lmo1694
hup	lmo1934	argJ	lmo1590	lmo1696	lmo1696
lmo1938	lmo1938	lmo1592	lmo1592	lmo1703	lmo1703
resD	lmo1948	rpsD	lmo1596	lmo1719	lmo1719
lmo1955	lmo1955	aroA	lmo1600	lmo1771	lmo1771
fur	lmo1956	lmo1602	lmo1602	purB	lmo1773
lmo1962	lmo1962	lmo1603	lmo1603	purE	lmo1775
lmo1966	lmo1966	lmo1606	lmo1606	lmo1820	lmo1820
lmo1967	lmo1967	lmo1609	lmo1609	lmo1822	lmo1822
ilvN	lmo1985	lmo1612	lmo1612	lmo1854	lmo1854
alsS	lmo2006	lmo1615	lmo1615	lmo1859	lmo1859
cspB	lmo2016	lmo1616	lmo1616	lmo1868	lmo1868
divIVA	lmo2020	lmo1634	lmo1634	lmo1882	lmo1882
nadC	lmo2024	lmo1639	lmo1639	lmo1890	lmo1890
nadA	lmo2025	citB	lmo1641	recU	lmo1891
lmo2028	lmo2028	lmo1642	lmo1642	cca	lmo1905
lmo2029	lmo2029	lmo1645	lmo1645	lmo1908	lmo1908
lmo2030	lmo2030	lmo1646	lmo1646	lmo1909	lmo1909
pbpB	lmo2039	lmo1649	lmo1649	lmo1912	lmo1912
lmo2048	lmo2048	tsf	lmo1657	lmo1915	lmo1915
ctaA	lmo2058	rpsB	lmo1658	pflA	lmo1917
groEl	lmo2068	lmo1661	lmo1661	lmo1919	lmo1919
lmo2072	lmo2072	ansB	lmo1663	lmo1920	lmo1920
lmo2076	lmo2076	metK	lmo1664	lmo1922	lmo1922
lmo2077	lmo2077	lmo1665	lmo1665	hisC	lmo1925
lmo2078	lmo2078	lmo1666	lmo1666	lmo1933	lmo1933
lmo2083	lmo2083	lmo1671	lmo1671	lmo1951	lmo1951
lmo2101	lmo2101	lmo1683	lmo1683	fhuB	lmo1958
lmo2102	lmo2102	lmo1687	lmo1687	lmo1963	lmo1963
pta	lmo2103	lmo1690	lmo1690	lmo1969	lmo1969
lmo2114	lmo2114	lmo1693	lmo1693	lmo1974	lmo1974
lmo2115	lmo2115	lmo1695	lmo1695	lmo1975	lmo1975
lmo2119	lmo2119	lmo1697	lmo1697	lmo1982	lmo1982
lmo2120	lmo2120	lmo1701	lmo1701	ilvD	lmo1983
lmo2133	lmo2133	lmo1705	lmo1705	leuA	lmo1987
lmo2148	lmo2148	lmo1707	lmo1707	leuC	lmo1989
lmo2152	lmo2152	lmo1708	lmo1708	lmo1996	lmo1996
lmo2153	lmo2153	lmo1710	lmo1710	lmo2002	lmo2002
lmo2154	lmo2154	lmo1711	lmo1711	lmo2003	lmo2003
lmo2155	lmo2155	lmo1713	lmo1713	lmo2007	lmo2007
sepA	lmo2157	lmo1716	lmo1716	lmo2010	lmo2010
lmo2158	lmo2158	lmo1721	lmo1721	lmo2012	lmo2012
lmo2177	lmo2177	lmo1722	lmo1722	lmo2017	lmo2017
lmo2181	lmo2181	lmo1727	lmo1727	lmo2021	lmo2021
lmo2182	lmo2182	lmo1729	lmo1729	lmo2027	lmo2027
lmo2184	lmo2184	lmo1730	lmo1730	lmo2049	lmo2049
lmo2185	lmo2185	lmo1732	lmo1732	lmo2050	lmo2050
lmo2186	lmo2186	lmo1736	lmo1736	lmo2052	lmo2052
lmo2188	lmo2188	lmo1737	lmo1737	lmo2056	lmo2056
mecA	lmo2190	lmo1740	lmo1740	lmo2061	lmo2061
lmo2191	lmo2191	lmo1741	lmo1741	lmo2062	lmo2062
lmo2192	lmo2192	adeC	lmo1742	lmo2089	lmo2089
lmo2193	lmo2193	lmo1743	lmo1743	argH	lmo2091
lmo2194	lmo2194	lmo1745	lmo1745	lmo2097	lmo2097
lmo2195	lmo2195	lmo1747	lmo1747	lmo2098	lmo2098
lmo2196	lmo2196	lmo1750	lmo1750	lmo2100	lmo2100
trpS	lmo2198	lmo1751	lmo1751	lmo2105	lmo2105
lmo2199	lmo2199	lmo1752	lmo1752	lmo2116	lmo2116
lmo2201	lmo2201	lmo1753	lmo1753	lmo2118	lmo2118
lmo2202	lmo2202	gatA	lmo1755	lmo2121	lmo2121
lmo2203	lmo2203	lmo1760	lmo1760	lmo2122	lmo2122
lmo2204	lmo2204	lmo1761	lmo1761	lmo2140	lmo2140
lmo2205	lmo2205	purD	lmo1764	lmo2142	lmo2142
clpB	lmo2206	purH	lmo1765	lmo2144	lmo2144
lmo2207	lmo2207	purN	lmo1766	lmo2159	lmo2159
lmo2208	lmo2208	purM	lmo1767	lmo2184	lmo2184
lmo2209	lmo2209	purF	lmo1768	lmo2187	lmo2187
lmo2210	lmo2210	purI	lmo1770	lmo2188	lmo2188
hemH	lmo2211	purK	lmo1774	lmo2189	lmo2189
lmo2215	lmo2215	lmo1776	lmo1776	lmo2246	lmo2246
lmo2219	lmo2219	lmo1780	lmo1780	lmo2248	lmo2248
lmo2223	lmo2223	lmo1782	lmo1782	lmo2249	lmo2249
citG	lmo2225	rplT	lmo1783	lmo2252	lmo2252
lmo2234	lmo2234	infC	lmo1785	lmo2253	lmo2253
lmo2235	lmo2235	inlC	lmo1786	lmo2259	lmo2259
lmo2251	lmo2251	lmo1791	lmo1791	lmo2261	lmo2261
lmo2253	lmo2253	trmD	lmo1792	lmo2282	lmo2282
lmo2254	lmo2254	lmo1796	lmo1796	lmo2287	lmo2287
lmo2263	lmo2263	rpsP	lmo1797	lmo2291	lmo2291
lmo2267	lmo2267	lmo1802	lmo1802	lmo2295	lmo2295
lmo2269	lmo2269	lmo1803	lmo1803	lmo2296	lmo2296
lmo2277	lmo2277	smbA	lmo1804	lmo2303	lmo2303
lmo2360	lmo2360	rncS	lmo1805	lmo2306	lmo2306
pgi	lmo2367	fabG	lmo1807	lmo2309	lmo2309

lmo2376	lmo2376	lmo1811	lmo1811	lmo2311	lmo2311
lmo2384	lmo2384	lmo1813	lmo1813	lmo2312	lmo2312
lmo2386	lmo2386	lmo1814	lmo1814	lmo2318	lmo2318
lmo2390	lmo2390	lmo1817	lmo1817	lmo2324	lmo2324
lmo2391	lmo2391	fmt	lmo1823	lmo2326	lmo2326
ltrC	lmo2398	lmo1826	lmo1826	lmo2334	lmo2334
lmo2399	lmo2399	lmo1827	lmo1827	fruA	lmo2335
lmo2411	lmo2411	lmo1843	lmo1843	lmo2377	lmo2377
lmo2412	lmo2412	lmo1845	lmo1845	lmo2378	lmo2378
lmo2413	lmo2413	lmo1847	lmo1847	lmo2381	lmo2381
lmo2414	lmo2414	lmo1848	lmo1848	lmo2392	lmo2392
lmo2415	lmo2415	lmo1849	lmo1849	lmo2394	lmo2394
lmo2439	lmo2439	lmo1850	lmo1850	lmo2396	lmo2396
pgm	lmo2456	lmo1855	lmo1855	lmo2401	lmo2401
gap	lmo2459	deoD	lmo1856	lmo2402	lmo2402
lmo2471	lmo2471	lmo1860	lmo1860	lmo2405	lmo2405
trxB	lmo2478	lmo1862	lmo1862	lmo2427	lmo2427
uvrB	lmo2489	lmo1874	lmo1874	lmo2465	lmo2465
phoR	lmo2500	lmo1881	lmo1881	lmo2480	lmo2480
lmo2503	lmo2503	lmo1887	lmo1887	lmo2483	lmo2483
spl	lmo2505	pbpA	lmo1892	lmo2485	lmo2485
ftsX	lmo2506	panD	lmo1900	lmo2486	lmo2486
lmo2515	lmo2515	aroE	lmo1923	uvrA	lmo2488
lmo2518	lmo2518	aroF	lmo1928	lmo2493	lmo2493
lmo2522	lmo2522	ndk	lmo1929	lmo2496	lmo2496
murA	lmo2526	menH	lmo1931	lmo2497	lmo2497
atpD	lmo2529	lmo1932	lmo1932	lmo2502	lmo2502
fbaA	lmo2556	lmo1937	lmo1937	lmo2503	lmo2503
lmo2560	lmo2560	lmo1938	lmo1938	atpA	lmo2531
rpsI	lmo2596	resD	lmo1948	lmo2540	lmo2540
rplM	lmo2597	drm	lmo1954	lmo2555	lmo2555
rpsM	lmo2608	lmo1955	lmo1955	fbaA	lmo2556
infA	lmo2610	fur	lmo1956	lmo2579	lmo2579
rplO	lmo2613	lmo1962	lmo1962	lmo2583	lmo2583
rplX	lmo2621	lmo1966	lmo1966	lmo2592	lmo2592
rplV	lmo2627	lmo1967	lmo1967	rplF	lmo2617
rplB	lmo2629	lmo1970	lmo1970	rpsC	lmo2626
rplC	lmo2632	ilvN	lmo1985	rpsG	lmo2655
rpsJ	lmo2633	ilvC	lmo1986	rpsI	lmo2656
fus	lmo2654	ilvA	lmo1991	lmo2657	lmo2657
rpsG	lmo2655	lmo1992	lmo1992	lmo2661	lmo2661
lmo2691	lmo2691	lmo1997	lmo1997	lmo2662	lmo2662
lmo2692	lmo2692	cspB	lmo2016	lmo2676	lmo2676
lmo2696	lmo2696	lmo2018	lmo2018	lmo2693	lmo2693
lmo2697	lmo2697	nadC	lmo2024	lmo2695	lmo2695
recR	lmo2702	lmo2028	lmo2028	lmo2696	lmo2696
lmo2703	lmo2703	lmo2029	lmo2029	lmo2697	lmo2697
dnaX	lmo2704	lmo2030	lmo2030	lmo2699	lmo2699
lmo2713	lmo2713	ftsA	lmo2033	lmo2724	lmo2724
lmo2714	lmo2714	murG	lmo2035	lmo2725	lmo2725
cydD	lmo2715	murD	lmo2036	lmo2751	lmo2751
cydA	lmo2718	mraY	lmo2037	lmo2754	lmo2754
lmo2729	lmo2729	murE	lmo2038	topB	lmo2756
lmo2730	lmo2730	pbpB	lmo2039	lmo2757	lmo2757
lmo2731	lmo2731	lmo2042	lmo2042	lmo2772	lmo2772
lmo2770	lmo2770	lmo2048	lmo2048	lmo2779	lmo2779
lmo2779	lmo2779	ctaB	lmo2057	lmo2780	lmo2780
kat	lmo2785	lmo2067	lmo2067	lmo2781	lmo2781
lmo2794	lmo2794	groEl	lmo2068	lmo2795	lmo2795
		lmo2072	lmo2072	lmo2805	lmo2805
		lmo2076	lmo2076	lmo2807	lmo2807
		lmo2077	lmo2077	lmo2812	lmo2812
		lmo2078	lmo2078	lmo2816	lmo2816
		lmo2082	lmo2082	lmo2817	lmo2817
		lmo2083	lmo2083	lmo2828	lmo2828
		lmo2094	lmo2094	lmo2833	lmo2833
		lmo2099	lmo2099		
		lmo2101	lmo2101		
		lmo2102	lmo2102		
		lmo2112	lmo2112		
		lmo2113	lmo2113		
		lmo2114	lmo2114		
		lmo2119	lmo2119		
		lmo2120	lmo2120		
		lmo2133	lmo2133		
		lmo2135	lmo2135		
		lmo2145	lmo2145		
		lmo2149	lmo2149		
		lmo2150	lmo2150		
		lmo2151	lmo2151		
		lmo2153	lmo2153		
		lmo2154	lmo2154		
		lmo2155	lmo2155		
		sepA	lmo2157		
		lmo2158	lmo2158		
		lmo2177	lmo2177		
		lmo2178	lmo2178		
		lmo2182	lmo2182		
		lmo2185	lmo2185		
		lmo2186	lmo2186		
		mecA	lmo2190		
		lmo2191	lmo2191		
		lmo2192	lmo2192		
		lmo2193	lmo2193		
		lmo2195	lmo2195		
		lmo2196	lmo2196		
		trpS	lmo2198		
		lmo2199	lmo2199		
		lmo2200	lmo2200		
		lmo2201	lmo2201		
		lmo2202	lmo2202		

lmo2203	lmo2203
lmo2204	lmo2204
lmo2205	lmo2205
clpB	lmo2206
lmo2207	lmo2207
lmo2208	lmo2208
lmo2209	lmo2209
lmo2210	lmo2210
hemH	lmo2211
lmo2215	lmo2215
lmo2219	lmo2219
lmo2220	lmo2220
lmo2223	lmo2223
citG	lmo2225
lmo2230	lmo2230
lmo2233	lmo2233
lmo2234	lmo2234
lmo2235	lmo2235
arpJ	lmo2250
lmo2254	lmo2254
lmo2263	lmo2263
lmo2267	lmo2267
lmo2269	lmo2269
lmo2277	lmo2277
lmo2284	lmo2284
lmo2328	lmo2328
lmo2330	lmo2330
lmo2339	lmo2339
lmo2360	lmo2360
lmo2376	lmo2376
lmo2384	lmo2384
lmo2386	lmo2386
lmo2387	lmo2387
lmo2389	lmo2389
lmo2391	lmo2391
ltrC	lmo2398
lmo2399	lmo2399
lmo2411	lmo2411
lmo2412	lmo2412
lmo2413	lmo2413
lmo2414	lmo2414
lmo2415	lmo2415
lmo2417	lmo2417
lmo2418	lmo2418
lmo2422	lmo2422
lmo2431	lmo2431
lmo2434	lmo2434
lmo2438	lmo2438
lmo2440	lmo2440
lmo2442	lmo2442
lmo2444	lmo2444
lmo2446	lmo2446
lmo2447	lmo2447
lmo2448	lmo2448
lmo2450	lmo2450
lmo2451	lmo2451
lmo2453	lmo2453
lmo2454	lmo2454
eno	lmo2455
pgm	lmo2456
tpi	lmo2457
pgk	lmo2458
gap	lmo2459
lmo2460	lmo2460
clpP	lmo2468
lmo2469	lmo2469
lmo2471	lmo2471
lmo2472	lmo2472
lmo2473	lmo2473
lmo2474	lmo2474
trxB	lmo2478
lmo2481	lmo2481
uvrB	lmo2489
lmo2492	lmo2492
phoR	lmo2500
sod	lmo2505
ftsX	lmo2506
lmo2511	lmo2511
lmo2518	lmo2518
lmo2522	lmo2522
lmo2524	lmo2524
mbl	lmo2525
murA	lmo2526
atpD	lmo2529
atpB	lmo2535
prf1	lmo2543
suI	lmo2545
murZ	lmo2552
lmo2562	lmo2562
lmo2569	lmo2569
lmo2572	lmo2572
lmo2573	lmo2573
lmo2591	lmo2591
rpsI	lmo2596
rplM	lmo2597
lmo2599	lmo2599
lmo2601	lmo2601
lmo2603	lmo2603
rplQ	lmo2605
rpoA	lmo2606
rpsM	lmo2608

adk	lmo2611
secY	lmo2612
rplO	lmo2613
rpsE	lmo2615
rplR	lmo2616
rpsH	lmo2618
rplX	lmo2621
rplN	lmo2622
rplV	lmo2627
rpsS	lmo2628
rplB	lmo2629
rpsJ	lmo2633
lmo2635	lmo2635
lmo2636	lmo2636
lmo2652	lmo2652
tufA	lmo2653
fus	lmo2654
rpsG	lmo2655
lmo2679	lmo2679
kdpC	lmo2680
lmo2683	lmo2683
lmo2684	lmo2684
lmo2691	lmo2691
lmo2692	lmo2692
recR	lmo2702
lmo2703	lmo2703
dnaX	lmo2704
lmo2708	lmo2708
lmo2711	lmo2711
lmo2713	lmo2713
lmo2714	lmo2714
cydD	lmo2715
cydC	lmo2716
cydB	lmo2717
cydA	lmo2718
lmo2720	lmo2720
lmo2729	lmo2729
lmo2731	lmo2731
lmo2736	lmo2736
lmo2741	lmo2741
lmo2745	lmo2745
lmo2770	lmo2770
bvrC	lmo2786
lmo2794	lmo2794
gidB	lmo2802
lmo2821	lmo2821
lmo2822	lmo2822
lmo2829	lmo2829
lmo2837	lmo2837
lmo2842	lmo2842
lmo2845	lmo2845
lmo2851	lmo2851
