# Supplementary material for: In Vivo Transcriptional Profiling of Listeria monocytogenes and Mutagenesis Identify New Virulence Factors Involved in Infection
Source: PLoS Pathog. 2009 May 29;5(5):e1000449. doi: 10.1371/journal.ppat.1000449 (PMC2679221; doi:10.1371/journal.ppat.1000449)
Supplement: Table S3 — L. monocytogenes genes differentially regulated in the host as compared to exponential growth in BHI at 37°C (0.07 MB PDF) [file ppat.1000449.s005.pdf]

Table S3. *L. monocytogenes* EGDe genes differentially regulated in the host as compared to exponential growth in BHI at 37ºC

Gene designation	Gene	Annotation	Homolog in *L. innocua*	Fold change 24h	Fold change 48h	Fold change 72h
dnaA	lmo0001	chromosomal replication initiation protein DnaA	lin0001, dnaA		2,38	
recF	lmo0005	recombination protein F RecF	lin0005, recF		2,82	
gyrA	lmo0007	DNA gyrase subunit A	lin0007, gyrA		3,11	
lmo0010	lmo0010	similar to mevalonate kinase	lin0010		3,48	
mvaD	lmo0011	similar to mevalonate diphosphate decarboxylase	lin0011		2,35	
lmo0012	lmo0012	similar to mevalonate kinases	lin0012		3,40	
qoxA	lmo0013	AA3-600 quinol oxidase subunit II	lin0013, qoxA		2,94	
qoxB	lmo0014	AA3-600 quinol oxidase subunit I	lin0014, qoxB		2,42	
qoxD	lmo0016	highly similar to quinol oxidase aa3-600 chain IV	lin0016		2,99	
lmo0023	lmo0023	similar to PTS system, fructose-specific IIC component	lin0022			-2,05
lmo0024	lmo0024	similar to PTS system, mannose-specific IID component	lin0023			-2,88
lmo0032	lmo0032	similar to xylose repressor	lin0031			-2,30
lmo0033	lmo0033	similar to endoglucanase	lin0032			-2,10
ssb	lmo0045	highly similar to single-strand binding protein (SSB)	lin0038		7,98	3,41
rpsR	lmo0046	ribosomal protein S18	lin0039, rpsR		4,21	
rplI	lmo0053	50S ribosomal protein L9	lin0046, rplI		4,04	2,69
dnaC	lmo0054	replicative DNA helicase DnaC	lin0047			2,14
purA	lmo0055	adenylosuccinate synthetase	lin0048		11,87	
lmo0098	lmo0098	similar to PTS system mannose-specific, factor IID	lin0145		3,00	
lmo0150	lmo0150	unknown protein			16,53	
lmo0153	lmo0153	similar to a probable high-affinity zinc ABC transporter (Zn(II)-binding lipoprotein)	lin0191		5,32	
lmo0158	lmo0158	unknown protein	lin0196	2,45	4,48	
lmo0161	lmo0161	unknown protein	lin0204		3,72	
holB	lmo0162	DNA polymerase III subunit delta	lin0205	3,36	5,99	
lmo0165	lmo0165	unknown protein	lin0208		3,82	
lmo0169	lmo0169	similar to a glucose uptake protein	llin0212		3,11	
lmo0189	lmo0189	highly similar to B subtilis Veg protein	lin0228		10,91	
ispE	lmo0190	4-diphosphocytidyl-2-C-methyl-D-erythritol kinase	lin0229		2,15	
spoVG-1	lmo0196	similar to B subtilis SpoVG protein	lin0235		6,61	3,82
prfA	lmo0200	listeriolysin positive regulatory protein			8,09	4,00
plcA	lmo0201	phosphatidylinositol-specific phospholipase c		7,20	48,31	6,70
hly	lmo0202	listeriolysin O precursor		35,56	118,39	15,14
mpl	lmo0203	Zinc metalloproteinase precursor	lin0694	3,36	18,41	4,68
actA	lmo0204	actin-assembly inducing protein precursor		6,02	15,58	4,49
plcB	lmo0205	phospholipase C		12,37	106,64	31,78
lmo0206	lmo0206	unknown protein				3,11
lmo0207	lmo0207	hypothetical lipoprotein	lin0239		5,31	3,27
lmo0208	lmo0208	unknown protein	lin0240		3,95	
ctc	lmo0211	general stress protein Ctc	lin0243		5,81	
lmo0216	lmo0216	highly similar to B subtilis YabO protein	lin0248		4,09	
ftsH	lmo0220	highly similar to cell division protein ftsH	lin0252		7,96	2,95
lmo0222	lmo0222	molecular chaperone Hsp33	lin0254		2,17	
sul	lmo0224	highly similar to dihydropteroate synthases	lin0256		2,53	
folA	lmo0225	highly similar to dihydroneopterin aldolase	lin0257		2,84	
folK	lmo0226	similar to 7,8-dihydro-6-hydroxymethylpterin pyrophosphokinase	lin0258		2,29	
lmo0227	lmo0227	unknown protein	lin0259		4,03	
lysS	lmo0228	lysyl-tRNA synthetase	lin0260, lysS		3,77	
mcsA	lmo0230	similar to B subtilis YacH protein	lin0262		2,87	
mcsB	lmo0231	putative ATP:guanido phosphotransferase	lin0263	2,97	5,12	2,27
clpC	lmo0232	endopeptidase Clp ATP-binding chain C	lin0264, clpC	3,32	4,27	
ispF	lmo0236	2-C-methyl-D-erythritol 2,4-cyclodiphosphate synthase	lin0268, ispF		4,48	
gltX	lmo0237	highly similar to glutamyl-tRNA synthetase	lin0269		3,41	
secE	lmo0245	highly similar to preprotein translocase subunit	lin0277		2,21	
nusG	lmo0246	transcription antitermination protein NusG	lin0278		2,69	
rplK	lmo0248	ribosomal protein L11	lin0280, rplK	5,45	6,75	3,81
rplA	lmo0249	50S ribosomal protein L1	lin0281, rplA	9,90	12,55	3,07
rplJ	lmo0250	ribosomal protein L10	lin0282, rplJ	5,69	6,92	
rplL	lmo0251	50S ribosomal protein L7/L12	lin0283, rplL	4,79	5,49	2,03
lmo0257	lmo0257	unknown protein			2,12	
rpoC	lmo0259	DNA-directed RNA polymerase beta' subunit	lin0286, rpoC		10,44	2,87
inlH	lmo0263	internalin H		3,08	6,60	2,41
lmo0265	lmo0265	similar to succinyldiaminopimelate desuccinylase	lin0289		2,31	
lmo0278	lmo0278	similar to sugar ABC transporter, ATP-binding protein	lin0304		3,30	
ndrD	lmo0279	anaerobic ribonucleoside triphosphate reductase	lin0305		5,71	2,90
ndrG	lmo0280	highly similar to anaerobic ribonucleotide reductase activator protein	lin0306		6,99	2,52
lmo0301	lmo0301	similar to PTS beta-glucoside-specific enzyme IIA component	lin0329		9,46	
lmo0303	lmo0303	putaive secreted, lysin rich protein	lin0331		3,19	
lmo0355	lmo0355	fumarate reductase	lin0374	2,98	6,69	2,51
lmo0366	lmo0366	putative lipoprotein	lin0385		2,70	2,17
lmo0368	lmo0368	unknown protein	lin0387		5,02	
lmo0369	lmo0369	highly similar to B subtilis YeeI protein	lin0388		4,22	2,60
lmo0370	lmo0370	unknown protein	lin0389			2,76
lmo0391	lmo0391	unknown protein	lin0409	-4,68		-3,34
lmo0393	lmo0393	unknown protein	lin0411			-2,26
lmo0408	lmo0408	unknown protein	lin0431		3,44	
lmo0411	lmo0411	phosphoenolpyruvate synthase			4,76	
pgdA	lmo0415	similar to endo-1,4-beta-xylanase	lin0436		14,32	3,69
lstR	lmo0422	lineage-specific thermal regulator protein	lin0442, lstR		2,90	
inlA	lmo0433	Internalin A			15,92	3,86
inlB	lmo0434	Internalin B			3,82	2,55
lmo0439	lmo0439	weakly similar to a module of peptide synthetase	lin0460		2,76	
lmo0443	lmo0443	similar to B subtilis transcription regulator LytR	lin0463		2,41	
lmo0452	lmo0452	unknown protein	lin0469		2,96	
lmo0471	lmo0471	unknown protein			3,18	
lmo0481	lmo0481	unknown protein	lin0483	7,10	13,56	5,83
lmo0483	lmo0483	unknown protein	lin0485		6,02	
lmo0484	lmo0484	unknown protein	lin0487		2,66	
rpmF-1	lmo0486	ribosomal protein L32	lin0489, rpmF		2,88	
lmo0496	lmo0496	similar to B subtilis YnzC protein	lin0497			-2,00
prs	lmo0509	similar to phosphoribosyl pyrophosphate synthetase	lin0509		2,87	
lmo0512	lmo0512	unknown protein	lin0512	10,45	26,38	
lmo0516	lmo0516	similar to Bacillus anthracis encapsulation protein CapA	lin0516		14,26	
lmo0539	lmo0539	tagatose 1,6-diphosphate aldolase	lin0543	18,71	24,03	
lmo0540	lmo0540	similar to penicillin-binding protein	lin0544	3,77	3,96	2,29
lmo0541	lmo0541	similar to ABC transporter (binding protein)	lin0545	6,20	10,23	2,07
lmo0558	lmo0558	unknown protein	lin0567		2,33	
lmo0580	lmo0580	weakly similar to carboxylesterase	lin0589		2,69	
iap	lmo0582	P60 extracellular protein, invasion associated protein Iap	lin0591, iap	34,64	28,54	2,05
secA2	lmo0583	translocase	lin0592		7,41	
lmo0584	lmo0584	conserved hypothetical membrane protein	lin0593		3,16	
lmo0590	lmo0590	unknown protein	lin0599		3,48	
lmo0592	lmo0592	unknown protein	lin0601	33,87	18,41	
lmo0593	lmo0593	similar to transport proteins (formate?)	lin0602		2,32	
metX	lmo0594	homoserine O-acetyltransferase	lin0603	12,28	6,36	
lmo0596	lmo0596	unknown protein	lin0605	23,31	68,15	
lmo0597	lmo0597	similar to transcription regulator CRP/FNR family	lin0606	80,90	42,16	
lmo0599	lmo0599	unknown protein	lin0608	41,61	16,84	
lmo0600	lmo0600	unknown protein	lin0609	4,64	5,63	
lmo0601	lmo0601	similar to cell surface protein	lin0610	3,31	5,56	
lmo0604	lmo0604	similar to B subtilis YvlA protein	lin0613	51,45	27,43	
lmo0606	lmo0606	similar to transcription regulator MarR family	lin0615		2,93	
lmo0607	lmo0607	similar to ABC transporter, ATP-binding protein	lin0616		2,60	
lmo0608	lmo0608	similar to ABC transporter, ATP-binding protein	lin0617	12,16	7,07	
lmo0609	lmo0609	similar to E coli phage shock protein E	lin0618	13,83	6,76	
acpD	lmo0611	acyl carrier protein phosphodiesterase	lin0620, acpD	33,87	17,12	
lmo0612	lmo0612	similar to transcription regulator MarR family	lin0621	33,32	14,53	
lmo0618	lmo0618	similar to protein kinase	lin0627	40,02	19,89	
lmo0619	lmo0619	unknown protein	lin0628	32,90	16,81	
lmo0620	lmo0620	unknown protein	lin0629	9,63	5,51	
lmo0621	lmo0621	unknown protein	lin0630	102,28	47,85	
lmo0623	lmo0623	unknown protein	lin0632	97,66	43,52	
lmo0624	lmo0624	unknown protein	lin0633		4,74	

lmo0625	lmo0625	unknown protein	lin0634	25,50	13,86	
lmo0628	lmo0628	unknown protein	lin0637	17,27	9,55	
lmo0629	lmo0629	unknown protein	lin0638	3,97	3,63	
lmo0637	lmo0637	weakly similar to methyltransferase	lin0641	10,28	8,23	
lmo0639	lmo0639	similar to a transcription regulator (surface protein PAg negative regulator par)	lin0642	58,04	22,73	
lmo0644	lmo0644	unknown protein	lin0647	11,66	10,42	
lmo0645	lmo0645	similar to amino acid transporter	lin0648		2,94	
lmo0646	lmo0646	unknown protein	lin0649		5,52	
lmo0648	lmo0648	similar to membrane proteins	lin0651	83,42	35,45	
lmo0650	lmo0650	conserved membrane protein	lin0653	24,09	13,30	
lmo0651	lmo0651	similar to transcription regulator	lin0654	157,28	73,20	
thiD	lmo0662	highly similar to phosphomethylpyrimidine kinase thiD	lin0667		3,74	
lmo0663	lmo0663	unknown protein	lin0668		2,89	
mogR	lmo0674	unknown protein	lin0682		2,40	
flhF	lmo0681	similar to flagellar biosynthesis protein FlhF	lin0689			-2,04
flgE	lmo0697	flagellar hook protein FlgE	lin0705			-2,10
lmo0721	lmo0721	putative fibronectin-binding protein	lin0729	5,92	15,24	
glmS	lmo0727	D-fructose-6-phosphate amidotransferase	lin0734		9,82	2,78
lmo0764	lmo0764	similar to lipoate-protein ligase	lin0758		3,56	
lmo0774	lmo0774	unknown protein	lin0768		4,01	
lmo0777	lmo0777	unknown protein	lin0771		2,61	
lmo0781	lmo0781	similar to mannose-specific phosphotransferase system (PTS) component IID	lin0774		2,57	
lmo0782	lmo0782	similar to mannose-specific phosphotransferase system (PTS) component IIC	lin0775		4,69	
lmo0783	lmo0783	similar to mannose-specific phosphotransferase system (PTS) component IIB	lin0776		3,82	
lmo0784	lmo0784	similar to mannose-specific phosphotransferase system (PTS) component IIA	lin0777		2,89	
lmo0788	lmo0788	unknown protein	lin0781	60,06	31,36	20,50
lmo0794	lmo0794	similar to B subtilis YwnB protein	lin0787		4,47	
lmo0796	lmo0796	unknown protein	lin0789		4,21	
lmo0799	lmo0799	unknown protein	lin0792		3,02	
scrK	lmo0813	similar to fructokinases	lin0809	19,38	10,05	7,45
uhpT	lmo0838	hexose phosphate transport protein			5,13	2,59
lmo0840	lmo0840	unknown protein	lin0831	11,78	5,72	5,56
lmo0841	lmo0841	similar to cation (calcium) transporting ATPase	lin0832			2,06
lmo0848	lmo0848	similar to amino acid ABC transporter, ATP-binding protein	llin0841	20,94	10,77	8,22
lmo0866	lmo0866	similar to ATP-dependent RNA helicase	lin0859		3,38	
lmo0876	lmo0876	similar to PTS system, Lichenan-specific enzyme IIC component	lin0874	102,02	46,02	30,54
rsbU	lmo0892	highly similar to serine phosphatase RsbU	lin0891		2,44	
rsbX	lmo0896	Indirect negative regulation of sigma B dependant gene expression (serine phosphatase)	lin0895, rsbX		2,79	
lmo0907	lmo0907	similar to phosphoglycerate mutase	lin0907		3,12	
lmo0909	lmo0909	similar to transcription regulator, GntR family	lin0909	14,74	29,14	
lmo0915	lmo0915	similar to phosphotransferase system enzyme IIC		3,15		2,37
lmo0927	lmo0927	hypothetical transmembrane protein	lin0927		3,18	
lmo0937	lmo0937	unknown protein	lin0936		3,92	
lmo0939	lmo0939	unknown protein	lin0938		3,73	
fri	lmo0943	non-heme iron-binding ferritin	lin0942, fri		13,06	4,04
lmo0944	lmo0944	similar to B subtilis YneR protein	lin0943		2,93	
lmo0964	lmo0964	similar to B subtilis YjbH protein	lin0963		4,37	
lmo0969	lmo0969	similar to ribosomal large subunit pseudouridine synthetase	lin0968		6,37	
fabI	lmo0970	enoyl-(acyl carrier protein) reductase	lin0969		3,26	
dltD	lmo0971	DltD protein for D-alanine esterification of lipoteichoic acid and wall teichoic acid	lin0970, dltD	11,98	21,95	3,73
dltC	lmo0972	D-alanine--poly(phosphoribitol) ligase subunit 2 DltC	lin0971, dltC	4,13	6,22	2,35
dltB	lmo0973	DltB protein for D-alanine esterification of lipoteichoic acid and wall teichoic acid	lin0972, dltB	6,48	7,58	2,73
dltA	lmo0974	D-alanine--D-alanyl carrier protein ligase DltA	lin0973, dltA		6,37	3,85
lmo0976	lmo0976	similar to B subtilis YjcF protein	lin0975	7,06	4,60	3,54
lmo0977	lmo0977	similar to B subtilis YjcH protein	lin0976		2,57	
ilvE	lmo0978	branched-chain amino acid aminotransferase	lin0977	92,93	40,45	27,05
lmo0994	lmo0994	unknown protein	lin0993		2,53	
clpE	lmo0997	ATP-dependent protease	lin0996, clpE	2,64	6,31	2,41
ptsH	lmo1002	PTS phosphocarrier protein Hpr (histidine containing protein)	lin1001, ptsH	2,72	4,26	2,04
ptsI	lmo1003	phosphotransferase system enzyme I	lin1002		3,02	
lmo1006	lmo1006	similar to aminotransferases (to B subtilis PatA protein)	lin1005		2,36	
lmo1011	lmo1011	similar to tetrahydrodipicolinate succinylase	lin1010		4,72	
def	lmo1051	peptide deformylase	lin1043, def	40,34	19,14	13,47
pdhC	lmo1054	dihydrolipoamide dehydrogenase E3 subunit of pyruvate dehydrogenase complex	lin1046		8,99	2,25
typA	lmo1067	similar to GTP-binding elongation factor	lin1055		6,43	2,52
lmo1070	lmo1070	similar to B subtilis YlaN protein	lin1058	48,83	22,60	18,13
lmo1075	lmo1075	similar to teichoic acid translocation ATP-binding protein TagH (ABC transporter)	lin1063	9,28	5,48	4,20
lmo1081	lmo1081	similar to glucose-1-phosphate thymidyl transferase		8,25	5,99	5,17
lmo1082	lmo1082	similar to dTDP-sugar epimerase		47,21	24,06	21,01
lmo1083	lmo1083	similar to dTDP-D-glucose 4,6-dehydratase			3,49	
lmo1084	lmo1084	similar to DTDP-L-rhamnose synthetase			3,89	
ispD	lmo1086	2-C-methyl-D-erythritol 4-phosphate cytidylyltransferase	lin1071, ispD		2,66	
lmo1087	lmo1087	similar to glucitol dehydrogenase	lin1072	6,66	4,16	4,25
nadE	lmo1093	NAD(+) synthetase	lin1078, nadE		4,25	
lmo1094	lmo1094	unknown protein	lin1079		3,25	
guaA	lmo1096	bifunctional GMP synthase/glutamine amidotransferase protein	lin1081, guaA		2,72	
lmo1099	lmo1099	similar to a protein encoded by Tn916		65,42	30,42	24,04
lmo1102	lmo1102	similar to cadmium efflux system accessory proteins		9,71	4,51	4,72
pduS	lmo1142	similar to Salmonella enterica PduS protein	lin1106	-3,10		-2,44
pduT	lmo1143	similar to Salmonella enterica PduT protein	lin1107			-2,30
pduU	lmo1144	similar to Salmonella enterica PduU protein	lin1108			-2,87
pduV	lmo1145	similar to Salmonella enterica PduV protein	lin1109			-2,38
lmo1146	lmo1146	unknown protein	lin1110	-5,87	-5,33	-5,48
cobU	lmo1147	similar to bifunctional cobalamin biosynthesis protein CopB	lin1111	-5,13	-6,07	-5,61
cobS	lmo1148	highly similar to cobalamin (5'-phosphatase) synthetase	lin1112	-10,33	-12,33	-10,58
cobC	lmo1149	similar to alpha-ribazole-5'-phosphatase	lin1113	-5,60	-7,79	-5,85
pocR	lmo1150	Regulatory protein similar to Salmonella typhimurium PocR protein	lin1114	-4,06	-4,31	-3,42
pduA-1	lmo1151	similar to Salmonella typhimurium PduA protein	lin1115	-3,68	-4,62	-3,76
pduB	lmo1152	similar to Salmonella typhimurium PduB protein	lin1116	-10,81	-13,62	-12,38
pduC	lmo1153	highly similar to propanediol dehydratase, alpha subunit	lin1117	-5,91	-6,30	-5,72
pduD	lmo1154	similar to diol dehydrase (diol dehydratase) gamma subunit	lin1118			-2,14
pduG	lmo1156	similar to diol dehydratase-reactivating factor large subunit	lin1120	-4,26	-5,80	-5,79
pduH	lmo1157	similar to diol dehydratase-reactivating factor small chain	lin1121	-3,25	-3,47	-2,75
pduK	lmo1158	similar to Salmonella enterica PduK protein	lin1122	-6,22	-6,41	-4,61
pduA-2	lmo1159	similar to carboxysome structural protein	lin1123	-4,26	-3,96	-4,21
pduL	lmo1160	similar to Salmonella enterica PduL protein	lin1124	-4,97	-5,28	-4,84
eutJ	lmo1161	similar to ethanolamine utilization protein EutJ	lin1125	-14,00	-11,93	-13,46
pduM	lmo1162	similar to Salmonella enterica PduM propanediol utilization protein	lin1126	-4,91	-5,17	-4,40
eutN	lmo1163	similar to Salmonella enterica EutN ethanolamine utilization protein	lin1127	72,21	33,09	17,85
pduO	lmo1164	hyghly similar to Salmonella enterica PduO protein	lin1128	-3,84	-3,68	-3,40
eutE	lmo1165	similar to ethanolamine utilization protein EutE	lin1129	-8,66	-8,43	-8,28
pduQ-1	lmo1166	similar to Salmonella enterica pduQ putative propanol dehydrogenase	lin1130	-7,84	-8,03	-7,30
glpF	lmo1167	similar to glycerol uptake facilitator protein	lin1131	-11,94	-11,42	-12,30
AckA2	lmo1168	similar to acetate kinase	lin1132	-4,57	-4,59	-4,00
cobD	lmo1169	L-threonine-O-3-phosphate decarboxylase	lin1133	-4,49	-5,01	-4,58
pduX	lmo1170	similar to Salmonella enterica PduX protein	lin1134	-4,25	-5,16	-3,60
pduQ-2	lmo1171	similar to NADPH-dependent butanol dehydrogenase	lin1135	-5,33	-4,35	-5,80
lmo1172	lmo1172	similar to similar to two-component response regulator	lin1136	-4,15		-4,66
lmo1173	lmo1173	similar to two-component sensor histidine kinase	lin1137	-4,27	-4,20	-4,83
eutA	lmo1174	similar to ethanolamine utilization protein EutA (putative chaperonin)	lin1138	-6,39	-5,86	-6,60
eutB	lmo1175	similar to ethanolamine ammonia-lyase, heavy chain	lin1139			-2,32
eutC	lmo1176	ethanolamine ammonia-lyase small subunit	lin1140	-3,85		-4,50
eutL	lmo1177	similar to putative carboxysome structural protein (eutL)	lin1141	-4,88	-3,41	-6,35
lmo1179	lmo1179	similar to acetaldehyde dehydrogenase / alcohol dehydrogenase	lin1143	47,44	16,19	12,33
lmo1180	lmo1180	similar to putative carboxysome structural protein	lin1144	-3,29		-3,31
lmo1181	lmo1181	similar to cobalamin adenosyl transferase	lin1145	-3,31		-2,96
pduL-2	lmo1182	similar to Salmonella enterica PduL protein	lin1146	-3,18		-2,50
cbiA	lmo1191	cobyrinic acid a,c-diamide synthase	lin1154			-2,00
cbiB	lmo1192	cobalamin biosynthesis protein	lin1155, cbiB			-2,71
cbiD	lmo1194	cobalt-precorrin-6A synthase	lin1157			-2,16
cbiO	lmo1207	similar to cobalt transport ATP-binding protein CbiO	lin1170	7,58		18,32
pheT	lmo1222	phenylalanyl-tRNA synthetase beta subunit	lin1185, pheT		6,43	
lmo1250	lmo1250	similar to antibiotic resistance protein	lin1217			2,84
lmo1272	lmo1272	conserved hypothetical protein similar to B subtilis YlqF protein (ribosomal biogenesis GTPase)	lin1311		3,47	
codV	lmo1277	similar to integrase/recombinase	lin1316	5,91	2,69	18,12
lmo1290	lmo1290	similar to internalin proteins, putative peptidoglycan bound protein (LPXTG motif)				6,24

lmo1291	lmo1291	similar to acyltransferase (to B subtilis YrhL protein)	lin1329		4,73	
glpD	lmo1293	similar to glycerol 3 phosphate dehydrogenase	lin1331		3,47	
miaA	lmo1294	similar to tRNA isopentenylpyrophosphate transferase	lin1332		2,33	
lmo1297	lmo1297	similar to aluminum resistance protein and to B subtilis YnbB protein (hypothetical)	lin1335	17,17	10,49	41,32
glnR	lmo1298	similar to glutamine synthetase repressor	lin1336	3,25	8,22	2,27
glnA	lmo1299	highly similar to glutamine synthetases	lin1337	2,63	4,11	
lmo1301	lmo1301	unknown protein	lin1339		4,10	
lexA	lmo1302	LexA repressor	lin1340	6,20		13,96
lmo1306	lmo1306	highly similar to B subtilis YneF protein	lin1344	3,40	6,55	
pyrH	lmo1313	highly similar to uridylate kinases	lin1350		3,05	
frr	lmo1314	highly similar to ribosome recycling factors	lin1351		2,68	
uppS	lmo1315	similar to undecaprenyl diphosphate synthase	lin1352			2,16
lmo1326	lmo1326	conserved hypothetical protein similar to B subtilis YlxP protein	lin1363	11,24	4,33	30,57
lmo1342	lmo1342	similar to B subtilis comG operon protein 6	lin1379		2,43	7,23
comGE	lmo1343	similar to comG operon protein 5 (comGE)	lin1380			8,30
lmo1352	lmo1352	unknown protein	lin1389	7,23		14,73
lmo1354	lmo1354	similar to aminopeptidase P	lin1391	4,97	2,97	11,70
cspL	lmo1364	similar to cold shock protein	lin1401			2,02
tcsA	lmo1388	CD4+ T cell-stimulating antigen, lipoprotein	lin1425, tcsA			2,19
lmo1395	lmo1395	unknown protein	lin1432		7,80	2,42
mutL	lmo1404	DNA mismatch repair protein	lin1441, mutL		3,63	
pflB	lmo1406	pyruvate formate-lyase	lin1443, pflB		4,32	
pflC	lmo1407	pyruvate-formate lyase activating enzyme	lin1444, pflC		2,66	
lmo1423	lmo1423	unknown protein	lin1462	4,22	26,53	3,15
lmo1424	lmo1424	putative manganese transport protein MntH	lin1463		10,22	2,67
opuCD	lmo1425	osmoprotectant transport system permease protein	lin1464		2,69	
lmo1431	lmo1431	similar to ABC transporter (ATP-binding protein)	lin1470		3,45	
lmo1432	lmo1432	unknown protein	lin1471		3,25	
lmo1434	lmo1434	unknown protein	lin1473		4,17	
lmo1438	lmo1438	similar to penicillin-binding protein	lin1477		3,55	
sod	lmo1439	superoxide dismutase	lin1478, sod	4,51	13,84	
lmo1440	lmo1440	unknown protein	lin1479		2,42	
lmo1466	lmo1466	unknown protein	lin1503	6,36		17,07
lmo1468	lmo1468	unknown protein	lin1505		6,78	2,48
dnaK	lmo1473	class I heat-shock protein (molecular chaperone) DnaK	lin1510, dnaK		6,69	
grpE	lmo1474	heat shock protein GrpE	lin1511, grpE			2,07
comEB	lmo1483	similar to B subtilis ComEB protein	lin1518			5,13
comEA	lmo1484	similar to integral membrane protein ComEA	lin1519	5,50		14,06
lmo1492	lmo1492	unknown protein	lin1527	21,55	6,50	52,50
udk	lmo1497	uridine kinase	lin1532	21,03	7,30	44,64
lmo1499	lmo1499	unknown protein	lin1534		3,54	
lmo1502	lmo1502	unknown protein	lin1537		4,21	
lmo1503	lmo1503	unknown protein	lin1538		2,57	
trmU	lmo1512	similar to putative tRNA (5-methylaminomethyl-2-thiouridylate)-methyltransferase	lin1547			7,38
lmo1518	lmo1518	lmo1518	lin1553		2,95	
lmo1521	lmo1521	similar to N-acetylmuramoyl-L-alanine amidase	lin1556		2,73	
pheA	lmo1536	prephenate dehydratase	lin1571		3,49	
lmo1537	lmo1537	conserved GTP binding protein	lin1572		4,55	
glpK	lmo1538	glycerol kinase	lin1573		2,59	
rpmA	lmo1540	50S ribosomal protein L27	lin1575, rpmA		3,96	
lmo1541	lmo1541	unknown protein	lin1576	2,95	4,02	
rplU	lmo1542	ribosomal protein L21	lin1577, rplU	4,71	8,12	2,22
mreB	lmo1547	similar to cell-shape determining protein MreB	lin1581		2,44	
mreC	lmo1548	similar to cell-shape determining protein MreC	lin1582		4,07	
dnaB	lmo1561	chromosome replication initiation / membrane attachment protein DnaB	lin1596, dnaB			5,31
lmo1562	lmo1562	transcriptional regulator NrdR	lin1597		6,57	
citC	lmo1566	isocitrate dehydrogenase	lin1601			2,04
pepQ	lmo1578	similar to X-Pro dipeptidase	lin1613			5,25
thiI	lmo1592	similar to thiamin biosynthesis protein ThiI	lin1634		6,37	
rpsD	lmo1596	30S ribosomal protein S4	lin1638, rpsD	7,35	14,30	3,06
aroA	lmo1600	3-deoxy-7-phosphoheptulonate synthase	lin1641, aroA		4,08	2,27
lmo1601	lmo1601	similar to general stress protein	lin1642			2,18
lmo1602	lmo1602	unknown protein	lin1643		4,54	2,13
murC	lmo1605	UDP-N-acetylmuramate--L-alanine ligase	lin1646			2,40
lmo1606	lmo1606	similar to DNA translocase	lin1647		7,73	
trmB	lmo1615	tRNA (guanine-N(7)-)-methyltransferase	lin1656		6,71	
lmo1616	lmo1616	unknown protein	lin1657		2,87	
adhE	lmo1634	similar to Alcohol-acetaldehyde dehydrogenase	lin1675	4,81	12,76	3,38
tag	lmo1639	similar to dna-3-methyladenine glycosidase	lin1680		4,30	
citB	lmo1641	aconitate hydratase	lin1682		2,14	
lmo1643	lmo1643	unknown protein	lin1684		5,10	
lmo1649	lmo1649	unknown protein	lin1689		3,38	
lmo1650	lmo1650	unknown protein	lin1690		2,33	
tsf	lmo1657	elongation factor Ts EF-Ts	lin1766, tsf		4,79	2,49
rpsB	lmo1658	30S ribosomal protein S2	lin1767, rpsB		7,52	5,82
leuS	lmo1660	leucyl-tRNA synthetase	lin1769, leuS		2,38	
lmo1665	lmo1665	unknown protein	lin1774		3,30	
menD	lmo1675	2-oxoglutarate decarboxylase	lin1783		3,31	
lmo1677	lmo1677	1,4-dihydroxy-2-naphthoate octaprenyltransferase	lin1785		3,19	
lmo1687	lmo1687	unknown protein	lin1795		4,01	8,56
lmo1688	lmo1688	enoyl-(acyl carrier protein) reductase	lin1796		2,70	
lmo1690	lmo1690	unknown protein	lin1798		6,10	
recX	lmo1693	recombination regulator RecX	lin1801		2,40	
mprF	lmo1695	similar to MprF protein of S aureus	lin1803	9,68	4,59	24,61
lmo1701	lmo1701	unknown protein	lin1809		2,83	
lmo1703	lmo1703	similar to similar to RNA methyltransferases	lin1815		3,52	
lmo1705	lmo1705	similar to deoxyguanosine kinase/deoxyadenosine kinase(I) subunit	lin1817		4,97	
lmo1711	lmo1711	highly similar to aminopeptidases	lin1823		2,77	
lmo1713	lmo1713	similar to cell-shape determining protein mreB	lin1825		5,82	
lmo1716	lmo1716	similar to putative transcription regulators	lin1827			8,61
lmo1721	lmo1721	similar to transcriptional regulator (NifA/NtrC family)	lin1832			2,04
lmo1722	lmo1722	similar to ATP-dependent RNA helicases	lin1833		3,69	
lmo1728	lmo1728	some similarities to cellobiose-phosphorylase	lin1839			-2,03
bglX	lmo1729	similar to beta-glucosidases	lin1840	8,84		24,80
virS	lmo1741	similar to two-component sensor histidine kinase	lin1852		2,76	
adeC	lmo1742	highly similar to adenine deaminases	lin1853		2,66	
virR	lmo1745	similar to two-component response regulator	lin1856		5,96	2,29
lmo1751	lmo1751	similar to hypothetical RNA methyltransferase	lin1863		3,41	
lmo1752	lmo1752	unknown protein	lin1864		3,44	
lmo1753	lmo1753	unknown protein	lin1865			8,41
pcrB	lmo1760	PcrB-like protein	lin1872			2,78
lmo1761	lmo1761	similar to putative sodium-dependent transporter	lin1873	16,59	5,42	42,54
purD	lmo1764	phosphoribosylglycinamide synthetase	lin1876, purD			2,26
pepT	lmo1780	peptidase T	lin1892		2,52	
exoA	lmo1782	similar to 3'-exo-deoxyribonuclease exoA	lin1894		2,35	
rplT	lmo1783	50S ribosomal protein L20	lin1895, rplT		2,61	
infC	lmo1785	translation initiation factor IF-3	lin1897, infC		5,70	2,06
inlC	lmo1786	internalin C			8,56	3,57
lmo1791	lmo1791	unknown protein	lin1905		2,25	
trmD	lmo1792	similar to E coli tRNA (guanine-N1) methyltransferase	lin1906		4,03	
rpsP	lmo1797	ribosomal protein S16	lin1911, rpsP	3,17	4,96	2,01
sdhA	lmo1812	similar to L-serine dehydratase	lin1926	-4,06	-3,82	-4,17
lmo1817	lmo1817	weakly similar to thiamin pyrophosphokinase	lin1931			-3,10
rpe-2	lmo1818	ribulose-phosphate 3-epimerase	lin1932			-2,04
lmo1819	lmo1819	ribosome-associated GTPase	lin1933			-3,22
lmo1821	lmo1821	similar to putative phosphoprotein phosphatase	lin1935	-4,75	-4,43	-4,31
sun	lmo1822	similar to RNA-binding Sun protein	lin1936			-2,52
fmt	lmo1823	similar to methionyl-tRNA formyltransferase	lin1937			-3,42
priA	lmo1824	primosome assembly protein PriA	lin1938	-3,66	-4,02	-4,11
coaBC	lmo1825	similar to pantothenate metabolism flavoprotein homolog	lin1939			-2,27
lmo1828	lmo1828	unknown protein	lin1942			-2,69
fbpA	lmo1829	similar to fibronectin binding proteins	lin1943	-3,50		-3,61
lmo1830	lmo1830	unknown protein	lin1944			-2,56
pyrE	lmo1831	orotate phosphoribosyltransferase	lin1945, pyrE			-3,55
pyrF	lmo1832	highly similar to orotidine 5'-phosphate decarboxylases	lin1946	-3,09		-3,42
pyrD	lmo1833	highly similar to dihydroorotase dehydrogenase	lin1947			-3,24

pyrDII	lmo1834	highly similar to dihydroorotate dehydrogenase (electron transfer subunit)	lin1948	-5,85	-6,38	-6,12
pyrAb	lmo1835	carbamoyl-phosphate synthase large subunit	lin1949, carB	-4,27	-4,09	-4,06
pyrAa	lmo1836	carbamoyl-phosphate synthase small subunit	lin1950	-3,67	-3,58	-3,78
pyrC	lmo1837	dihydroorotase	lin1951, pyrC	-3,70	-4,14	-3,72
pyrP	lmo1839	highly similar to uracil permease	lin1953	-3,45	-3,35	-3,52
lspA	lmo1844	signal peptidase II	lin1958, lspA			-2,01
lmo1845	lmo1845	unknown protein	lin1959			-2,13
lpeA	lmo1847	similar to adhesion binding proteins and lipoproteins	lin1961	6,55	31,08	10,64
lmo1848	lmo1848	similar metal cations ABC transporter (permease protein)	lin1962		9,93	2,86
lmo1849	lmo1849	similar to metal cations ABC transporter, ATP-binding proteins	lin1963	3,34	17,86	5,57
lmo1851	lmo1851	similar to carboxy-terminal processing proteinase	lin1965			-2,12
lmo1855	lmo1855	similar to similar to D-alanyl-D-alanine carboxypeptidases	lin1969		3,31	
msrA	lmo1860	methionine sulfoxide reductase A	lin1974		2,64	
lmo1869	lmo1869	similar to conserved hypothetical proteins, putative integral membrane protein	lin1983			-2,41
lmo1871	lmo1871	similar to phosphoglucomutases	lin1985	-3,93	-4,23	-3,77
lmo1872	lmo1872	similar to methyltransferases	lin1986	-3,80	-4,10	-3,89
lmo1883	lmo1883	similar to chitinases	lin1996			-2,44
ansB	lmo1896	asparaginyl-tRNA synthetase	lin2010		5,15	
hup	lmo1934	similar to non-specific DNA-binding protein HU	lin2048		5,21	
rpsA	lmo1938	similar to ribosomal protein S1	lin2052		5,25	
resD	lmo1948	similar to two-component response regulator (ResD)	lin2062		4,09	
xerD	lmo1955	similar to integrase/recombinase	lin2069		2,76	
fur	lmo1956	similar to transcriptional regulator (Fur family)	lin2070		7,68	2,71
lmo1962	lmo1962	similar to transcription regulators (TetR family)	lin2076		2,83	
lmo1966	lmo1966	unknown protein	lin2080		3,15	
lmo1967	lmo1967	similar to toxic ion resistance proteins	lin2081		4,73	
ilvN	lmo1985	similar to acetolactate synthase (acetohydroxy-acid synthase) (small subunit)	lin2092		8,42	
alsS	lmo2006	alpha-acetolactate synthase	lin2114		2,43	
cspB	lmo2016	similar to major cold-shock protein	lin2124		3,66	
divIVA	lmo2020	similar to cell-division initiation protein (septum placement)	lin2128		3,30	
nadC	lmo2024	nicotinate-nucleotide pyrophosphorylase	lin2132		6,56	
nadA	lmo2025	quinolinate synthetase	lin2133		4,07	
lmo2028	lmo2028	unknown protein	lin2134		5,25	
lmo2029	lmo2029	unknown protein	lin2135	4,35	6,03	
lmo2030	lmo2030	unknown protein	lin2136		2,14	
pbpB	lmo2039	penicilin binding protein 2B	lin2145		5,00	
rpmF-2	lmo2047	50S ribosomal protein L32	lin2153, rpmF		1,78	
lmo2048	lmo2048	unknown protein	lin2154		4,93	2,51
ctaA	lmo2058	similar to heme O oxygenase	lin2164		3,59	
groEL	lmo2068	chaperonin GroEL	lin2174, groEL		5,59	2,01
rex	lmo2072	redox-sensing transcriptional repressor Rex	lin2178		2,68	
rimI	lmo2076	similar to ribosomal protein alanine acetyltransferase	lin2182		2,53	
lmo2077	lmo2077	similar to glycoprotease	lin2183		6,60	
lmo2078	lmo2078	unknown protein	lin2184		4,68	
lmo2083	lmo2083	unknown protein	lin2189		6,47	
lmo2101	lmo2101	pyridoxine biosynthesis protein	lin2205		2,93	
lmo2102	lmo2102	glutamine amidotransferase subunit PdxT	lin2206	2,77	4,10	
lmo2114	lmo2114	similar to ABC transporter (ATP-binding protein)	lin2219		3,68	2,93
lmo2115	lmo2115	similar to ABC transporter (permease)	lin2220	4,86	5,92	3,06
lmo2119	lmo2119	unknown protein	lin2224		4,43	
lmo2120	lmo2120	unknown protein	lin2225		2,37	
lmo2133	lmo2133	similar to fructose-1,6-biphosphate aldolase type II	lin2238		5,19	
lmo2148	lmo2148	unknown protein	lin2252		2,93	
lmo2152	lmo2152	similar to thioredoxin	lin2256		5,98	2,27
lmo2153	lmo2153	flavodoxin	lin2257		10,57	4,55
nrdF	lmo2154	ribonucleotide-diphosphate reductase beta subunit	lin2258		5,82	2,13
nrdA	lmo2155	ribonucleotide-diphosphate reductase alpha subunit	lin2259		7,83	2,93
lmo2157	lmo2157	unknown protein		3,29	5,85	2,01
lmo2158	lmo2158	similar to B subtilis YwmG protein	lin2261	3,02	12,61	
lmo2175	lmo2175	3-ketoacyl-(acyl-carrier-protein) reductase	lin2278			-2,34
lmo2177	lmo2177	unknown protein	lin2280	3,36	6,14	2,23
srtB	lmo2181	sortase B	lin2285		2,44	
lmo2182	lmo2182	similar to ferrichrome ABC transporter (ATP-binding protein)	lin2286		2,77	
lmo2184	lmo2184	similar to ferrichrome ABC transporter (binding protein)	lin2288		5,11	2,56
svpA	lmo2185	unknown protein	lin2289	6,61	11,11	
lmo2186	lmo2186	unknown protein	lin2290		2,72	
pepF	lmo2188	similar to oligoendopeptidase	lin2292		4,36	
mecA	lmo2190	adaptor protein	lin2294, mecA	3,55	4,49	2,18
spxA	lmo2191	transcriptional regulator Spx	lin2295, spxA		4,53	
lmo2192	lmo2192	similar to oligopeptide ABC transporter (ATP-binding protein)	lin2296	7,80	7,22	
lmo2193	lmo2193	similar to oligopeptide ABC transporter (ATP-binding protein)	lin2297		4,39	
lmo2194	lmo2194	similar to oligopeptide ABC transporter (permease)	lin2298	3,37	3,36	2,48
lmo2195	lmo2195	similar to oligopeptide ABC transporter (permease)	lin2299	7,32	6,47	
oppA	lmo2196	similar to pheromone ABC transporter (binding protein)	lin2300	5,20	6,16	2,73
trpS	lmo2198	tryptophanyl-tRNA synthetase	lin2301, trpS		3,30	
lmo2199	lmo2199	unknown protein	lin2302	4,61	6,66	
fabF	lmo2201	similar to 3-oxoacyl-acyl-carrier protein synthase	lin2304		3,90	2,03
fabH	lmo2202	3-oxoacyl-(acyl carrier protein) synthase	lin2305		3,51	
lmo2203	lmo2203	similar to N-acetylmuramoyl-L-alanine amidase	lin2306		4,50	
lmo2204	lmo2204	unknown protein	lin2307	5,71	4,31	
gpm	lmo2205	similar to phosphoglyceromutase 1	lin2308	4,08	8,42	2,15
clpB	lmo2206	similar to endopeptidase Clp ATP-binding chain B (ClpB)	lin2309		3,90	
lmo2207	lmo2207	unknown protein	lin2310		4,48	
lmo2208	lmo2208	unknown protein	lin2311		2,70	
lmo2209	lmo2209	unknown protein	lin2312	4,32	4,41	
lmo2210	lmo2210	unknown protein	lin2313	9,23	10,20	
hemH	lmo2211	ferrochelatase	lin2314	4,71	6,76	
lmo2215	lmo2215	similar to ABC transporter (ATP-binding protein)	lin2318		7,42	
prsA2	lmo2219	similar to post-translocation molecular chaperone	lin2322	12,02	24,00	8,95
lmo2223	lmo2223	unknown protein	lin2326		3,46	
citG	lmo2225	similar to fumarate hydratase	lin2328		2,34	
lmo2234	lmo2234	unknown protein	lin2336		6,74	
lmo2235	lmo2235	similar to NADH oxidase	lin2337	6,92	7,95	
lmo2251	lmo2251	similar to amino acid ABC transporter (ATP-binding protein)	lin2353		2,22	
lmo2253	lmo2253	similar to phosphoglucomutase	lin2355		4,54	
lmo2254	lmo2254	unknown proteins	lin2356		4,85	
lmo2257	lmo2257	hypothetical CDS		-4,62		-3,99
lmo2263	lmo2263	unknown protein	lin2364		3,49	
addA	lmo2267	similar to ATP-dependent deoxyribonuclease (subunit A)	lin2368		2,59	
lmo2269	lmo2269	unknown protein	lin2370		3,01	
lmo2276	lmo2276	similar to an unknown bacteriophage protein				-2,72
lmo2277	lmo2277	unknown protein			2,61	
lmo2360	lmo2360	transmembrane protein	lin2460			2,80
pgi	lmo2367	glucose-6-phosphate isomerase	lin2466, pgi		5,83	2,23
lmo2376	lmo2376	similar to peptidyl-prolyl cis-trans isomerase	lin2475		4,14	
lmo2384	lmo2384	similar to proteins involved in resistance to cholate and to NA(+) and in pH homeostasis	lin2483		2,83	
lmo2386	lmo2386	similar to B subtilis YuiD protein	lin2485		3,63	
lmo2390	lmo2390	similar to hypothetical thioredoxine reductase	lin2489		2,19	
lmo2391	lmo2391	conserved hypothetical protein similar to B subtilis YhfK protein	lin2490		4,56	
ltrC	lmo2398	low temperature requirement C protein, also similar to B subtilis YutG protein	lin2497, ltrC		2,94	
lmo2399	lmo2399	unknown protein	lin2498	3,09	12,36	3,39
lmo2411	lmo2411	Fe-S cluster assembly protein SufB	lin2506		12,44	5,12
lmo2412	lmo2412	nitrogen fixation protein NifU and related proteins	lin2507		7,65	2,64
lmo2413	lmo2413	selenocysteine lyase	lin2508		8,46	3,93
lmo2414	lmo2414	FeS assembly protein SufD	lin2509			3,78
lmo2415	lmo2415	Fe-S cluster assembly ATP-binding protein	lin2510			2,50
gcvH	lmo2425	similar to glycine cleavage system protein H	lin2519			-2,13
lmo2432	lmo2432	unknown protein	lin2526			-2,39
lmo2437	lmo2437	unknown protein	lin2531	-3,20		-4,03
lmo2439	lmo2439	unknown protein	lin2533		4,44	
lmo2454	lmo2454	unknown protein	lin2548			-2,47
pgm	lmo2456	phosphoglyceromutase	lin2550		6,29	
gap	lmo2459	glyceraldehyde-3-phosphate dehydrogenase	lin2553		4,49	2,37
lmo2471	lmo2471	similar to NADH oxidase	lin2614		5,00	
trxB	lmo2478	thioredoxin reductase	lin2621, trxB		2,63	
uvrB	lmo2489	excinuclease ABC subunit B	lin2632, uvrB		2,91	

phoR	lmo2500	two-component sensor histidine kinase	lin2643, phoR		2,52	
lmo2503	lmo2503	similar to cardiolipin synthase	lin2646		3,06	
spl	lmo2505	peptidoglycan lytic protein P45	lin2648, spl		12,21	2,68
ftsX	lmo2506	highly similar to cell-division protein FtsX	lin2649			3,88
degU	lmo2515	similar to B subtilis two-component response regulator DegU	lin2659			2,11
lmo2518	lmo2518	similar to B subtilis putative transcriptional regulator LytR	lin2662		5,03	
lmo2522	lmo2522	similar to hypothetical cell wall binding protein from B subtilis	lin2666		5,72	3,07
lmo2526	lmo2526	UDP-N-acetylglucosamine 1-carboxyvinyltransferase	lin2670			2,40
atpD	lmo2529	ATP synthase subunit B	lin2673		5,08	2,72
fbaA	lmo2556	similar to fructose-1,6-bisphosphate aldolase	lin2701		5,73	
lmo2560	lmo2560	similar to B subtilis RNA polymerase delta subunit	lin2705			2,06
lmo2574	lmo2574	unknown protein	lin2719			-2,51
lmo2583	lmo2583	similar to two-component response regulator	lin2728			-2,18
lmo2586	lmo2586	similar to formate dehydrogenase alpha chain	lin2731			-2,52
rpsI	lmo2596	30S ribosomal protein S9	lin2745, rpsI	3,55	9,70	
rplM	lmo2597	50S ribosomal protein L13	lin2746, rplM	5,02	12,78	3,29
rpsM	lmo2608	30S ribosomal protein S13	lin2757, rpsM		5,49	2,62
infA	lmo2610	translation initiation factor IF-1	lin2759		3,07	
rplO	lmo2613	ribosomal protein L15	lin2762, rplO		2,86	
rplX	lmo2621	50S ribosomal protein L24	lin2770, rplX	6,39	5,44	2,75
rplV	lmo2627	50S ribosomal protein L22	lin2776, rplV	12,44	12,21	
rplB	lmo2629	50S ribosomal protein L2	lin2778, rplB			2,05
rplC	lmo2632	ribosomal protein L3	lin2781, rplC	8,50	8,99	3,25
rpsJ	lmo2633	30S ribosomal protein S10	lin2782, rpsJ			2,22
lmo2642	lmo2642	unknown protein	lin2791			-2,13
lmo2644	lmo2644	unknown protein	lin2793	-3,30		-2,75
lmo2645	lmo2645	unknown protein	lin2794			-2,53
lmo2646	lmo2646	unknown protein	lin2795			-3,67
lmo2648	lmo2648	similar to Phosphotriesterase	lin2797	-3,24	-3,53	-3,28
ulaA	lmo2649	ascorbate-specific PTS system enzyme IIC	lin2798, ulaA			-2,76
fus	lmo2654	elongation factor EF-2	lin2803	5,37	6,18	2,75
rpsG	lmo2655	30S ribosomal protein S7	lin2804, rpsG	4,30	4,42	
dgt	lmo2657	deoxyguanosinetriphosphate triphosphohydrolase	lin2806			-2,29
tkt	lmo2660	similar to transketolase	lin2809	-3,02		-2,75
lmo2661	lmo2661	similar to ribulose-5-phosphate 3-epimerase	lin2810	-3,21		-2,81
rpiB-2	lmo2662	similar to ribose 5-phosphate epimerase	lin2811			-2,24
lmo2663	lmo2663	similar to polyol dehydrogenase	lin2812	-4,79	-4,12	-4,56
lmo2664	lmo2664	similar to sorbitol dehydrogenase	lin2813	-3,84		-3,27
lmo2665	lmo2665	similar to PTS system galactitol-specific enzyme IIC component	lin2814			-3,08
lmo2669	lmo2669	unknown protein	lin2818			-2,06
lmo2672	lmo2672	weakly similar to transcription regulator				-2,31
rpiB-4	lmo2674	ribose-5-phosphate isomerase B	lin2821			-2,67
lmo2676	lmo2676	similar to UV-damage repair protein	lin2823			-2,11
kdpB	lmo2681	potassium-transporting ATPase subunit B	lin2829, kdpB			-2,16
murA	lmo2691	autolysin, N-acetylmuramidase	lin2838		4,06	
lmo2692	lmo2692	unknown protein	lin2840		3,91	
lmo2696	lmo2696	similar to hypothetical dihydroxyacetone kinase	lin2844		2,49	
lmo2697	lmo2697	unknown protein	lin2845		3,14	
recR	lmo2702	recombination protein RecR	lin2850, recR		2,79	
lmo2703	lmo2703	highly similar to B subtilis YaaK protein	lin2851		2,95	
dnaX	lmo2704	highly similar to DNA polymerase III (gamma and tau subunits)	lin2852		2,76	
lmo2713	lmo2713	secreted protein with 1 GW repeat	lin2861	8,86	17,08	2,80
lmo2714	lmo2714	peptidoglycan anchored protein (LPXTG motif)	lin2862	25,45	70,27	3,09
cydD	lmo2715	highly similar to ABC transporter (ATP-binding protein) required for expression of cytochrome BD	lin2863		3,04	
cydA	lmo2718	highly similar to cytochrome D ubiquinol oxidase subunit I	lin2866		3,70	
lmo2729	lmo2729	unknown protein	lin2877		5,42	
lmo2730	lmo2730	similar to phosphatase	lin2878		2,15	
lmo2731	lmo2731	similar to transcription regulator (RpiR family)	lin2879		2,63	
lmo2733	lmo2733	similar to PTS system, fructose-specific IIABC component				-2,35
lmo2736	lmo2736	glycerate kinase				-2,48
lmo2741	lmo2741	similar to drug-efflux transporters	lin2884			-2,17
lmo2759	lmo2759	unknown protein	lin2902			-2,36
lmo2761	lmo2761	similar to beta-glucosidase	lin2904			-2,43
lmo2763	lmo2763	similar to PTS cellobiose-specific enzyme IIC	lin2906			-2,16
lmo2770	lmo2770	putative glutamate--cysteine ligase	lin2913		3,14	
lmo2779	lmo2779	similar to probable GTP-binding protein	lin2919		3,79	
kat	lmo2785	catalase	lin2920, kat		6,45	2,68
lmo2794	lmo2794	chromosome partitioning protein, ParB family	lin2926		3,17	
