# Supplementary material for: In Vivo Transcriptional Profiling of Listeria monocytogenes and Mutagenesis Identify New Virulence Factors Involved in Infection
Source: PLoS Pathog. 2009 May 29;5(5):e1000449. doi: 10.1371/journal.ppat.1000449 (PMC2679221; doi:10.1371/journal.ppat.1000449)
Supplement: Table S4 — L. monocytogenes EGDe genes encoding proteins of the cell wall subproteome and differentially regulated in the host (0.03 MB PDF) [file ppat.1000449.s006.pdf]

Table S4. *L. monocytogenes* EGDe genes encoding proteins of the cell wall subproteome and differentially regulated in the host

Gene designation	Gene	Annotation	Homolog in *L. innocua*	Fold change 24h	Fold change 48h	Fold change 72h
qoxA	lmo0013	AA3-600 quinol oxidase subunit II.	lin0013, qoxA		2,95	
inlB	lmo0434	Internalin B			3,84	2,55
lmo0443	lmo0443	similar to B. subtilis transcription regulator LytR				
iap	lmo0582	P60 extracellular protein, invasion associated protein Iap	lin0591, iap	34,54	28,64	2,04
pdhC	lmo1054	dihydrolipoamide dehydrogenase E3 subunit of pyruvate dehydrogenase complex	lin1047		9,00	2,25
lmo1291	lmo1291	similar to acyltransferase (to B subtilis YrhL protein)	lin1329		4,72	
frr	lmo1314	highly similar to ribosome recycling factors	lin1351		2,68	
tcsA	lmo1388	CD4+ T cell-stimulating antigen, lipoprotein	lin1425, tcsA			2,19
sod	lmo1439	superoxide dismutase.	lin1478, sod	4,50	13,83	
dnaK	lmo1473	class I heat-shock protein (molecular chaperone) DnaK.	lin1510, dnaK		6,68	
adhE	lmo1634	similar to Alcohol-acetaldehyde dehydrogenase.	lin1675	4,79	12,73	3,39
tsf	lmo1657	elongation factor Ts EF-Ts	lin1766, tsf		4,79	2,50
lpeA	lmo1847	similar to adhesion binding proteins and lipoproteins	lin1961	6,54	31,12	10,63
lmo1967	lmo1967	similar to toxic ion resistance proteins	lin2081		4,72	
groEL	lmo2068	chaperonin GroEL	lin2174, groEL		5,58	2,01
prsA2	lmo2219	similar to post-translocation molecular chaperone.	lin1482; lin2322	12,04	23,92	8,94
lmo2415	lmo2415	similar to ABC transporter, ATP-binding protein.	lin2510			2,50
gcvH	lmo2425	similar to glycine cleavage system protein H	lin2519			-2,13
pgm	lmo2456	phosphoglyceromutase	lin2550		6,28	
gap	lmo2459	glyceraldehyde-3-phosphate dehydrogenase	lin2553		4,50	2,36
spl	lmo2505	peptidoglycan lytic protein P45	lin2648, spl		12,21	2,68
fbaA	lmo2556	similar to fructose-1,6-bisphosphate aldolase.	lin2701		5,74	
fus	lmo2654	elongation factor EF-2	lin2803	5,35	6,19	2,75
murA	lmo2691	autolysin, N-acetylmuramidase	lin2838		4,06	
