# Supplementary material for: In Vivo Transcriptional Profiling of Listeria monocytogenes and Mutagenesis Identify New Virulence Factors Involved in Infection
Source: PLoS Pathog. 2009 May 29;5(5):e1000449. doi: 10.1371/journal.ppat.1000449 (PMC2679221; doi:10.1371/journal.ppat.1000449)
Supplement: Table S5 — L. monocytogenes genes encoding secreted proteins and differentially regulated in the host (0.03 MB PDF) [file ppat.1000449.s007.pdf]

Table S5. *L. monocytogenes* EGDe genes encoding secreted proteins and differentially regulated in the host

Gene designation	Gene	Annotation	Homolog in *L. innocua*	Fold change 24h	Fold change 48h	Fold change 72h
Proteins present in culture supernatant [34]						
qoxA	lmo0013	AA3-600 quinol oxidase subunit II	lin0013, qoxA		2,95	
lmo0153	lmo0153	similar to a probable high-affinity zinc ABC transporter (Zn(II)-binding lipoprotein)	lin0191		5,31	
plcA	lmo0201	phosphatidylinositol-specific phospholipase c		7,21	48,17	6,68
hly	lmo0202	listeriolysin O precursor		35,51	118,60	15,14
mpl	lmo0203	Zinc metalloproteinase precursor	lin0694	3,36	18,38	4,69
actA	lmo0204	actin-assembly inducing protein precursor		6,02	15,56	4,50
plcB	lmo0205	phospholipase C		12,38	106,89	31,78
rplL	lmo0251	50S ribosomal protein L7/L12	lin0283, rplL	4,79	5,50	2,03
inlH	lmo0263	internalin H		3,07	6,59	2,41
inlA	lmo0433	Internalin A			15,89	3,86
inlB	lmo0434	Internalin B			3,84	2,55
lmo0443	lmo0443	similar to B subtilis transcription regulator LytR	lin0463		2,41	
lmo0539	lmo0539	tagatose 1,6-diphosphate aldolase	lin0543	18,77	24,08	
iap	lmo0582	P60 extracellular protein, invasion associated protein Iap	lin0591, iap	34,54	28,64	2,04
lmo0644	lmo0644	unknown protein	lin0647	11,63	10,41	
lmo0796	lmo0796	unknown protein	lin0789		4,20	
lmo0927	lmo0927	hypothetical transmembrane protein	lin0927		3,18	
fri	lmo0943	non-heme iron-binding ferritin	lin0942, fri		13,09	4,06
ptsH	lmo1002	PTS phosphocarrier protein Hpr (histidine containing protein)	lin1001, ptsH	2,71	4,26	2,04
lmo1291	lmo1291	similar to acyltransferase (to B subtilis YrhL protein)	lin1329		4,72	
tcsA	lmo1388	CD4+ T cell-stimulating antigen, lipoprotein	lin1425, tcsA			2,19
lmo1395	lmo1395	unknown protein	lin1432		7,78	2,41
sod	lmo1439	superoxide dismutase	lin1478, sod	4,50	13,83	
dnaK	lmo1473	class I heat-shock protein (molecular chaperone) DnaK	lin1510, dnaK		6,68	
lmo1521	lmo1521	similar to N-acetylmuramoyl-L-alanine amidase	lin1556		2,73	
tsf	lmo1657	elongation factor Ts EF-Ts	lin1766, tsf		4,79	2,50
lmo1752	lmo1752	unknown protein	lin1864		3,43	
inlC	lmo1786	internalin C			8,57	3,58
lpeA	lmo1847	similar to adhesion binding proteins and lipoproteins	lin1961	6,54	31,12	10,63
lmo1851	lmo1851	similar to carboxy-terminal processing proteinase	lin1965			-2,11
lmo1883	lmo1883	similar to chitinases	lin1996			-2,45
hup	lmo1934	similar to non-specific DNA-binding protein HU	lin2048		5,21	
cspB	lmo2016	similar to major cold-shock protein	lin2124		3,66	
groEL	lmo2068	chaperonin GroEL	lin2174, groEL		5,58	2,01
svpA	lmo2185	unknown protein	lin2289	6,59	11,08	
lmo2186	lmo2186	unknown protein	lin2290		2,71	
oppA	lmo2196	similar to pheromone ABC transporter (binding protein)	lin2300	5,21	6,15	2,73
gcvH	lmo2425	similar to glycine cleavage system protein H	lin2519			-2,13
gap	lmo2459	glyceraldehyde-3-phosphate dehydrogenase	lin2553		4,50	2,36
spl	lmo2505	peptidoglycan lytic protein P45	lin2648, spl		12,21	2,68
lmo2518	lmo2518	similar to B subtilis putative transcriptional regulator LytR	lin2662		5,03	
lmo2522	lmo2522	similar to hypothetical cell wall binding protein from B subtilis	lin2666		5,74	3,07
kdpB	lmo2681	potassium-transporting ATPase subunit B	lin2829, kdpB			-2,16
murA	lmo2691	autolysin, N-acetylmuramidase	lin2838		4,06	
lmo2714	lmo2714	peptidoglycan anchored protein (LPXTG motif)	lin2862	25,46	70,03	3,10
Proteins secreted by the SecA2 system [24,36]						
rpoC	lmo0259	DNA-directed RNA polymerase beta' subunit	lin0286, rpoC		10,44	2,87
iap	lmo0582	P60 extracellular protein, invasion associated protein Iap	lin0591, iap	34,54	28,64	2,04
pdhC	lmo1054	dihydrolipoamide dehydrogenase E3 subunit of pyruvate dehydrogenase complex	lin1047		8,99	2,25
tcsA	lmo1388	CD4+ T cell-stimulating antigen, lipoprotein	lin1425, tcsA			2,19
sod	lmo1439	superoxide dismutase	lin1478, sod	4,50	13,83	
dnaK	lmo1473	class I heat-shock protein (molecular chaperone) DnaK	lin1510, dnaK		6,68	
groEL	lmo2068	chaperonin GroEL	lin2174, groEL		5,58	2,01
oppA	lmo2196	similar to pheromone ABC transporter (binding protein)	lin2300	5,21	6,15	2,73
rpsI	lmo2596	30S ribosomal protein S9	lin2745, rpsI	3,55	9,70	
murA	lmo2691	autolysin, N-acetylmuramidase	lin2838		4,06	
