# Supplementary material for: In Vivo Transcriptional Profiling of Listeria monocytogenes and Mutagenesis Identify New Virulence Factors Involved in Infection
Source: PLoS Pathog. 2009 May 29;5(5):e1000449. doi: 10.1371/journal.ppat.1000449 (PMC2679221; doi:10.1371/journal.ppat.1000449)
Supplement: Table S6 — L. monocytogenes genes involved in DNA metabolism, RNA and protein synthesis, cell division and multiplication, and up regulated in the host (0.03 MB PDF) [file ppat.1000449.s008.pdf]

Table S6. *L. monocytogenes* EGDe genes involved in DNA metabolism, RNA and protein synthesis, cell division and multiplication, and up-regulated in the host

Gene designation	Gene	Annotation	Homolog in *L. innocua*	Fold change 24h	Fold change 48h	Fold change 72h
DNA synthesis						
holB	lmo0162	DNA polymerase III subunit delta	lin0205	3,36	5,99	
dnaX	lmo2704	highly similar to DNA polymerase III (gamma and tau subunits)	lin2852		2,76	
DNA restriction/modifications and repair						
mutL	lmo1404	DNA mismatch repair protein	lin1441, mutL		3,63	
tag	lmo1639	similar to dna-3-methyladenine glycosidase	lin1680		4,30	
exoA	lmo1782	similar to 3'-exo-deoxyribonuclease exoA	lin1894		2,35	
uvrB	lmo2489	excinuclease ABC subunit B	lin2632, uvrB		2,91	
DNA recombination						
recF	lmo0005	recombination protein F RecF	lin0005, recF		2,82	
codV	lmo1277	similar to integrase/recombinase	lin1316	5,91	2,69	18,12
recX	lmo1693	recombination regulator RecX	lin1801		2,40	
addA	lmo2267	similar to ATP-dependent deoxyribonuclease (subunit A)	lin2368		2,59	
recR	lmo2702	recombination protein RecR	lin2850, recR		2,79	
DNA packaging and segregation						
gyrA	lmo0007	DNA gyrase subunit A	lin0007, gyrA		3,11	
lmo1606	lmo1606	similar to DNA translocase	lin1647		7,73	
hup	lmo1934	similar to non-specific DNA-binding protein HU	lin2048		5,21	
lmo2794	lmo2794	chromosome partitioning protein, ParB family	lin2926		3,17	
Ribosomal proteins						
rpsR	lmo0046	ribosomal protein S18	lin0039, rpsR		4,21	
rplI	lmo0053	50S ribosomal protein L9	lin0046, rplI		4,04	2,69
rplK	lmo0248	ribosomal protein L11	lin0280, rplK	5,45	6,75	3,81
rplA	lmo0249	50S ribosomal protein L1	lin0281, rplA	9,90	12,55	3,07
rplJ	lmo0250	ribosomal protein L10	lin0282, rplJ	5,69	6,92	
rplL	lmo0251	50S ribosomal protein L7/L12	lin0283, rplL	4,79	5,49	2,03
rpmF-1	lmo0486	ribosomal protein L32	lin0489, rpmF		2,88	
rpmA	lmo1540	50S ribosomal protein L27	lin1575, rpmA		3,96	
rplU	lmo1542	ribosomal protein L21	lin1577, rplU	4,71	8,12	2,22
rpsD	lmo1596	30S ribosomal protein S4	lin1638, rpsD	7,35	14,30	3,06
rpsB	lmo1658	30S ribosomal protein S2	lin1767, rpsB		7,52	5,82
rplT	lmo1783	50S ribosomal protein L20	lin1895, rplT		2,61	
rpsP	lmo1797	ribosomal protein S16	lin1911, rpsP	3,17	4,96	2,01
rpsA	lmo1938	similar to ribosomal protein S1	lin2052		5,25	
rpmF-2	lmo2047	50S ribosomal protein L32	lin2153, rpmF		1,78	
rpsI	lmo2596	30S ribosomal protein S9	lin2745, rpsI	3,55	9,70	
rplM	lmo2597	50S ribosomal protein L13	lin2746, rplM	5,02	12,78	3,29
rpsM	lmo2608	30S ribosomal protein S13	lin2757, rpsM		5,49	2,62
rplO	lmo2613	ribosomal protein L15	lin2762, rplO		2,86	
rplX	lmo2621	50S ribosomal protein L24	lin2770, rplX	6,39	5,44	2,75
rplV	lmo2627	50S ribosomal protein L22	lin2776, rplV	12,44	12,21	

rplB	lmo2629	50S ribosomal protein L2	lin2778, rplB			2,05
rplC	lmo2632	ribosomal protein L3	lin2781, rplC	8,50	8,99	3,25
rpsJ	lmo2633	30S ribosomal protein S10	lin2782, rpsJ			2,22
rpsG	lmo2655	30S ribosomal protein S7	lin2804, rpsG	4,30	4,42	
Protein synthesis initiation, elongation and termination						
typA	lmo1067	similar to GTP-binding elongation factor	lin1055		6,43	2,52
frr	lmo1314	highly similar to ribosome recycling factors	lin1351		2,68	
tsf	lmo1657	elongation factor Ts EF-Ts	lin1766, tsf		4,79	2,49
infC	lmo1785	translation initiation factor IF-3	lin1897, infC		5,70	2,06
infA	lmo2610	translation initiation factor IF-1	lin2759		3,07	
fus	lmo2654	elongation factor EF-2	lin2803	5,37	6,18	2,75
Chromosomal replication and segregation						
dnaA	lmo0001	chromosomal replication initiation protein DnaA	lin0001, dnaA		2,38	
ssb	lmo0045	highly similar to single-strand binding protein (SSB)	lin0038		7,98	3,41
dnaC	lmo0054	replicative DNA helicase DnaC	lin0047			2,14
dnaB	lmo1561	chromosome replication initiation / membrane attachment protein DnaB	lin1596, dnaB			5,31
divIVA	lmo2020	similar to cell-division initiation protein (septum placement)	lin2128		3,30	
Cell elongation and division						
spoVG-1	lmo0196	similar to B subtilis SpoVG protein	lin0235		6,61	3,82
ftsH	lmo0220	highly similar to cell division protein ftsH	lin0252		7,96	2,95
mreB	lmo1547	similar to cell-shape determining protein MreB	lin1581		2,44	
mreC	lmo1548	similar to cell-shape determining protein MreC	lin1582		4,07	
ftsX	lmo2506	highly similar to cell-division protein FtsX	lin2649			3,88
