# Supplementary material for: In Vivo Transcriptional Profiling of Listeria monocytogenes and Mutagenesis Identify New Virulence Factors Involved in Infection
Source: PLoS Pathog. 2009 May 29;5(5):e1000449. doi: 10.1371/journal.ppat.1000449 (PMC2679221; doi:10.1371/journal.ppat.1000449)
Supplement: Table S7 — L. monocytogenes genes involved in stress responses and differentially regulated in the host (0.04 MB PDF) [file ppat.1000449.s009.pdf]

Table S7. *L. monocytogenes* EGDe genes involved in stress responses and differentially regulated in the host

Gene designation	Gene	Annotation	Homolog in *L. innocua*	Fold change 24h	Fold change 48h	Fold change 72h
**Class I**						
HrcA down regulated						
lmo0355	lmo0355	fumarate reductase	lin0374	2,97	6,68	2,51
glpD	lmo1293	similar to glycerol 3 phosphate dehydrogenase	lin1331		3,46	
pflC	lmo1407	pyruvate-formate lyase activating enzyme	lin1444, pflC		2,66	
dnaK	lmo1473	class I heat-shock protein (molecular chaperone) DnaK	lin1510, dnaK		6,68	
grpE	lmo1474	heat shock protein GrpE	lin1511, grpES			2,07
adhE	lmo1634	similar to Alcohol-acetaldehyde dehydrogenase	lin1675	4,79	12,73	3,39
groEL	lmo2068	chaperonin GroEL	lin2174, groEL		5,58	2,01
lmo2269	lmo2269	unknown protein	lin2370		3,01	
gap	lmo2459	glyceraldehyde-3-phosphate dehydrogenase	lin2553		4,50	2,36
HrcA up regulated						
dnaA	lmo0001	chromosomal replication initiation protein DnaA	lin0001, dnaA		2,38	
rpsR	lmo0046	ribosomal protein S18	lin0039, rpsR		4,20	
rplK	lmo0248	ribosomal protein L11	lin0280, rplK	5,46	6,73	3,81
rplA	lmo0249	50S ribosomal protein L1	lin0281, rplA	9,92	12,55	3,07
rpmF-1	lmo0486	ribosomal protein L32	lin0489, rpmF		2,89	
glmS	lmo0727	D-fructose-6-phosphate amidotransferase	lin0734		9,85	2,77
lmo1468	lmo1468	unknown protein	lin1505		6,77	2,48
lmo1541	lmo1541	unknown protein	lin1576	2,95	4,03	
rplU	lmo1542	ribosomal protein L21	lin1577, rplU		8,11	2,22
tsf	lmo1657	elongation factor Ts. EF-Ts	lin1766, tsf		4,79	2,50
rpsB	lmo1658	30S ribosomal protein S2	lin1767, rpsB		7,52	5,82
infC	lmo1785	translation initiation factor IF-3	lin1897, infC		5,70	2,06
fur	lmo1956	similar to transcriptional regulator Fur	lin2070		7,67	2,71
rpmF-2	lmo2047	50S ribosomal protein L32	lin2153, rpmF		1,78	
lmo2048	lmo2048	unknown protein	lin2154		4,92	2,51
trxB	lmo2478	thioredoxin reductase	lin2621, trxB		2,64	
rplM	lmo2597	50S ribosomal protein L13	lin2746, rplM		12,82	3,29
**Class II**						
SigB up regulated						
lmo0169	lmo0169	similar to a glucose uptake protein	llin0212		3,12	
inlH	lmo0263	internalin H		3,07	6,59	2,41
lmo0265	lmo0265	similar to succinyldiaminopimelate desuccinylase	lin0289		2,31	
lmo0439	lmo0439	weakly similar to a module of peptide synthetase	lin0460		2,77	
lmo0539	lmo0539	similar to tagatose-1,6-diphosphate aldolase	lin0543	18,77	24,08	
lmo0580	lmo0580	weakly similar to carboxylesterase	lin0589		2,69	
lmo0590	lmo0590	unknown protein	lin0599		3,48	
lmo0593	lmo0593	similar to transport proteins (formate?)	lin0602		2,31	
lmo0596	lmo0596	unknown protein	lin0605	23,26	68,12	
lmo0781	lmo0781	similar to mannose-specific phosphotransferase system (PTS) component IID	lin0774		2,57	
lmo0782	lmo0782	similar to mannose-specific phosphotransferase system (PTS) component IIC	lin0775		4,69	
lmo0783	lmo0783	similar to mannose-specific phosphotransferase system (PTS) component IIB	lin0776		3,84	
lmo0784	lmo0784	similar to mannose-specific phosphotransferase system (PTS) component IIA	lin0777		2,89	
lmo0794	lmo0794	similar to B. subtilis YwnB protein	lin0787		4,47	
lmo0796	lmo0796	unknown protein	lin0789		4,20	
lmo0994	lmo0994	unknown protein	lin0993		2,53	
clpE	lmo0997	ATP-dependent protease	lin0996, clpE	2,64	6,32	2,41
pdhC	lmo1054	dihydrolipoamide dehydrogenase E3 subunit of pyruvate dehydrogenase complex	lin1047		9,00	2,25
AckA2	lmo1168	similar to acetate kinase	lin1132	-4,56	-4,59	-4,00
lmo1601	lmo1601	similar to general stress protein	lin1642			2,19
lmo1602	lmo1602	unknown protein	lin1643		4,53	2,13
lmo1606	lmo1606	similar to DNA translocase	lin1647		7,73	
lmo1830	lmo1830	unknown protein	lin1944			-2,57
lmo1883	lmo1883	similar to chitinases	lin1996			-2,45
lmo2157	lmo2157	unknown protein		3,29	5,86	2,01
lmo2158	lmo2158	similar to B. subtilis YwmG protein	lin2261	3,01	12,64	
lmo2175	lmo2175	similar to dehydrogenase	lin2278			-2,33
gpm	lmo2205	similar to phosphoglyceromutase 1	lin2308	4,08	8,40	2,14
lmo2386	lmo2386	similar to B. subtilis YuiD protein	lin2485		3,63	
lmo2391	lmo2391	conserved hypothetical protein similar to B. subtilis YhfK protein	lin2490		4,56	
ltrC	lmo2398	low temperature requirement C protein, also similar to B. subtilis YutG protein	lin2497, ltrC		2,93	
lmo2672	lmo2672	weakly similar to transcription regulator				-2,31
rpiB-4	lmo2674	similar to ribose 5-phosphate epimerase B	lin2821			-2,68
lmo2696	lmo2696	similar to hypothetical dihydroxyacetone kinase	lin2844		2,50	
lmo2697	lmo2697	unknown protein	lin2845		3,14	
SigB down regulated during stationary growth phase						
ispE	lmo0190	4-diphosphocytidyl-2-C-methyl-D-erythritol kinase	lin0229		2,14	
ftsH	lmo0220	hypothetical protein. NOTE: highly similar to cell division protein ftsH	lin0252		7,94	2,95
lysS	lmo0228	lysyl-tRNA synthetase	lin0260, lysS		3,76	
iap	lmo0582	P60 extracellular protein, invasion associated protein Iap	lin0591, iap	34,54	28,64	2,04
lmo0663	lmo0663	hypothetical protein. NOTE: conserved hypothetical proteins	lin0668		2,89	
fabI	lmo0970	enoyl-(acyl carrier protein) reductase	lin0969		3,27	
ispD	lmo1086	2-C-methyl-D-erythritol 4-phosphate cytidylyltransferase	lin1071, ispD		2,66	
lmo1087	lmo1087	similar to glucitol dehydrogenase	lin1072	6,63	4,17	4,26
frr	lmo1314	highly similar to ribosome recycling factors	lin1351		2,68	
lmo1424	lmo1424	putative manganese transport protein MntH	lin1463		10,20	2,68
lmo1431	lmo1431	similar to ABC transporter (ATP-binding protein).	lin1470		3,46	
lmo1440	lmo1440	unknown proteins.	lin1479		2,43	
rpmA	lmo1540	50S ribosomal protein L27	lin1575, rpmA		3,94	
rplU	lmo1542	ribosomal protein L21	lin1577, rplU	4,72	8,11	2,22
mreD	lmo1546	similar to cell-shape determining protein MreD.	lin1581		2,45	
rpsD	lmo1596	30S ribosomal protein S4	lin1638, rpsD	7,36	14,32	3,07
tsf	lmo1657	elongation factor Ts. EF-Ts	lin1766, tsf		4,79	2,50
lmo1677	lmo1677	1,4-dihydroxy-2-naphthoate octaprenyltransferase	lin1785		3,18	
lmo1752	lmo1752	unknown protein	lin1864		3,43	
infC	lmo1785	translation initiation factor IF-3	lin1897, infC		5,70	2,06
rpe-2	lmo1818	ribulose-phosphate 3-epimerase	lin1932			-2,04
pyrE	lmo1831	orotate phosphoribosyltransferase	lin1945, pyrE			-3,56
lpeA	lmo1847	similar to adhesion binding proteins and lipoproteins with multiple specificity for metal cati	lin1961	6,54	31,12	10,63
lmo1848	lmo1848	similar metal cations ABC transporter (permease protein).	lin1962		9,92	2,87
lmo1849	lmo1849	similar to metal cations ABC transporter, ATP-binding proteins.	lin1963	3,34	17,88	5,58
lmo2048	lmo2048	unknown proteins.	lin2154		4,92	2,51
lmo2192	lmo2192	similar to oligopeptide ABC transporter (ATP-binding protein).	lin2296	7,78	7,21	
lmo2194	lmo2194	similar to oligopeptide ABC transporter (permease).	lin2298	3,36	3,36	2,48
fabF	lmo2201	similar to 3-oxoacyl-acyl-carrier protein synthase.	lin2304		3,92	2,03
lmo2254	lmo2254	unknown proteins.	lin2356		4,86	
pgi	lmo2367	glucose-6-phosphate isomerase	lin2466, pgi		5,82	2,23
lmo2415	lmo2415	similar to ABC transporter, ATP-binding protein.	lin2510			2,50
pgmA	lmo2456	phosphoglyceromutase	lin2550		6,28	
lmo2503	lmo2503	similar to cardiolipin synthase	lin2646		3,05	
spl	lmo2505	peptidoglycan lytic protein P45	lin2648, spl		12,21	2,68
ftsX	lmo2506	highly similar to cell-division protein FtsX.	lin2649			3,89
lmo2560	lmo2560	similar to B. subtilis RNA polymerase delta subunit.	lin2705			2,06
rpsI	lmo2596	30S ribosomal protein S9	lin2745, rpsI	3,56	9,71	
rplM	lmo2597	50S ribosomal protein L13	lin2746, rplM	5,03	12,82	3,29
rplV	lmo2627	50S ribosomal protein L22	lin2776, rplV	12,47	12,21	
rplB	lmo2629	50S ribosomal protein L2	lin2778, rplB			2,06
kat	lmo2785	catalase	lin2920, kat		6,45	2,68
**Class III**						
CtsR down regulated						
qoxB	lmo0014	AA3-600 quinol oxidase subunit I	lin0014, qoxB		2,42	
lmo0098	lmo0098	similar to PTS system mannose-specific, factor IID.	lin0145		3,00	
lmo0230	lmo0230	similar to B. subtilis YacH protein.	lin0262		2,87	
lmo0231	lmo0231	putative ATP:guanido phosphotransferase	lin0263	2,97	5,12	2,27
clpC	lmo0232	endopeptidase Clp ATP-binding chain C	lin0264, clpC	3,32	4,27	
lmo0496	lmo0496	similar to B. subtilis YnzC protein.	lin0497			-2,00
lmo0600	lmo0600	unknown protein	lin0609	4,64	5,63	
lmo0609	lmo0609	similar to E. coli phage shock protein E.	lin0618	13,83	6,76	
lmo0944	lmo0944	similar to B. subtilis YneR protein.	lin0943		2,93	
lmo0977	lmo0977	similar to B. subtilis YjcH protein.	lin0976		2,57	
guaA	lmo1096	bifunctional GMP synthase/glutamine amidotransferase protein	lin1081, guaA		2,72	
alsS	lmo2006	alpha-acetolactate synthase	lin2114		2,43	
lmo2114	lmo2114	similar to ABC transporter (ATP-binding protein).	lin2219		3,68	2,93
gpm	lmo2205	similar to phosphoglyceromutase 1.	lin2308	4,08	8,42	2,15
clpB	lmo2206	similar to endopeptidase Clp ATP-binding chain B (ClpB).	lin2309		3,90	
lmo2208	lmo2208	unknown protein.	lin2311		2,70	
