# Supplementary material for: In Vivo Transcriptional Profiling of Listeria monocytogenes and Mutagenesis Identify New Virulence Factors Involved in Infection
Source: PLoS Pathog. 2009 May 29;5(5):e1000449. doi: 10.1371/journal.ppat.1000449 (PMC2679221; doi:10.1371/journal.ppat.1000449)
Supplement: Table S8 — L. monocytogenes genes involved in metabolism and differentially regulated in the host (0.05 MB PDF) [file ppat.1000449.s010.pdf]

Table S8. *L. monocytogenes* EGDe genes involved in metabolism and differentially regulated in the host

Gene designation	Gene	Annotation
Genes up-regulated in vivo		
lmo0011	lmo0011	similar to mevalonate diphosphate decarboxylase
lmo0012	lmo0012	similar to mevalonate kinases
purA	lmo0055	highly similar to adenylosuccinate synthetase
lmo0098	lmo0098	similar to PTS system mannose-specific, factor IID
lmo0153	lmo0153	similar to a probable high-affinity zinc ABC transporter (Zn(II)-binding lipoprotein)
lmo0169	lmo0169	similar to a glucose uptake protein
plcA	lmo0201	phosphatidylinositol-specific phospholipase c
plcB	lmo0205	phospholipase C
sul	lmo0224	highly similar to dihydropteroate synthases
folA	lmo0225	highly similar to dihydroneopterin aldolase
folK	lmo0226	similar to 7,8-dihydro-6-hydroxymethylpterin pyrophosphokinase
lmo0231	lmo0231	similar to arginine kinase
lmo0265	lmo0265	similar to succinyldiaminopimelate desuccinylase
lmo0278	lmo0278	similar to sugar ABC transporter, ATP-binding protein
lmo0279	lmo0279	highly similar to anaerobic ribonucleoside-triphosphate reductase
lmo0280	lmo0280	highly similar to anaerobic ribonucleotide reductase activator protein
lmo0301	lmo0301	similar to PTS beta-glucoside-specific enzyme IIA component
lmo0366	lmo0366	conserved hypothetical protein, putative lipoprotein
lmo0541	lmo0541	similar to ABC transporter (binding protein)
lmo0580	lmo0580	weakly similar to carboxylesterase
lmo0593	lmo0593	similar to transport proteins (formate?)
lmo0594	lmo0594	similar to homoserine O-acetyltransferase
lmo0607	lmo0607	similar to ABC transporter, ATP-binding protein
lmo0608	lmo0608	similar to ABC transporter, ATP-binding protein
lmo0645	lmo0645	similar to amino acid transporter
lmo0650	lmo0650	conserved membrane protein
thiD	lmo0662	highly similar to phosphomethylpyrimidine kinase thiD
lmo0781	lmo0781	similar to mannose-specific phosphotransferase system (PTS) component IID
lmo0782	lmo0782	similar to mannose-specific phosphotransferase system (PTS) component IIC
lmo0783	lmo0783	similar to mannose-specific phosphotransferase system (PTS) component IIB
lmo0784	lmo0784	similar to mannose-specific phosphotransferase system (PTS) component IIA
uhpT	lmo0838	highly similar to hexose phosphate transport protein
lmo0841	lmo0841	similar to cation (calcium) transporting ATPase
lmo0848	lmo0848	similar to amino acid ABC transporter, ATP-binding protein
lmo0876	lmo0876	similar to PTS system, Lichenan-specific enzyme IIC component
lmo0915	lmo0915	similar to phosphotransferase system enzyme IIC
lmo0970	lmo0970	similar to enoyl- acyl-carrier protein reductase
lmo0978	lmo0978	similar to branched-chain amino acid aminotransferase
ptsH	lmo1002	PTS phosphocarrier protein Hpr (histidine containing protein)
lmo1003	lmo1003	phosphotransferase system enzyme I
lmo1006	lmo1006	similar to aminotransferases (to B. subtilis PatA protein)
lmo1011	lmo1011	similar to tetrahydrodipicolinate succinylase
lmo1086	lmo1086	similar to CDP-ribitol pyrophosphorylase
lmo1087	lmo1087	similar to glucitol dehydrogenase
lmo1093	lmo1093	similar to NH(3)-dependent NAD(+) synthetases, nitrogen regulatory protein
guaA	lmo1096	highly similar to GMP synthetase
lmo1207	lmo1207	similar to cobalt transport ATP-binding protein CbiO
lmo1250	lmo1250	similar to antibiotic resistance protein
glnA	lmo1299	highly similar to glutamine synthetases
lmo1354	lmo1354	similar to aminopeptidase P
tcsA	lmo1388	CD4+ T cell-stimulating antigen, lipoprotein
lmo1424	lmo1424	similar to manganese transport proteins NRAMP
opuCD	lmo1425	similar to betaine/carnitine/choline ABC transporter (membrane p)
lmo1431	lmo1431	similar to ABC transporter (ATP-binding protein)
udk	lmo1497	similar to Uridine kinase
lmo1536	lmo1536	similar to prephenate dehydratase PheA
lmo1578	lmo1578	similar to X-Pro dipeptidase
lmo1592	lmo1592	similar to thiamin biosynthesis protein ThiI
aroA	lmo1600	3-deoxy-D-arabino-heptulosonate 7-phosphate synthase
menD	lmo1675	similar to 2-succinyl-6-hydroxy-2,4-cyclohexadiene-1-carboxylate synthase / 2-oxoglutarate decarboxylase
lmo1677	lmo1677	similar to menaquinone biosynthesis proteins
lmo1705	lmo1705	similar to deoxyguanosine kinase/deoxyadenosine kinase(I) subunit
lmo1711	lmo1711	highly similar to aminopeptidases
adeC	lmo1742	highly similar to adenine deaminases
lmo1761	lmo1761	similar to putative sodium-dependent transporter
purD	lmo1764	phosphoribosylglycinamide synthetase
lmo1780	lmo1780	similar to aminotripeptidase (peptidase T)
lmo1847	lmo1847	similar to adhesion binding proteins and lipoproteins with multiple specificity for metal cations (ABC transporter)
lmo1848	lmo1848	similar metal cations ABC transporter (permease protein)
lmo1849	lmo1849	similar to metal cations ABC transporter, ATP-binding proteins
ilvN	lmo1985	similar to acetolactate synthase (acetohydroxy-acid synthase) (small subunit)
nadC	lmo2024	similar to nicotinate-nucleotide pyrophosphorylase
nadA	lmo2025	similar to quinolinate synthetase
ctaA	lmo2058	similar to heme O oxygenase
lmo2077	lmo2077	similar to glycoprotease
lmo2101	lmo2101	similar to a protein required for pyridoxine synthesis
lmo2114	lmo2114	similar to ABC transporter (ATP-binding protein)
lmo2115	lmo2115	similar to ABC transporter (permease)
lmo2154	lmo2154	similar to ribonucleoside-diphosphate reductase, subunit beta
lmo2155	lmo2155	similar to ribonucleoside-diphosphate reductase, subunit alpha
lmo2182	lmo2182	similar to ferrichrome ABC transporter (ATP-binding protein)
lmo2184	lmo2184	similar to ferrichrome ABC transporter (binding protein)
lmo2188	lmo2188	similar to oligoendopeptidase
lmo2192	lmo2192	similar to oligopeptide ABC transporter (ATP-binding protein)
lmo2193	lmo2193	similar to oligopeptide ABC transporter (ATP-binding protein)
lmo2194	lmo2194	similar to oligopeptide ABC transporter (permease)
lmo2195	lmo2195	similar to oligopeptide ABC transporter (permease)
lmo2196	lmo2196	similar to pheromone ABC transporter (binding protein)
lmo2201	lmo2201	similar to 3-oxoacyl-acyl-carrier protein synthase
lmo2202	lmo2202	similar to 3-oxoacyl- acyl-carrier protein synthase
hemH	lmo2211	similar to ferrochelatase

lmo2215	lmo2215	similar to ABC transporter (ATP-binding protein)
lmo2251	lmo2251	similar to amino acid ABC transporter (ATP-binding protein)
lmo2413	lmo2413	similar to aminotransferase
lmo2414	lmo2414	similar to aminotransferase
lmo2415	lmo2415	similar to ABC transporter, ATP-binding protein
lmo2503	lmo2503	similar to cardiolipin synthase
cydD	lmo2715	highly similar to ABC transporter (ATP-binding protein) required for expression of cytochrome BD
lmo2770	lmo2770	similar to gamma-glutamylcysteine synthetase (N_terminal part) and to cyanophycin synthetase (C-terminal part)
Genes down-regulated in vivo		
lmo0023	lmo0023	similar to PTS system, fructose-specific IIC component
lmo0024	lmo0024	similar to PTS system, mannose-specific IID component
lmo0033	lmo0033	similar to endoglucanase
lmo1142	lmo1142	similar to Salmonella enterica PduS protein
lmo1143	lmo1143	similar to Salmonella enterica PduT protein
lmo1144	lmo1144	similar to Salmonelle enterica PduU protein
lmo1145	lmo1145	similar to Salmonella enterica PduV protein
lmo1147	lmo1147	similar to bifunctional cobalamin biosynthesis protein CopB
lmo1148	lmo1148	highly similar to cobalamin (5'-phosphatase) synthetase
lmo1149	lmo1149	similar to alpha-ribazole-5'-phosphatase
lmo1151	lmo1151	similar to Salmonella typhimurium PduA protein
lmo1152	lmo1152	similar to Salmonella typhimurium PduB protein
lmo1153	lmo1153	highly similar to propanediol dehydratase, alpha subunit
lmo1154	lmo1154	similar to diol dehydrase (diol dehydratase) gamma subunit
lmo1156	lmo1156	similar to diol dehydratase-reactivating factor large subunit
lmo1157	lmo1157	similar to diol dehydratase-reactivating factor small chain
lmo1158	lmo1158	similar to Salmonella enterica PduK protein
lmo1159	lmo1159	similar to carboxysome structural protein
lmo1160	lmo1160	similar to Salmonella enterica PduL protein
lmo1161	lmo1161	similar to ethanolamine utilization protein EutJ
lmo1164	lmo1164	hyghly similar to Salmonella enterica PduO protein
lmo1165	lmo1165	similar to ethanolamine utilization protein EutE
lmo1166	lmo1166	similar to NADPH-dependent butanol dehydrogenase
glpF	lmo1167	similar to glycerol uptake facilitator protein
AckA2	lmo1168	similar to acetate kinase
cobD	lmo1169	similar to Salmonella typhimurium CobD protein and to histidinol-phosphate aminotransferase
pduQ	lmo1171	similar to NADPH-dependent butanol dehydrogenase
lmo1173	lmo1173	similar to two-component sensor histidine kinase
eutA	lmo1174	similar to ethanolamine utilization protein EutA (putative chaperonin)
eutB	lmo1175	similar to ethanolamine ammonia-lyase, heavy chain
eutC	lmo1176	similar to ethanolamine ammonia-lyase, light chain
lmo1177	lmo1177	similar to putative carboxysome structural protein (eutL)
lmo1180	lmo1180	similar to putative carboxysome structural protein
lmo1181	lmo1181	similar to cobalamin adenosyl transferase
lmo1182	lmo1182	similar to Salmonella enterica PduL protein
cbiA	lmo1191	similar to cobyrinic acid a,c-diamide synthase
lmo1192	lmo1192	similar to cobalamine synthesis protein CbiB
cbiD	lmo1194	similar to cobalamin biosynthesis protein CbiD
lmo1728	lmo1728	some similarities to cellobiose-phosphorylase
lmo1812	lmo1812	similar to L-serine dehydratase
lmo1817	lmo1817	weakly similar to thiamin pyrophosphokinase
lmo1818	lmo1818	similar to ribulose-5-phosphate 3-epimerase (PPP)
lmo1825	lmo1825	similar to pantothenate metabolism flavoprotein homolog
pyrE	lmo1831	highly similar to orotate phosphoribosyltransferases
pyrF	lmo1832	highly similar to orotidine 5'-phosphate decarboxylases
pyrD	lmo1833	highly similar to dihydroorotase dehydrogenase
pyrDII	lmo1834	highly similar to dihydroorotate dehydrogenase (electron transfer subunit)
pyrAB	lmo1835	highly similar to carbamoyl-phosphate synthetase (catalytic subunit)
pyrAa	lmo1836	highly similar to carbamoyl-phosphate synthetase (glutaminase subunit)
pyrC	lmo1837	highly similar to dihydroorotase
pyrP	lmo1839	highly similar to uracil permease
lmo1851	lmo1851	similar to carboxy-terminal processing proteinase
lmo1871	lmo1871	similar to phosphoglucomutases
lmo1883	lmo1883	similar to chitinases
lmo2175	lmo2175	similar to dehydrogenase
lmo2425	lmo2425	similar to glycine cleavage system protein H
lmo2586	lmo2586	similar to formate dehydrogenase alpha chain
lmo2648	lmo2648	similar to Phosphotriesterase
lmo2649	lmo2649	similar to hypothetical PTS enzyme IIC component
lmo2657	lmo2657	similar to dGTP triphosphohydrolase
lmo2660	lmo2660	similar to transketolase (PPP)
lmo2661	lmo2661	similar to ribulose-5-phosphate 3-epimerase (PPP)
lmo2662	lmo2662	similar to ribose 5-phosphate epimerase (PPP)
lmo2663	lmo2663	similar to polyol dehydrogenase
lmo2664	lmo2664	similar to sorbitol dehydrogenase
lmo2665	lmo2665	similar to PTS system galactitol-specific enzyme IIC component
lmo2674	lmo2674	similar to ribose 5-phosphate epimerase (PPP)
kdpB	lmo2681	potassium-transporting atpase b chain
lmo2733	lmo2733	similar to PTS system, fructose-specific IIABC component
lmo2741	lmo2741	similar to drug-efflux transporters
lmo2761	lmo2761	similar to beta-glucosidase
lmo2763	lmo2763	similar to PTS cellobiose-specific enzyme IIC
