# Supplementary material for: In Vivo Transcriptional Profiling of Listeria monocytogenes and Mutagenesis Identify New Virulence Factors Involved in Infection
Source: PLoS Pathog. 2009 May 29;5(5):e1000449. doi: 10.1371/journal.ppat.1000449 (PMC2679221; doi:10.1371/journal.ppat.1000449)
Supplement: Table S9 — Primers (0.04 MB PDF) [file ppat.1000449.s011.pdf]

Table S9 - Primers

Primers used for construction of mutants

Name	Sequence (5' to 3')	Restriction site
lmo1081-F	G*GAGCTC* TGATTTATTATCCGCTATCG	SacI
lmo1081-R	ATG*GTCGAC* CAATTTCCTCAAGACAAGCG	SalI
lmo1082-A	TAC*GTCGAC* TGCTCAAATCGATGCTGG	SalI
lmo1082-B	CG*ACGCGT* CATTCTTTTCTCTCC	MluI
lmo1082-C	CG*ACGCGT* TAAATGAATTTATTAG	MluI
lmo1082-D	TAC*AGATCT* TAGTGGTCTCCACCAAGC	BglII
lmo1102-A	CGA*GTCGAC* TTCCCACTATCAAAGTGG	SalI
lmo1102-B	C*GAATTC* CACGTTCCTAAACACTCC	EcoRI
lmo1102-C	C*GAATTC* TAAAAAAAATCTTCAAACAC	EcoRI
lmo1102-D	CGC*AGATCT* AATTCGTACAAGACATACC	BglII
lmo2219-F	G*GAGCTC* AAAGACGAACTTTATGACGC	SacI
lmo2219-R	ATG*GTCGAC* TGAGACTCAAGGTAAGAAGC	SalI
lmo2459-A	G*GAATTC* ATGACAGTTAAAGTTGGTATTAATGG	EcoRI
lmo2459-B	AC*GGTACC* TTACGCCCCTAAAGAGAACACGCCAATAGCTAACATTGCAAGAATTAATTATTTAGCGATTTTTGC	KpnI
lmo2459-C	G*TCTAGA* GAAGTGCTAGTCACATCAGC	XbaI
lmo2459-D	ATG*CTGCAG* AGTTCGAATACGCCCATTGG	PstI
lmo2713-F	C*GAATTC* TAAGTACCTCGGTTCAGC	EcoRI
lmo2713-R	ATA*GTCGAC* AGCGACGCAACCTAATGTG	SalI
lmo2714-F	G*GAATTC* GACAGAACCAGCAGAACC	EcoRI
lmo2714-R	ATA*GTCGAC* TGGAGCTGTTACGACTGC	SalI

Primers used for verification of mutagenesis

Name	Sequence (5' to 3')
lmo1081-F	CCATTCGCCATTCAGGCTGCG
lmo1081-R	TACAGATCTTAGTGGTCTCCACCAAGC
lmo1082-F	CCACTCTCTTTCTGTGGAAGC
lmo1082-R	ATCATAAATTGCGCCTGTCG
lmo1102-F	AAGCCCTATCTGAAGAAACTAGG
lmo1102-R	CGCTACTGTTGATTTCACAATG
lmo2219-F	CCATTCGCCATTCAGGCTGCG
lmo2219-R	CGCAGATCTGTTGGAGAAACAGTAGACC
lmo2713-F	CCATTCGCCATTCAGGCTGCG
lmo2713-R	AGCTCTCCATTACTTCCAACC
lmo2714-F	CCATTCGCCATTCAGGCTGCG
lmo2714-R	AGTAGGTTGTTGCGGTAGTGC

Primers used for construction of complemented strains

Name	Sequence (5' to 3')	Restriction site
lmo1082-G	CG*ACTAGT* AGCGTTACCTTGAACTGGGAG	SpeI
lmo1082-H	ATA*GTCGAC* CAATAAAACCTGCCCCCACCTG	SalI
lmo1102-G	GCC*ACTAGT* AACCCTGTTTACACACAAGC	SpeI
lmo1102-H	ACC*GTCGAC* TAGTCCGTGACTCTTTAGAC	SalI
lmo2714-G	AGT*CTGCAG* TAATGGCAGAAGAACCACGC	PstI
lmo2714-H	AGT*GTCGAC* AAAGGAGTGACCAAGTAGGC	SalI
lmo2459-G	ATA*CTGCAG* CTTACGATTAGTACAATCCG	PstI
lmo2459-H	ATA*GTCGAC* TAGCCATTTAGAGTTCCTCC	SalI

Primers used for qPCR

Name	Sequence (5' to 3')
lmo0206-F	CCATTTCATTTAAGCGATGC
lmo0206-R	TCCAGCTATATCACCCTTTTCC
lmo1081-F	CTTATGGGCAGAGGATTTGCT
lmo1081-R	GCAGCCTCATCAATATACCC
lmo1082-F	CCACTCTCTTTCTGTGGAAGC
lmo1082-R	ATCATAAATTGCGCCTGTCG
lmo1102-F	AAGCCCTATCTGAAGAAACTAGG
lmo1102-R	CGCTACTGTTGATTTCACAATG
lmo1290-F	TTCAGCTTGATGACGGAACG
lmo1290-R	CCGTCGCTTGTAATTGTTAGGC
lmo2219-F	CAATACGGATACCACATCATCC
lmo2219-R	TGTCTTCCACTTTCACGTTAGC
lmo2713-F	AAAGCACTACCGCAACAACC
lmo2713-R	CCATTTGCCGATATGACTGG
lmo2714-F	TGGAGAAGGAACAACAACTGC
lmo2714-R	TTTCGCTGGGACTTGATAGG
fri-F	ACTAGCAATCGGCGGAAGC
fri-R	TCGCCTTCTTTGTCAGTAAGC
oppA-F	AGCGCACTAATTCCTTCTGC
oppA-R	GTCAAGCGCATATAGACCTTCG
sigB-F	AGAAACGGGTGAACTACTCG
sigB-R	CTTCCTCATTCTGCAACGCC
inlA-F	ACAACTGGAGGGAACGCGCC
inlA-R	CCAGGTATATTTGCGGAAGG
prfA-F	TCATTAGCGAGCAGGCTACC
prfA-R	GCAAATAGAGCCAAGCTTCC
16S-F	CTCGTGTCGTGAGATGTTGG
16S-R	CGTGTGTAGCCCAGGTCATA
hly-F	CGTCCATCTATTTGCCAGGT
hly-R	ATTTCGGATAAAGCGTGGTG
murA-F	AGCGCAGACGAAACAGCGCC
murA-R	AGGAGTGGCCGTTGCTGATGC
inlH-F	CG CAA ATT ACA GAT GTG ACC
inlH-R	TT AGT TAG TCC TGC AAG CGG
sod-F	GCTGGTCATCCTGAACTTGC
sod-R	CCACCGTGGTTACGGACAGC
ami-F	TGCTGCTTCCATTGATCCGGTGC
ami-R	GCGCGAAGCAAAGGGACTTGC
lpeA-F	CCAGCCAACATTCAAAGTGC
lpeA-R	CGATCAAACCAACCGTTCCC
iap-F	GCGCCAACAATCGCATCCGC
iap-R	TCAACACCAGCGCCACTACGG
gap-F	CTGCTCAACGTGTTCCAGTTCC
gap-R	GCTTCCATAGCTGCATTTACTTCG
